# Plasma proteomics uncovers divergent molecular signatures in ischemic stroke and intracerebral hemorrhage

**DOI:** 10.1186/s40364-025-00848-1

**Published:** 2025-10-28

**Authors:** David Núñez-Jurado, Alejandro Fernández-Vega, Carmen del Río, Anna Penalba, Laia Llucià-Carol, Elena Muiño-Acuña, Garbiñe Ezcurra-Díaz, Marina Guasch-Jiménez, Natalia Cullell, Gemma Serrano-Heras, Lourdes Arias-Salazar, Cristòfol Vives-Bauza, Silvia Tur, Xabier Urra, Mar Castellanos, Jerzy Krupinski, Marimar Freijo-Guerrero, Jordi Jiménez-Conde, Isabel Fernández-Pérez, Tomás Segura, Joan Marti-Fabregas, Israel Férnandez-Cadenas, Joan Montaner

**Affiliations:** 1 Neurovascular Research Group, Institute de Biomedicine of Seville, IBiS/Virgen Macarena University Hospital/CSIC/University of Seville, Av. Dr. Fedriani, 3, Seville, 41009 Spain; 2 Department of Clinical Biochemistry, Virgen Macarena University Hospital, Seville, 41009 Spain; 3 Department of Cell Biology, University of Seville, Seville, 41013 Spain; 4 Neurovascular Research Laboratory, Vall d’Hebron Institute of Research (VHIR), Universitat Autónoma de Barcelona, Barcelona, 08035 Spain; 5 Stroke Pharmacogenomics and Genetics Group, Institut de Recerca Sant Pau (IR SANT PAU), Barcelona, 08041 Spain; 6 Departament de Genètica i de Microbiologia, Universitat Autònoma de Barcelona, Barcelona, 08193 Spain; 7 Stroke Unit, Department of Neurology, Hospital de la Santa Creu i Sant Pau, Institut de Recerca Sant Pau (IR SANT PAU), Barcelona, 08025 Spain; 8 Stroke Pharmacogenomics and Genetics Laboratory, Fundación Docència I Recerca Mútua Terrassa, Hospital Mútua Terrassa, Barcelona, 08221 Spain; 9 Research Unit, Albacete University Hospital Complex (CHUA), Albacete, 02008 Spain; 10 Department of Neurology, Hospital Universitario Son Espases (HUSE), Mallorca, 07120 Spain; 11 Functional Unit of Cerebrovascular Diseases, Institute of Neurosciences, Hospital Clinic-Institut d’Investigacions Biomèdiques August Pi i Sunyer (IDIBAPS), University of Barcelona, Barcelona, 08036 Spain; 12 Department of Neurology, Health Sciences Faculty, Biomedical Research Institute, University of A Coruña, A Coruña, 15006 Spain; 13 Stroke Unit of the Neurology Service, Cruces University Hospital, Biobizkaia Health Research Institute, Barakaldo, 48903 Spain; 14 Department of Neurology, Neurovascular Research Group, Instituto de investigaciones médicas Hospital del Mar (IMIM) Hospital del Mar, Barcelona, 08003 Spain; 15 Department of Neurology, General University Hospital of Albacete, Albacete, 02006 Spain

**Keywords:** Stroke, Proteomics, Biomarkers, Neuroinflammation

## Abstract

**Background:**

Timely differentiation between ischemic stroke (IS) and intracerebral hemorrhage (ICH) is critical for guiding appropriate acute management strategies. While neuroimaging is the diagnostic gold standard, its accessibility is often limited in urgent clinical settings. Blood biomarkers offer a promising, scalable diagnostic alternative; however, no validated panel is yet available for distinguishing stroke subtypes during the hyperacute phase.

**Methods:**

In a multicenter study, plasma samples were collected within 6 h of symptom onset. A total of 3,072 proteins were measured using Olink® proximity extension assays. We applied differential expression analysis, principal component analysis (PCA), partial least squares discriminant analysis (PLS-DA), and receiver operating characteristic (ROC) curve evaluation. To interpret the biological relevance of the findings, we conducted functional enrichment and protein–protein interaction (PPI) analyses.

**Results:**

Among the 388 patients (344 IS, 44 ICH), 2,531 proteins were retained; 878 reached nominal significance (*p* < 0.05), and 67 remained significant after multiple-testing correction (FDR-adjusted *p* < 0.05). Of these, 844 were overexpressed in ICH and 34 in IS. GFAP, a glial marker, emerged as the most discriminative biomarker for ICH versus IS (AUC = 0.887; sensitivity: 80%, specificity: 90%), followed by BCAN (AUC = 0.820), SNAP25 (AUC = 0.797), and SPOCK1 (AUC = 0.786). For IS, S100A12 (AUC = 0.677) and MNDA (AUC = 0.657) showed the best performance. Multivariate analyses confirmed the presence of distinct proteomic patterns, with enrichment revealing a significant overrepresentation of neurodevelopmental and synaptic pathways. In PPI networks, GFAP and LYN emerged as central hubs.

**Conclusion:**

This study reveals a robust plasma proteomic signature distinguishing IS from ICH within hours of onset. These results lay the groundwork for scalable, blood-based diagnostics to guide early stroke management when imaging is delayed or unavailable.

**Supplementary Information:**

The online version contains supplementary material available at 10.1186/s40364-025-00848-1.

## Highlights

- GFAP shows high accuracy in differentiating between ischemic stroke and intracerebral hemorrhage.

- GFAP and LYN are central hubs in intracerebral hemorrhage-related protein networks.

- Neuroinflammatory and synaptic remodeling proteins dominated intracerebral hemorrhage.

- S100A12 and MNDA were key discriminative proteins in ischemic stroke.

**Supplementary Information:**

The online version contains supplementary material available at 10.1186/s40364-025-00848-1.

## Introduction

Stroke is a major contributor to global disability-adjusted life years and healthcare burden, with distinct therapeutic paths depending on subtype [1]. Stroke is generally classified into two main types: ischemic stroke (IS), accounting for ~ 80% of cases, and intracerebral hemorrhage (ICH), comprising the remaining 20% [2]. IS is caused by the occlusion of a cerebral blood vessel, usually due to thrombosis or embolism, leading to a reduction in blood flow and oxygen supply to brain tissue [3]. In contrast, ICH results from the rupture of a blood vessel, causing bleeding into the brain parenchyma or surrounding spaces, which leads to increased intracranial pressure and direct tissue damage [4]. Early differentiation between IS and ICH is critical, given the markedly different therapeutic approaches required for each. While IS requires urgent reperfusion therapies such as intravenous thrombolysis or mechanical thrombectomy, ICH management focuses on blood pressure control, reversal of anticoagulation, and, in selected cases, surgical intervention [5].

In clinical practice, the differential diagnosis between IS and ICH relies on neuroimaging, particularly non-contrast cranial computed tomography (CT), due to its availability and speed [6]. However, this approach has important limitations. In early-stage IS, CT scans may not reveal clear abnormalities due to their limited sensitivity to subtle signs, including hypoattenuation or loss of gray-white matter differentiation, potentially delaying treatment [7, 8]. Additionally, in settings with limited access to urgent imaging, such as rural hospitals or low-resource countries, delays in performing CT scans or the absence of specialized personnel may compromise early diagnosis [9]. Clinical presentation alone is frequently unreliable, as IS and ICH can present with identical symptoms, which may overlap with stroke mimics and non-vascular conditions, further complicating timely diagnosis that could even guide patient transfer decisions [10]. These challenges underscore the need for complementary tools that can enhance early differential diagnosis, particularly in prehospital settings or during the first minutes after symptom onset.

Given the limitations of current diagnostic tools, there is growing interest in identifying blood-based biomarkers that can aid in the early differentiation between IS and ICH [11]. Notably, glial fibrillary acidic protein (GFAP) emerges as a strong candidate due to its markedly higher concentrations in ICH relative to IS [12]. Conversely, biomarkers such as N-terminal pro-B-type natriuretic peptide (NT-proBNP) have been associated with IS and are increasingly being studied for their diagnostic and prognostic value [13]. However, no single biomarker has yet demonstrated sufficient accuracy to be adopted widely in clinical practice. Proteomics, the large-scale study of proteins, offers a powerful and unbiased approach for the discovery of novel biomarkers by allowing comprehensive profiling of the protein content in biological fluids [14]. The proximity extension assay (PEA) technology, known for its excellent reproducibility and stability, has been increasingly utilized due to its various assay panels targeted towards different diseases [15]. This approach may enable the identification of protein signatures specific to stroke subtypes, potentially enhancing diagnostic precision and informing early intervention strategies.

The objective of this study was to apply a proteomic approach to plasma samples obtained during the hyperacute phase of stroke to identify distinct molecular signatures capable of differentiating IS from ICH. By characterizing the circulating protein profiles associated with each subtype, the study aimed to discover candidate biomarkers to support early differential diagnosis, especially in contexts where imaging is not readily available, and to gain insight into the underlying pathophysiological mechanisms, ultimately contributing to improved clinical decision-making and patient outcomes.

## Materials and methods

### Ethics statement

This study was performed following the ethical recommendations contained in the Declaration of Helsinki on Humans [16] and in the International Council for Harmonisation (ICH) E6 Guidelines on Good Clinical Practice [17]. The protocol was reviewed and approved by the Institutional Research Ethics Committee of the authors’ institution (IIBSP-PRE-2022-39). Written informed consent was obtained from all participants or their legal representatives where applicable prior to the initiation of any study-related procedures. All personal data were handled in strict compliance with applicable data protection regulations to ensure participant confidentiality and anonymity. Furthermore, this manuscript adheres to the relevant Strengthening the Reporting of Observational studies in Epidemiology (STROBE) guidelines.

### Study design and patients

We conducted a prospective, multicenter observational study across nine Spanish hospitals from September 2022 to December 2024. The study was designed to evaluate plasma protein profiles in patients presenting with acute stroke, with the objective of identifying potential biomarkers capable of distinguishing between ischemic and hemorrhagic subtypes. The participating centers included: Hospital de la Santa Creu i Sant Pau, Hospital Clínic of Barcelona, Hospital del Mar, Vall d’Hebron University Hospital, and Mútua Terrassa University Hospital in Barcelona; University Hospital of A Coruña in A Coruña; Son Espases University Hospital in Palma de Mallorca; University Hospital Complex of Albacete in Albacete; and Virgen Macarena University Hospital in Seville.

Eligible patients were adults (aged ≥ 18 years) who presented to the emergency department with symptoms suggestive of acute stroke and received a confirmed diagnosis of either IS or ICH by neuroimaging. Stroke diagnosis was based on World Stroke Organization (WSO) criteria [18] and confirmed through non-contrast cranial CT or MRI at admission, interpreted by stroke specialists at each site. IS was defined by radiological evidence of vessel occlusion or infarction; ICH was defined as primary intracerebral hemorrhage, with or without intraventricular or subarachnoid extension. Patients were consecutively enrolled within the hyperacute phase, defined for this study as ≤ 6 h from symptom onset or last known well time, and plasma samples were obtained before any reperfusion therapy or surgical intervention.

Upon hospital arrival, stroke severity was assessed using the National Institutes of Health Stroke Scale (NIHSS), and all patients underwent standard clinical evaluations including medical history, neurological examination, and routine laboratory investigations. Patients with transient ischemic attacks, stroke mimics, or uncertain stroke subtype diagnosis were excluded. Additional exclusion criteria included prehospital diagnoses other than stroke, inability to obtain blood samples within the inclusion window, refusal to provide informed consent, active cancer, recent major surgery or trauma, ongoing systemic infection, or other conditions known to significantly alter systemic protein expression profiles.

### Sample collection and processing

Peripheral blood samples were collected specifically for this study via venipuncture into ethylenediaminetetraacetic acid (EDTA) tubes (BD Vacutainer^®^ K2EDTA spray-coated) during the hyperacute phase of stroke, following standard aseptic procedures. All samples were obtained prior to the initiation of any reperfusion therapy or surgical intervention to avoid treatment-related alterations in protein expression profiles. Immediately after collection, samples were centrifuged at 1,500 g for 15 min at 4 °C, and plasma was promptly inspected for preanalytical quality. In cases of visible hemolysis, a new blood sample was immediately obtained. For lipemic plasma, an additional centrifugation at 10,000 g for 5 min was performed to remove the lipid layer, retaining the clear infranatant. The processed plasma was then aliquoted and stored at − 80 °C until further analysis. All frozen plasma aliquots were shipped on dry ice to Olink^®^ Proteomics (Uppsala University, Sweden) under strict cold-chain conditions, ensuring they remained at − 80 °C during transport. Upon arrival, sample integrity was verified before proteomic analysis.

To minimize technical variability and potential biases, all samples were processed under blinded conditions. Specifically, each specimen was assigned a unique identifier, and technicians performing the proteomic assays had no access to patients’ demographic or clinical data, including stroke subtype. Randomization was applied during processing to avoid batch effects, and anonymity and traceability were ensured throughout all study procedures. Consistency across assay panels was maintained through regular quality control and calibration procedures in accordance with the manufacturer’s guidelines. These measures ensured high analytical reliability and that observed differences in protein expression reflected true biological variation rather than methodological artifacts.

### Proteomic analysis

Proteomic analysis was conducted at Olink® Proteomics (Uppsala University, Sweden) PEA platform [19]. This method uses pairs of oligonucleotide-labeled antibodies that bind specifically to target proteins; when both antibodies bind in close proximity, their oligonucleotides hybridize and are extended by a DNA polymerase, creating a unique sequence that is subsequently amplified and quantified by real-time PCR. Results were reported in Normalized Protein eXpression (NPX) units, presented on a log2 scale and reflecting relative — rather than absolute — quantities. While NPX values cannot be directly converted to standard concentration units, a higher NPX value corresponds to a higher protein expression level. Importantly, NPX values are comparable only for the same protein within the same project run, which is essential for ensuring the validity of comparative analyses.

To ensure analytical robustness and minimize variability, all plasma samples were run in singleton as per the manufacturer’s guidelines. All samples were processed within the same run and plate to reduce both intra- and inter-assay variation. Quality control procedures included the incorporation of four internal controls spiked into every sample. Each sample plate also contained six mandatory and two optional external controls placed in separate wells, which served to monitor assay performance and validate run consistency.

A total of 3,072 proteins were measured using eight Olink® panels (Neurology I and II Panels, Inflammation I and II Panels, Cardiometabolic I and II Panels and Oncology I and II Panels), each comprising 384 biomarkers. These panels were selected to maximize biological coverage relevant to stroke pathophysiology, including neurological, inflammatory, cardiovascular, metabolic, and oncological pathways:

### Preliminary data analysis

Prior to conducting the primary statistical analyses, a series of pre-processing steps were performed to ensure data quality and to appropriately manage values falling below the assay’s limit of detection (LOD). The handling of proteins with missing or undetectable values was systematically guided by the proportion of affected samples per protein and group:

- Proteins with a low proportion of undetectable values (< 30% of samples below LOD or with missing values per clinical group, equivalent to fewer than three individuals per group) were retained for the main differential expression analyses. These proteins were considered robust and likely to yield meaningful biological insights.
- Proteins with intermediate levels of missingness (> 30% of samples below LOD per group but < 70% across the entire dataset) were flagged as potentially “on-off” markers. These proteins might show activation or detection based on patient characteristics within each group. Although excluded from the main differential expression analysis, an alternative analysis using Fisher’s exact test was performed to evaluate whether the distribution of missing or undetectable values was associated with stroke subtype.
- Proteins with extensive missingness (> 70% of all samples below LOD) were excluded from further analysis due to the limited interpretability and potential unreliability of their expression patterns.

### Statistics

Statistical analyses were performed using R software (version 4.4.2) with associated packages and MedCalc 14.0 statistical software (MedCalc Software, Ostend, Belgium). Descriptive statistics were computed to summarize demographic and clinical variables. Continuous variables were presented as median and interquartile range (IQR), and categorical variables as frequencies and percentages. Group comparisons between IS and ICH were conducted using the Mann–Whitney U test for continuous variables and the chi-square test for categorical variables, as appropriate. A two-sided *p*-value < 0.05 was considered statistically significant.

For the proteomic data, initial pre-processing included quality control and filtering of proteins based on missing values and detection limits, as described in the Methods section. Proteins with more than 70% missing or below LOD values across all samples were excluded. Differential expression analysis between stroke subtypes was conducted using linear models fitted with the limma package. *P*-values were adjusted for multiple comparisons using the Benjamini–Hochberg method to control the false discovery rate (FDR). Proteins were considered significantly differentially expressed if they met an adjusted *p*-value (FDR < 0.05). Results were visualized using volcano plot, where the –log10(adjusted *p*-value) was plotted against the log2 fold change, highlighting the most discriminative proteins.

Multivariate analysis was conducted to explore global protein expression patterns and their capacity to discriminate between IS and ICH. Principal Component Analysis (PCA) and Partial Least Squares Discriminant Analysis (PLS-DA) were performed using MetaboAnalyst 6.0. VIP (Variable Importance in Projection) scores were also calculated in MetaboAnalyst to identify proteins contributing most to group separation. Proteins with VIP > 1 were considered relevant. Heatmaps of differentially expressed proteins were generated to visualize expression patterns across samples and support group-level clustering, also using MetaboAnalyst 6.0.

Functional enrichment analysis of the differentially expressed proteins was carried out using STRING (version 12.0), including Gene Ontology (GO) terms for biological process and cellular component categories, as well as tissue-specific expression enrichment. Significantly enriched terms were identified based on FDR values and visualized using dot plots. Protein–protein interaction (PPI) networks were constructed separately using STRING (version 11.0), with interaction confidence thresholds set to medium (score > 0.4). Network visualization enabled the exploration of functional clustering and connectivity among significant proteins.

To assess diagnostic performance, Receiver Operating Characteristic (ROC) curve analysis was performed for each differentially expressed protein. The area under the curve (AUC), 95% confidence intervals (CIs), and optimal cut-off points were estimated using the pROC package in R and MetaboAnalyst 6.0. Proteins with the highest AUC values were further examined for their potential utility in stroke subtype classification.

## Results

### Patient characteristics

A total of 388 patients experiencing acute stroke were recruited within six hours from symptom onset. Table 1 summarizes the demographic and clinical characteristics of the study population. Of these, 344 patients (88.7%) were diagnosed with IS, while 44 patients (11.3%) were classified as having ICH. The overall cohort included a slightly higher proportion of males across both stroke subtypes. The median age at onset was similar for both IS and ICH, with a median of 77 years. Hypertension was the most prevalent comorbidity, showing a significantly higher frequency among patients with ICH compared to those with IS (*p* = 0.003). A prior history of stroke was also more common in the ICH group. Notably, among patients with a previous stroke, 90% of those in the IS group had experienced a prior ischemic event, whereas 50% of patients in the ICH group had a history of ICH. Regarding stroke severity, the median NIHSS score upon hospital admission showed no statistically significant difference between groups. However, at discharge, patients with ICH had significantly higher NIHSS scores compared to those with IS (*p* = 0.009).

**Table 1:** Presents demographic data of patients who suffered ischemic stroke or intracerebral hemorrhage

	IS	ICH	*P* value
*N* (%)	344 (88.7%)	44 (11.3%)	-
Gender, female (*n* [%])	161 (46.8%)	17 (38.6%)	0.306^1^
Age (median [IQR])	77 (16)	78 (11.5)	0.369^2^
Hypertension (*n* [%])	249 (72.4%)	41 (93.2%)	**0.003**^1^
Diabetes mellitus (*n* [%])	92 (26.7%)	14 (29.5%)	0.694^1^
Atrial fibrillation (*n* [%])	79 (23.0%)	5 (11.4%)	0.079^1^
Previous stroke (*n* [%])	35 (10.2%)	12 (27.3%)	**0.001**^1^
Antiplatelets (*n* [%])	106 (30.8%)	12 (27.3%)	0.831^1^
Anticoagulants (*n* [%])	60 (17.4%)	4 (9.1%)	0.345^1^
NIHSS admission (median [IQR])	12.5 (12.2)	15.5 (9.2)	0.468^2^
NIHSS discharge (median [IQR])	3 (9)	8.5 (9)	**0.009**^2^

*IS* ischemic stroke, *ICH* intracerebral hemorrhage, *IQR* interquartile range

^1^Chi-square test

^2^Mann-Whitney

### Differential protein expression between ischemic stroke and intracerebral hemorrhage

Out of the initial 3,072 proteins analyzed using Olink® multiplex technology, a total of 2,531 proteins met the inclusion criteria and were retained for differential expression analysis. Among these, 878 proteins showed nominal differential expression (*p* < 0.05), with 844 overexpressed in ICH and 34 in IS. Of these, 67 remained significant after multiple testing correction (adjusted *p* < 0.05).

In addition, proteins with intermediate missingness were defined as those with at least 30% of samples below LOD in at least one group and less than 70% missing across the full cohort. These proteins were assessed for group-specific ‘on/off’ detection using two-sided Fisher’s exact tests. A subset of proteins showed significant differences in detection between IS and ICH (*p* < 0.05) and are reported in Supplementary Table S1.

The top 12 proteins with the greatest differences in fold-change and statistical significance are summarized in Table 2. The most significantly upregulated proteins in ICH compared to IS included glial fibrillary acidic protein (GFAP), EH domain-containing protein 3 (EHD3), dual adapter for phosphotyrosine and 3-phosphoinositides 1 (DAPP1), serpin H1 (SERPINH1), low-density lipoprotein receptor adaptor protein 1 (LDLRAP1), and serine/threonine-protein kinase AKT2. These proteins demonstrated both high statistical significance and elevated expression in ICH. Conversely, the proteins most significantly overexpressed in IS included survival of motor neuron domain-containing protein 1 (SMNDC1), killer cell immunoglobulin-like receptor 3DL1 (KIR3DL1), N-terminal pro-B-type natriuretic peptide (NT-proBNP), protein-arginine deiminase type-4 (PADI4), S100 calcium-binding protein A12 (S100A12), isovaleryl-CoA dehydrogenase (IVD), and myeloid cell nuclear differentiation antigen (MNDA).

**Table 2:** Top 12 differentially expressed proteins between ischemic stroke (IS) and intracerebral hemorrhage (ICH). The table shows the six proteins most significantly upregulated in ICH and the six most significantly upregulated in IS, ranked according to statistical significance (*p*-value < 0.05) and absolute log2 fold change. Columns CI.L and CI.R indicate the lower and upper bounds of the 95% confidence interval for the log2 fold change, providing the range of compatible effect estimates. Adjusted *p*-values were calculated using the Holm–Bonferroni correction method

Assay	UniProt	log_2_FC	CI.L	CI.*R*	t	*P*.Value	adj.PVal
GFAP	P14136	−3.907459	−4.449042	−3.365875	−14.185025	< 0.0001	< 0.0001
EHD3	Q9NZN3	−2.053637	−2.752745	−1.354529	−5.775383	< 0.0001	< 0.0001
DAPP1	Q9UN19	−2.036054	−2.828035	−1.244073	−5.054473	< 0.0001	< 0.0001
SERPINH1	P50454	−1.928227	−2.752065	−1.104390	−4.601694	< 0.0001	< 0.0001
LDLRAP1	Q5SW96	−1.910247	−2.610797	−1.209697	−5.361070	< 0.0001	< 0.0001
AKT2	P31751	−1.874836	−2.686570	−1.063102	−4.540992	< 0.0001	< 0.0001
SMNDC1	O75940	0.701110	0.193148	1.209071	2.713667	0.007	1
NT-proBNP	NTproBNP	0.758516	0.031575	1.485457	2.051477	0.041	1
PADI4	Q9UM07	0.758990	0.231817	1.286163	2.830635	0.005	1
S100A12	P80511	0.840987	0.405042	1.276932	3.792788	< 0.001	0.408996
IVD	P26440	1.017959	0.373098	1.662819	3.103600	0.002	1
MNDA	P41218	1.053378	0.430868	1.675888	3.326895	< 0.0001	1

A comprehensive list of all differentially expressed proteins, including Log2 fold-change values, 95% confidence intervals, and *p*-values, is available in Supplementary Table S2.

**Fig. 1:**
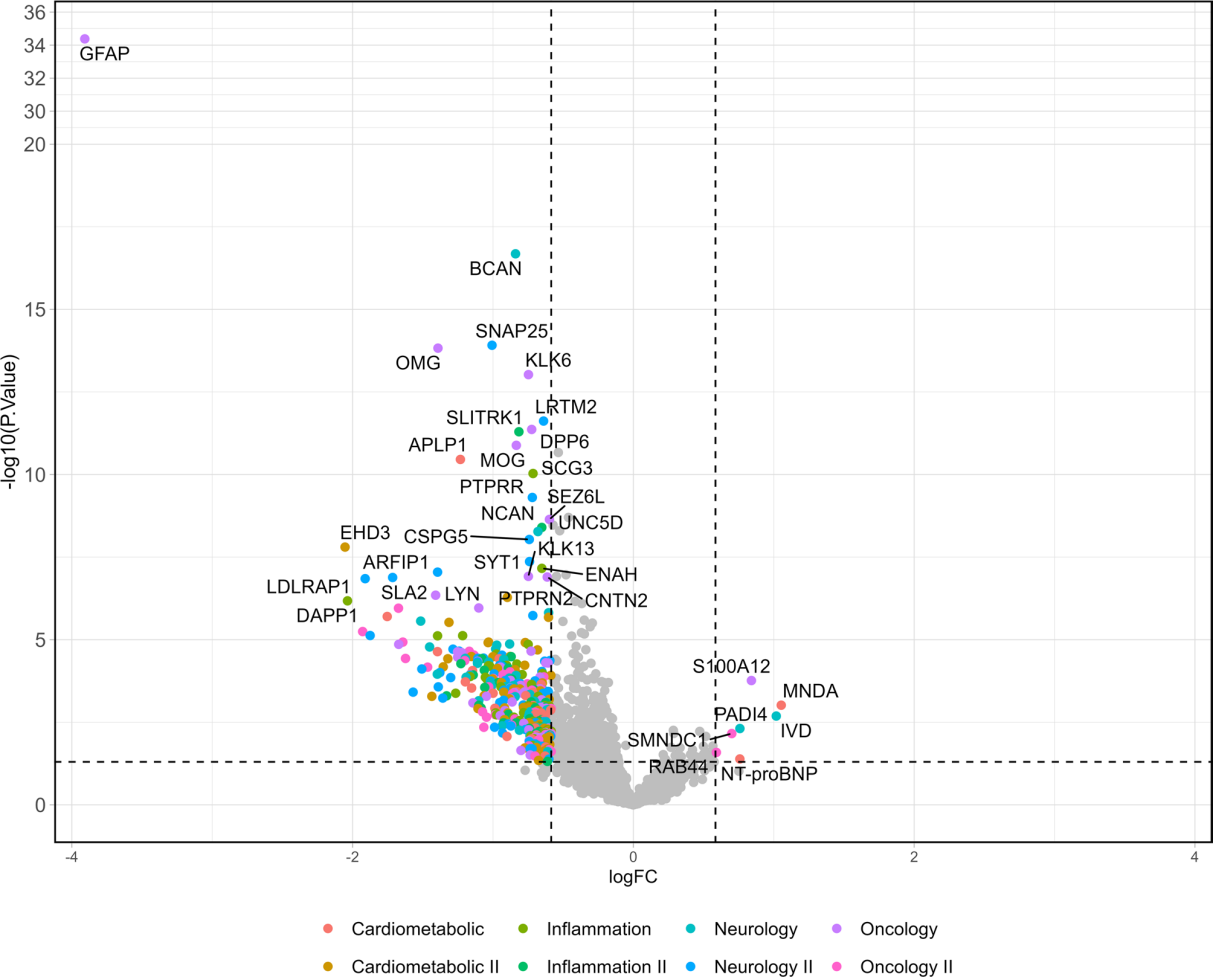
Volcano plot displaying the differentially expressed proteins between ischemic stroke (IS) and intracerebral hemorrhage (ICH). Each dot corresponds to one protein, positioned by its log2 fold change (x-axis) and –log10(*p*-value) (y-axis). Proteins upregulated in ICH appear on the left (negative logFC), while those upregulated in IS are on the right (positive logFC). Dashed vertical lines indicate the thresholds for logFC ± 0.6, and the horizontal dotted line corresponds to a *p*-value threshold of 0.05. Selected proteins with the most significant differential expression are labeled. Colors indicate the Olink^®^ panel classification of each protein

**Fig. 2:**
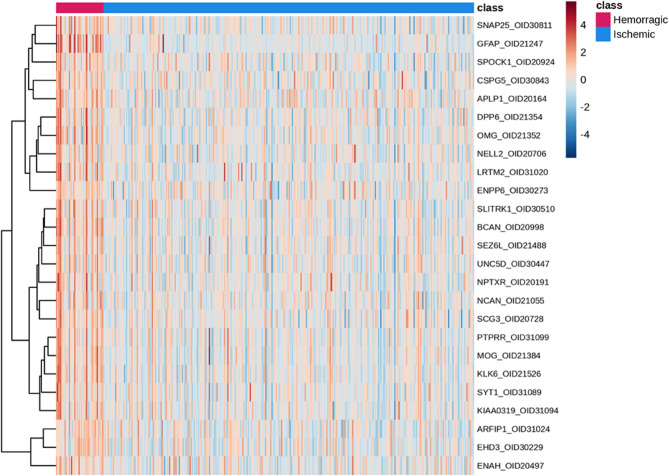
Heatmap of the top differentially expressed proteins between intracerebral hemorrhage (ICH) and ischemic stroke (IS). The heatmap displays z-score normalized expression levels of the most discriminative proteins across individual patient samples, grouped by stroke subtype (top annotation bar: red for ICH, blue for IS). Each row corresponds to a protein, and each column represents a patient. Color intensity reflects relative protein abundance, with warmer tones indicating higher expression and cooler tones indicating lower expression across the cohort

The volcano plot (Fig. 1) visually illustrates these differential patterns, with GFAP emerging as the most significantly overexpressed protein in ICH, displaying the highest –log10(*p*-value) and the largest positive log2 fold change. Additional proteins overexpressed in ICH, such as EHD3, DAPP1, SERPINH1, LDLRAP1, and AKT2, are also prominently represented. In contrast, IS-enriched proteins including S100A12, PADI4, IVD, MNDA, SMNDC1, NT-proBNP, and KIR3DL1 appear on the opposite side of the plot. To further explore expression profiles, we generated a heatmap based on the top differentially expressed proteins (Fig. 2). Proteins such as GFAP, SNAP25, and SPOCK1 were strongly upregulated in ICH.

**Fig. 3:**
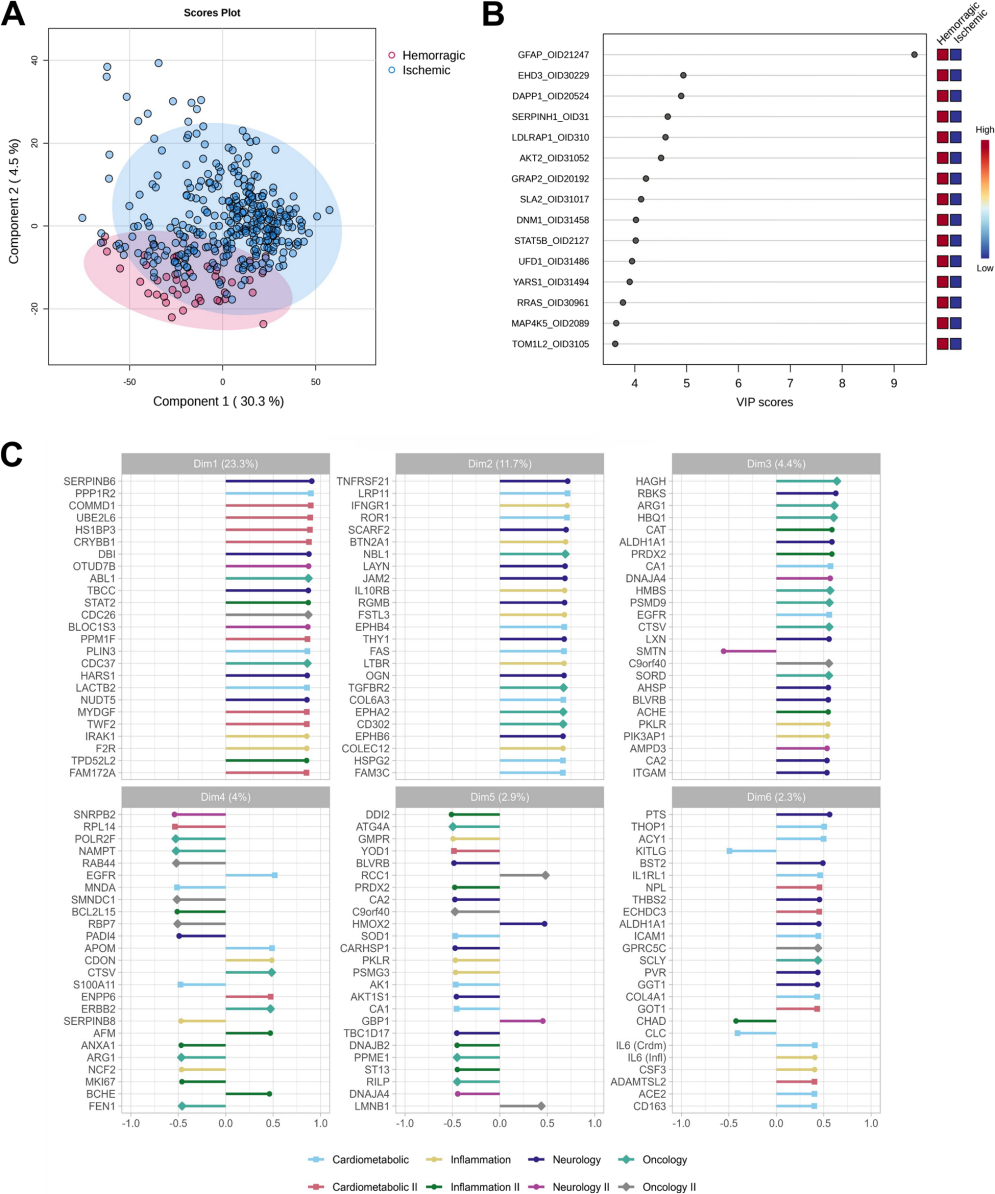
**A** Partial Least Squares Discriminant Analysis (PLS-DA) score plot comparing ischemic stroke and intracerebral hemorrhage patients. Each point represents an individual patient projected onto the first two PLS components based on normalized protein expression (NPX) values. Ischemic cases are shown as blue circles and hemorrhagic cases as red squares. Ellipses represent 95% confidence intervals for each group, illustrating the separation between stroke subtypes. **B** Bar plot of Variable Importance in Projection (VIP) scores for the top 15 proteins contributing to stroke subtype classification. Each dot represents a protein, ordered by decreasing VIP score. An adjacent heatmap shows protein expression across groups, with red indicating high and blue low expression levels. **C** Correlation of individual proteins with principal component dimensions based on NPX values. Bar plots display the correlation coefficients of proteins with the first six principal components (Dim1–Dim6), obtained through Principal Component Analysis (PCA) to explore structure and variation in the dataset. Each component’s explained variance is indicated in parentheses. Proteins are color-coded by Olink^®^ panel

To assess the discriminative capacity of the proteomic profiles, we performed a Partial Least Squares Discriminant Analysis (PLS-DA). The resulting score plot (Fig. 3A) revealed a clear separation between IS and ICH patients, supporting the presence of distinct protein expression patterns between the groups. Variable Importance in Projection (VIP) scores derived from the PLS-DA model (Fig. 3B) identified GFAP as the most influential contributor to group separation. Other proteins with high VIP scores included EHD3, DAPP1, SERPINH1, and LDLRAP1, all exceeding a VIP threshold of four. Additional contributors with elevated VIP scores included AKT2, GRAP2, SLA2, DNM1, and STAT5B.

In parallel, we performed Principal Component Analysis (PCA) on the NPX data to explore the underlying structure and identify protein contributions to each dimension (Fig. 3C). The first principal component (Dim1), accounting for 23.3% of total variance, was mainly influenced by proteins from the cardiometabolic panel, with SERPINB6, PPP1R2, COMMD1, and HS1BP3 showing the strongest correlations. The second dimension (Dim2), explaining 11.7% of the variance, was enriched for proteins involved in inflammation and immune regulation, including TNFRSF21, LRP1, IFNGR1, and FSTL3. Further components (Dim3 to Dim6), each accounting for between 4.4% and 2.3% of variance, captured additional biological processes, such as oxidative stress (CAT, SOD1), metabolic regulation (NAMPT, RPL14), and neuroinflammation (IL6, THOP1, GOT1).

### Functional enrichment analysis

Functional enrichment analysis was carried out to better understand the biological context of the differentially expressed proteins (DEPs). Gene ontology and tissue-specific enrichment approaches were applied to identify overrepresented biological categories.

**Fig. 4:**
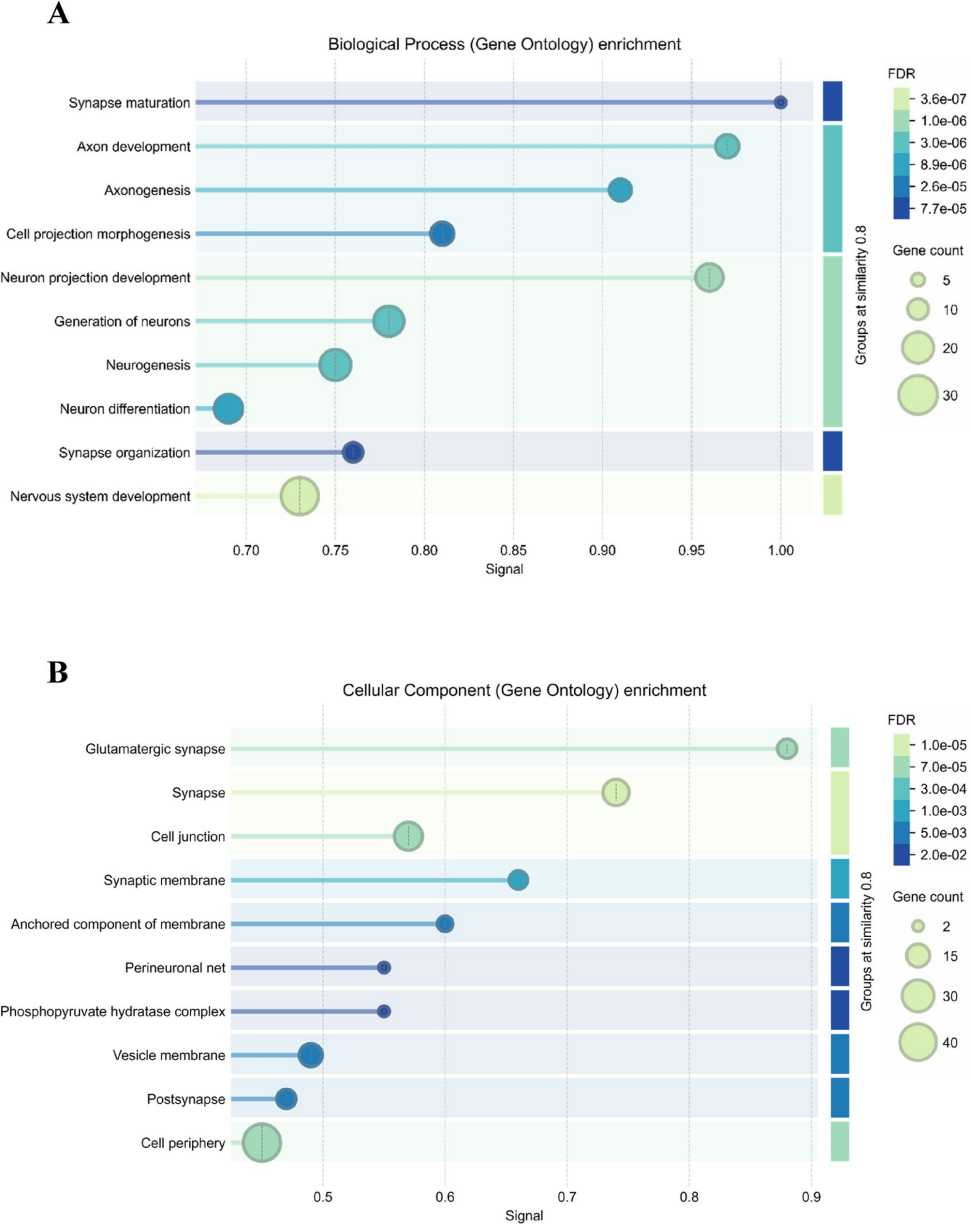
Functional enrichment of differentially expressed proteins by gene ontology. **A** Gene ontology (GO) enrichment for Biological process and (**B**) Cellular Component enrichment terms based on protein expression profiles. Each dot represents a significantly enriched term, grouped by functional similarity. Dot size reflects the number of associated proteins, while the x-axis indicates the enrichment signal score. Color intensity represents the false discovery rate (FDR), ranging from light green (lowest FDR) to dark blue (higher FDR)

In the Biological Process category (Fig. 4A), the most statistically enriched term was nervous system development (FDR = 3.6 × 10⁻^7^), which was also the process with the highest number of associated proteins. Other significantly enriched processes included neuron differentiation, neurogenesis, and generation of neurons, all reflecting strong associations with neurodevelopmental pathways. All these processes showed significant enrichment.

For the Cellular Component category (Fig. 4B), the most significantly enriched structure was the synapse, which had the lowest FDR among all cellular components. Other relevant terms included glutamatergic synapse and cell junction. Notably, the cell periphery encompassed the largest number of DEPs, pointing to widespread alterations in membrane-associated and intercellular structures.

In the Tissue Enrichment analysis (Supplementary Fig. 1), brain-related anatomical regions were prominently enriched. The brain showed the highest number of mapped proteins, followed by the temporal lobe and cerebral lobe. Notably, certain brain areas exhibited the lowest FDR values, highlighting strong enrichment regardless of differences in protein counts across regions.

### Protein-protein interaction network analysis

**Fig. 5:**
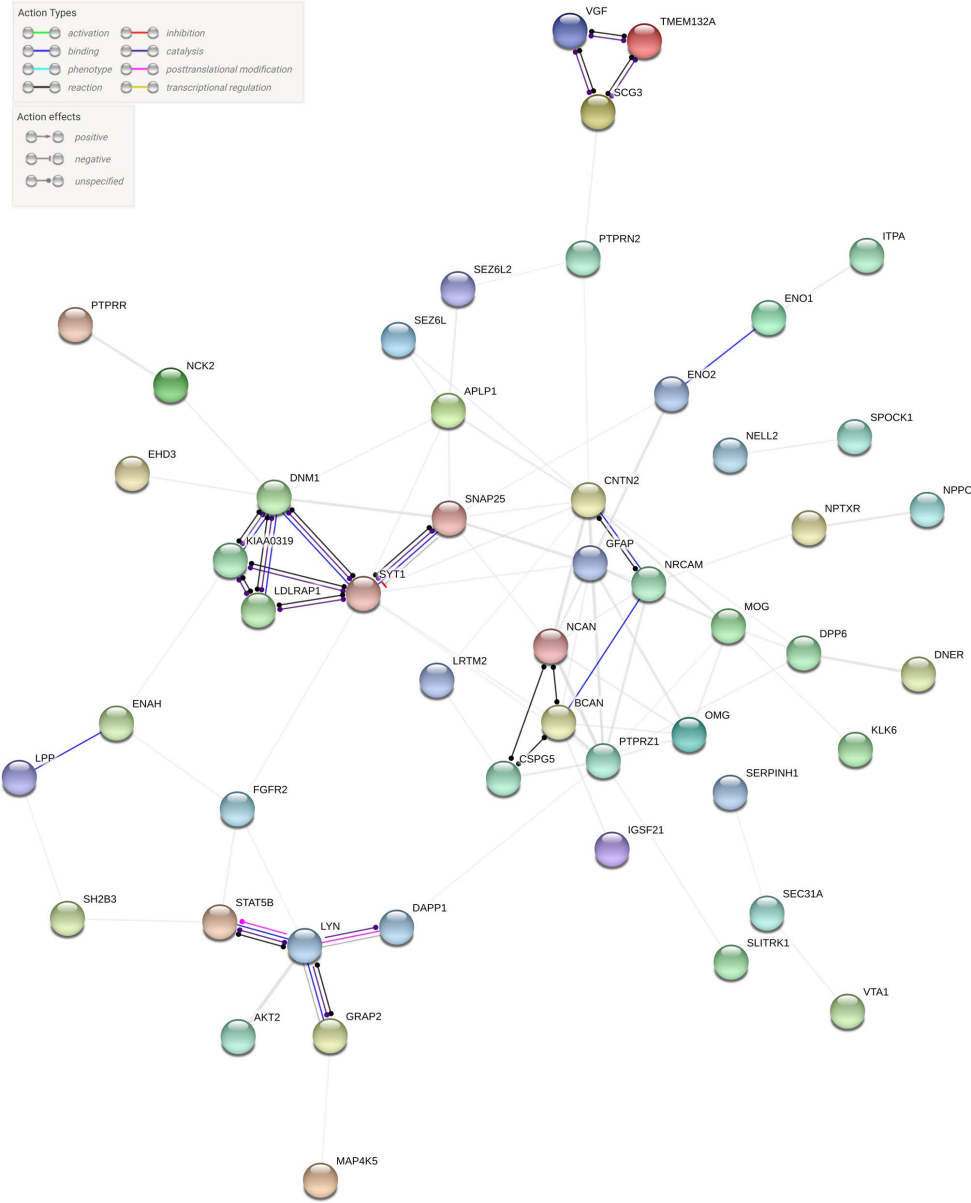
Protein–protein interaction (PPI) network constructed from significantly differentially expressed proteins using the STRING database. Nodes represent proteins; edges represent known or predicted interactions. Line thickness reflects interaction confidence. Proteins are colored based on functional clusters or annotation categories

A PPI network was generated using the STRING database to examine the connectivity and potential functional relationships among the most significantly DEPs, filtered by FDR correction. The resulting network revealed a highly organized and modular structure, with distinct clusters reflecting biological processes central to stroke pathophysiology (Fig. 5).

A prominent and densely interconnected core was identified, comprising glial and synaptic proteins such as GFAP, CNTN2, SNAP25, NCAN, and BCAN. This cluster formed a compact module associated with neuroglial signaling and extracellular matrix interactions. GFAP was positioned centrally within this module, showing extensive connectivity with proteins including MOG, OMG, CSPG5, and PTPRZ1, consistent with its central role in glial function and structural regulation.

A second distinct module was centered around LYN, which was connected to STAT5B, GRAP2, AKT2, and DAPP1. These proteins formed a cohesive cluster with multiple interactions, suggesting a coordinated network involved in signaling pathways related to inflammation and immune activation.

Additional clusters were observed involving proteins such as LDLRAP1, DNM1, and SYT1, forming a satellite group associated with synaptic vesicle trafficking and neuronal communication. Proteins including EHD3, SCG3, and APLP1 displayed intermediate connectivity, bridging the glial-synaptic and immune-related modules. More peripheral proteins such as ITPA, SEZ6L, and MAP4K5 showed fewer connections, consistent with more isolated or context-specific functional roles.

### Diagnostic performance of discriminatory proteins

**Fig. 6:**
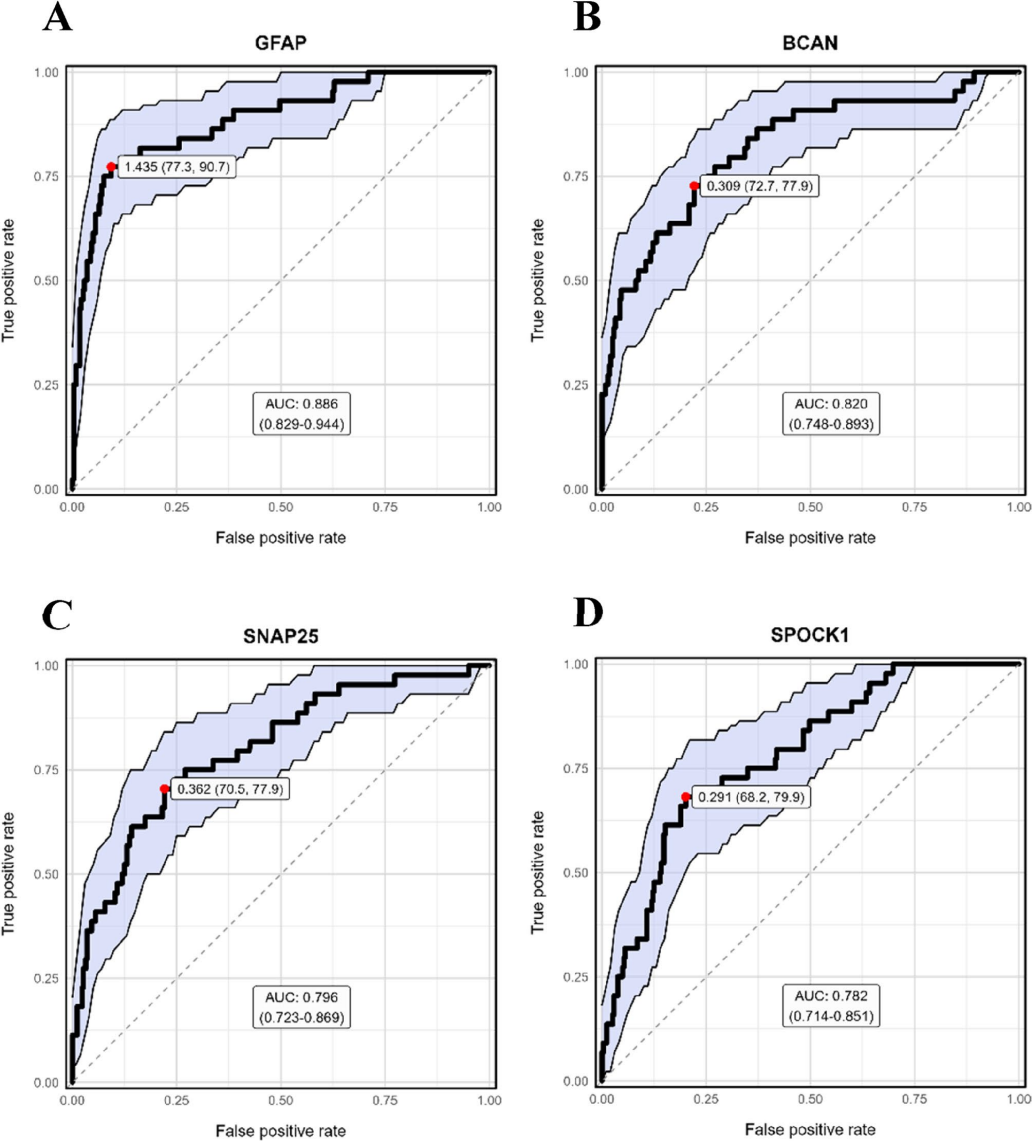
Receiver Operating Characteristic (ROC) curve analysis for the four proteins with the highest discriminatory power between intracerebral hemorrhage and ischemic stroke. ROC plots show AUC values with 95% confidence intervals, with the optimal cutoff points indicated by the red dot. **A** GFAP, **B** BCAN, **C** SNAP25, **D** SPOCK1

To evaluate the ability of individual proteins to distinguish between IS and ICH, ROC curve analyses were conducted for all significantly differentially expressed proteins. Among them, four proteins, GFAP, BCAN, SNAP25, and SPOCK1, showed the highest classification performance (Table 3). GFAP achieved the best discriminatory capacity, with an AUC of 0.886 (95% CI: 0.829–0.944), a sensitivity of 77.3%, and a specificity of 90.7% at an optimal threshold of 1.435 (Fig. 6A). BCAN followed with an AUC of 0.82 (95% CI: 0.748–0.893), achieving balanced sensitivity (72.7%) and specificity (77.9%) at a threshold of 0.309 (Fig. 6B).

**Table 3:** Diagnostic performance of intracerebral hemorrhage-enriched biomarkers. Diagnostic accuracy metrics for proteins upregulated in intracerebral hemorrhage

Biomarker	Cut-off	AUC (95% CI)	Sensitivity (95% CI)	Specificity (95% CI)	PPV (95% CI)	NPV (95% CI)
GFAP	1.435	0.886 (0.829–0.944)	77.3 (63.0–87.2)	90.7 (87.2–93.3)	51.5 (39.7–63.2)	96.9 (94.4–98.3)
BCAN	0.309	0.82 (0.748–0.893)	72.7 (58.2–83.7)	77.9 (73.2–82.0)	29.6 (21.8–38.8)	95.7 (92.7–97.5)
SNAP25	0.362	0.796 (0.723–0.869)	70.5 (55.8–81.8)	77.9 (73.2–82.0)	29.0 (21.2–38.2)	95.4 (92.2–97.3)
SPOCK1	0.291	0.782 (0.714–0.851)	68.2 (53.4–80.0)	79.9 (75.4–83.8)	30.3 (22.1–40.0)	95.2 (92.0–97.1)

*CI* confidence interval, *PPV* positive predictive value, *NPV* negative predictive value, *AUC* area under the ROC curve

SNAP25 and SPOCK1 also demonstrated strong performance, with AUCs of 0.796 (95% CI: 0.723–0.869) and 0.782 (95% CI: 0.714–0.851), respectively. The optimal threshold for both proteins was close to 0.30, and their expression levels were moderately higher in ICH (Fig. 6C and D).

In addition to the top-performing proteins associated with ICH, ROC analyses were also conducted for proteins upregulated in IS. Their performance is shown in Supplementary Figure S2 and detailed in **S**upplementary Table S3. Among them, S100A12 exhibited the highest classification performance, with an AUC of 0.675 (95% CI: 0.598–0.753), followed by MNDA (AUC 0.657, 95% CI: 0.567–0.740). PADI4, SMNDC1, NT-proBNP, and IVD showed lower, yet statistically significant, discriminatory capacities, with AUCs ranging from 0.602 to 0.635. Although the discriminatory power of these proteins was modest compared to those enriched in ICH, their expression profiles demonstrated clear trends of upregulation in IS patients, as shown in the violin plots in Supplementary Figure S3 and Supplementary S4 for ICH.

## Discussion

Accurate differentiation between IS and ICH is essential in the acute management of stroke patients, as treatment strategies differ fundamentally between the two conditions [5]. The present study provides a comprehensive proteomic characterization of IS and ICH within the first six hours after symptom onset, utilizing an extensive multiplex analysis of 3,072 proteins. Our findings highlight profound molecular differences between the two stroke subtypes, offering important insights into their distinct biological underpinnings. Notably, GFAP emerged as the most discriminative biomarker, achieving an excellent diagnostic performance for differentiating between IS and ICH, a distinction that holds immediate clinical relevance for optimizing acute therapeutic strategies. Notably, in the case of ICH, early identification in the prehospital setting could enable rapid initiation of blood pressure-lowering strategies. This is particularly important given the findings of the INTERACT-4 trial [20], which demonstrated that ultra-early blood pressure reduction in ICH patients significantly improves functional outcomes and reduces mortality. Moreover, early differentiation could inform triage decisions, such as prioritizing transfer to the nearest hospital for urgent management, rather than delaying care by routing patients to more distant high-complexity centers, which may negatively impact outcomes in ICH [21].

The demographic and clinical features of our cohort align with known stroke epidemiology. Hypertension was the most prevalent comorbidity and significantly more common in ICH than IS (*p* = 0.003), consistent with the INTERSTROKE study, which identified hypertension as the strongest modifiable risk factor for stroke, particularly for hemorrhagic events [22]. Although initial NIHSS scores were similar, ICH patients showed greater neurological deterioration by discharge (*p* = 0.009), in line with evidence linking hematoma expansion and perihematomal edema to worse outcomes [23, 24]. A history of stroke was also more frequent in ICH patients, echoing data showing a 13.1% five-year recurrence after ICH, mainly driven by hypertension [25]. These findings highlight the interplay between vascular risk factors, stroke subtype, and clinical severity in shaping early complications and reinforce the more severe prognosis of ICH compared to IS.

Proteomic analysis revealed a marked predominance of proteins overexpressed in ICH (844 vs. 34 in IS), highlighting the profound molecular disruption associated with ICH. GFAP emerged as the most discriminative biomarker, consistent with prior studies. Luger et al. reported significantly elevated serum GFAP in ICH compared to IS within 6 h of onset (AUC 0.872; sensitivity 77.8%, specificity 94.2%) [26]. Similarly, a meta-analysis by Cabezas et al. confirmed higher GFAP levels in ICH and their correlation with stroke severity [27]. Furthermore, recent Simoa^®^-based studies demonstrated that GFAP can accurately distinguish stroke subtypes in the prehospital phase, achieving 96.6% sensitivity and 98.4% negative predictive value within 3 h [12]. In our study, GFAP showed the highest VIP score, best individual AUC (0.889), and a central role in the PPI network, reinforcing its diagnostic and biological significance in early ICH. These findings are also consistent with the recent study by Misra et al. [28], who applied a different proteomic approach (SWATH-MS in discovery and targeted proteomics in validation) and identified GFAP, together with MMP-9 and APO-C1, as independent biomarkers capable of differentiating IS from ICH with high accuracy (92%). Notably, GFAP emerged as the strongest biomarker in both studies, underscoring its reproducibility across independent cohorts and analytical platforms. While Misra et al. focused on samples obtained within 24 h, our study specifically concentrated on the hyperacute window (≤ 6 h) and employed a broader high-throughput proteomic strategy that additionally uncovered novel discriminatory candidates such as BCAN, SNAP25, and SPOCK1. Together, these complementary results reinforce the robustness of GFAP and illustrate the added value of large-scale profiling for expanding the biomarker landscape.

Beyond GFAP, several proteins, including EHD3, DAPP1, SERPINH1, and LDLRAP1, emerged with high VIP scores and marked overexpression in ICH, highlighting their potential involvement in the pathophysiological response to ICH. EHD3, involved in early endosome-to-recycling compartment transport and Golgi organization, has been linked to vesicular trafficking and lysosomal biosynthesis, processes essential for synaptic integrity and neuronal repair following injury. Disruption of these pathways has been associated with impaired neuronal recovery, suggesting that EHD3 upregulation may reflect a compensatory mechanism activated in response to hemorrhagic stress [29, 30]. DAPP1, a PI3K-pathway adapter and known regulator of GPVI-mediated platelet signaling, may represent a feedback mechanism aiming to modulate platelet reactivity and limit secondary thrombotic complications in the setting of vascular rupture [31]. SERPINH1 encodes HSP47, a collagen-specific molecular chaperone that contributes to extracellular matrix stabilization and has been associated with protective responses in hypoxic-ischemic encephalopathy [32]. Its expression may be driven by ER stress and tissue remodeling following blood–brain barrier (BBB) disruption. LDLRAP1, a cytosolic adaptor for LRP1, may reflect increased endocytic clearance of heme and lipid debris, processes critical for limiting oxidative damage and preserving neurovascular integrity after erythrocyte lysis [33]. Although these proteins have not been previously characterized as stroke biomarkers, their biological functions, ranging from vesicular trafficking and hemostasis regulation to extracellular matrix remodeling and neurovascular protection, align closely with processes expected to differ between ICH and IS. Their consistent elevation in ICH relative to IS and contribution to multivariate discrimination support their potential as biomarkers and warrant further exploration in the context of acute ICH.

In addition to GFAP, several other proteins demonstrated high discriminatory capacity for ICH. BCAN, a key component of the perisynaptic extracellular matrix, showed strong performance (AUC = 0.820) and is known to modulate synaptic plasticity through its localization around excitatory synapses. Its upregulation in experimental models of synaptic remodeling suggests a dynamic response to acute neuronal disruption [34], while lower levels observed in chronic cerebrovascular disease, such as vascular dementia, may reflect sustained white matter injury and long-term extracellular matrix (ECM) depletion [35]. SNAP25 also showed robust classification performance for ICH (AUC = 0.797). Although often elevated in IS and neurodegenerative diseases like Alzheimer’s and Creutzfeldt-Jakob disease [36–38], its increased levels in our ICH cohort may be explained by the acute synaptic injury and BBB breakdown that characterize hemorrhagic events. Consistent with this, SNAP25 has also been reported to rise in traumatic brain injury (TBI), where it correlates with inflammation and unfavorable outcomes [39]. SPOCK1, another high-performing marker (AUC = 0.786), has been associated with reactive astrocytosis and neurovascular remodeling. Animal studies show its upregulation in astrocytes near brain lesions, where it co-localizes with FGF-2, suggesting a role in axonal repair [40], and it has also been shown to regulate BBB permeability through modulation of endothelial–pericyte interactions [41]. Altogether, the discriminatory power of BCAN, SNAP25, and SPOCK1, combined with their biological relevance to synaptic damage, astroglial reactivity, and ECM remodeling, highlights their potential utility, alongside GFAP, as a multi-marker panel for distinguishing ICH from IS.

Several proteins were significantly overexpressed in IS, though with more modest discriminative performance compared to ICH-associated markers. S100A12, a calcium-binding proinflammatory protein, stood out among the top IS markers. Elevated plasma levels have been linked to increased stroke severity, poorer 90-day outcomes, and genetic predisposition to larger infarct volumes [42–44]. Experimental models support its involvement in promoting inflammation and apoptosis via ERK signaling [45]. However, its elevation in other acute brain injuries, such as ICH and TBI [46–48], suggests it reflects general neuroinflammatory activity rather than IS-specific processes, aligning with its moderate ROC performance. MNDA is a nuclear protein expressed in leukocytes and part of the PYHIN family, involved in regulating inflammation and apoptosis. It modulates gene transcription in response to inflammatory stimuli or cellular stress [49]. Although not previously linked to stroke, its upregulation in IS may reflect early monocyte and neutrophil activation, suggesting a role as a marker of sterile inflammation. PADI4 appears to have a dual role in the ischemic brain. It may promote functional recovery by activating neurorepair genes via histone citrullination, mediated by lipids like 15-HETrE [50]. Simultaneously, it contributes to neutrophil extracellular trap formation, driving inflammation and secondary injury [51, 52]. Its elevation may thus reflect both detrimental inflammatory responses and intrinsic neuronal repair mechanisms. NT-proBNP, significantly elevated in IS in our study, is a recognized marker of cardiac dysfunction and atrial fibrillation, especially relevant in cardioembolic stroke. Its elevation has been consistently linked to increased stroke risk, mortality, and poor outcomes [13]. It also shows high specificity (0.93) for distinguishing cardioembolic from non-cardioembolic stroke [53], and predicts stroke risk even in asymptomatic individuals [54]. Moreover, its use in point-of-care testing has shown promising accuracy for rapid diagnosis in prehospital settings [55]. Lastly, SMNDC1 and IVD, although less characterized in stroke, were among the top IS-overexpressed proteins; their roles in RNA processing and mitochondrial metabolism suggest responses to ischemia-induced stress and energy deficits. Collectively, these findings highlight a proteomic profile in IS, relative to ICH, characterized by inflammatory signaling, immune cell activation, and metabolic adaptation, with emerging evidence suggesting possible concurrent activation of intrinsic repair mechanisms. This multifaceted pattern underscores the complexity of the ischemic cascade and supports the potential of these proteins as comparative markers and candidates for future biomarker panels.

The functional enrichment analysis of DEPs revealed significant overrepresentation of biological processes related to neuronal development and synaptic organization, with terms such as “neuron projection development” and “glutamatergic synapse” emerging as highly enriched. These findings were corroborated by PPI network analysis using the STRING database, which identified two major functional clusters centered around GFAP and LYN. The GFAP-associated module included proteins such as MOG, OMG, CNTN2, NRCAM, PTPRZ1, and SNAP25, reflecting coordinated glial, neuronal, and ECM-related differences between ICH and IS [39, 56–60]. GFAP’s centrality aligns with its established roles in astrogliosis, neuroinflammation, and BBB disruption after stroke [61, 62]. Notably, many of these same proteins were also found to be differentially expressed in a recent proteomic study of TBI, where they were linked to neuroregeneration and central nervous system (CNS) repair pathways [63]. These overlaps suggest that proteins more abundant in ICH relative to IS in our dataset are also involved in injury-response mechanisms previously described in brain trauma, indicating shared molecular programs across acute CNS injuries. Moreover, a large-scale imaging-proteomics study reported strong associations between GFAP, OMG, NCAN, and white matter microstructural alterations, reinforcing the brain specificity of these plasma biomarkers and supporting their relevance as indicators of acute CNS injury [64]. The second cluster, centered around LYN, included STAT5B, GRAP2, AKT2, and DAPP1, all overexpressed in ICH and with high VIP scores, pointing toward roles in immune activation and vascular dysfunction [65–68]. LYN has been associated with endothelial permeability and leukocyte recruitment, supporting its relevance in ICH pathophysiology [69, 70]. ECM-related proteins such as SPOCK1 and CSPG5 were also integrated within the network, consistent with their potential involvement in matrix remodeling during acute brain injury [41, 71]. Together, these interconnected modules suggest that glial, immune, and ECM-driven pathways are more prominent in ICH compared to IS.

When comparing our findings with previous proteomic studies in stroke and trauma, several overlaps and meaningful differences emerge that reinforce the biological relevance of our results. Many of the proteins elevated in ICH in our study, such as GFAP, SNAP25, SPOCK1, and BCAN, were also upregulated in TBI cohorts, where they were associated with axonal damage, glial activation, and synaptic disruption [63]. This convergence suggests that relative to IS, ICH and TBI may share overlapping patterns of injury-response pathways, supporting further exploration of cross-condition therapeutic strategies targeting glial and ECM remodeling. Similarly, proteins like S100A12, PADI4, MNDA, and NT-proBNP, found overexpressed in IS in our cohort, reflect systemic immune activation, neutrophil involvement, and cardioembolic mechanisms, consistent with other IS proteomic studies [72–75]. Although some proteins highly discriminatory in other studies (e.g., F2, F9, PLG, C1S, and PROS1 in the SWATH/MRM study [73]; THBS1, LYVE1, and IGF2 in the LVO study [76]) were not detected in our panel, they are involved in similar coagulation, vascular integrity, and extracellular signaling pathways as PADI4 and NT-proBNP in our data. Likewise, while our analysis identified SPOCK1 and BCAN as ECM-related markers, other studies found ECM proteins like fibulin-1, fibronectin, and MMP9 elevated in stroke [72, 77, 78], highlighting consistent engagement of tissue remodeling pathways across stroke types. Broadly, IS profiles across studies point to downregulation of synaptic proteins and upregulation of inflammatory, metabolic, and coagulation-related proteins, while ICH shows a stronger enrichment of extracellular matrix remodeling and coagulation activity [78]. Although specific protein hits may vary due to methodological and cohort differences (e.g., sample type, timing, or analytical platform), the convergence of affected pathways, including neuroinflammation, complement/coagulation cascades, and vascular stress, underscores the robustness of these molecular patterns and supports the potential of shared and subtype-specific biomarkers for improving stroke diagnosis and stratification.

This study presents several important methodological strengths that enhance the robustness and potential translational value of its findings. Notably, it was conducted as a multicenter study, increasing the diversity and representativeness of the sample population and strengthening the external validity of the results. Furthermore, to our knowledge, this is the first proteomic investigation specifically designed to compare the molecular signatures of IS and ICH using high-throughput plasma-based analysis. The application of PEA technology allowed for the sensitive and specific quantification of a large number of proteins, including low-abundance biomarkers, making it particularly suitable for early stroke detection research. The protocolized collection of blood samples within six hours of symptom onset minimized preanalytical variability related to time-dependent protein expression, increasing the clinical relevance of the data for acute diagnosis. Despite these strengths, the study also has several limitations. The relatively small number of ICH cases compared to IS may have limited statistical power for certain analyses and increased the risk of type II errors. Additionally, the absence of an independent external validation cohort restricts the generalizability of the findings, underscoring the need for replication in larger, prospective studies. Another limitation is the lack of orthogonal validation techniques, such as ELISA or western blotting, to confirm the differential expression of the most relevant protein candidates. Moreover, the potential impact of the exact timing of blood sample collection within the 6-hour window was not evaluated, which could have introduced variability in protein levels. Future studies should consider statistical adjustment for sampling time or perform sensitivity analyses stratified by specific time intervals. Finally, while plasma is a practical and clinically accessible sample type, it may not fully reflect brain-specific molecular events, as peripheral immune responses and systemic processes can obscure signals originating from the central nervous system. In conclusion, this study identifies distinct proteomic profiles that clearly differentiate IS from ICH in the hyperacute phase. GFAP stands out as a highly promising early biomarker, but several other proteins and pathways also emerged, offering novel insights into the relative molecular differences between stroke subtypes. These findings refine our understanding of IS and ICH and provide a foundation for developing biomarker-based diagnostic tools. Importantly, the potential application of such markers in prehospital settings could facilitate early stroke subtype classification. This would enable timely implementation of targeted interventions—particularly critical in ICH, where rapid therapeutic decisions, such as blood pressure management, may significantly influence clinical outcomes. However, further validation in larger, independent cohorts is needed. Future research should integrate proteomic data with clinical and imaging parameters to build comprehensive diagnostic models that improve early classification and guide personalized treatment in acute stroke. 

## Supplementary Information

Supplementary Material 1.

Supplementary Material 2.

## Data Availability

The data underlying this article are available in the article.
