# Supplementary material for: Plasma proteomics uncovers divergent molecular signatures in ischemic stroke and intracerebral hemorrhage
Source: Biomark Res. 2025 Oct 28;13:136. doi: 10.1186/s40364-025-00848-1 (PMC12570678; doi:10.1186/s40364-025-00848-1)
Supplement: Supplementary file 1 — Supplementary Material 1. [file 40364_2025_848_MOESM1_ESM.docx]

**Table S1. Proteins with group-specific detection patterns identified by Fisher’s exact test.** Proteins with intermediate missingness (30–70%) showing significant “on/off” differences between ischemic stroke (IS) and intracerebral hemorrhage (ICH) are listed with their UniProt IDs and Fisher’s exact test p-values.

| **Assay** | **UniProt** | **Fisher Exact Test p-value** |
| --- | --- | --- |
| CSNK2A1 | P68400 | 1.75721E-05 |
| VASH1 | Q7L8A9 | 1.946E-05 |
| NMT1 | P30419 | 0.000196822 |
| SNX18 | Q96RF0 | 0.000196822 |
| NARS1 | O43776 | 0.000232876 |
| CSNK1D | P48730 | 0.000236848 |
| TST | Q16762 | 0.002276401 |
| AP2B1 | P63010 | 0.002872758 |
| SESTD1 | Q86VW0 | 0.003365296 |
| RUVBL1 | Q9Y265 | 0.007184351 |
| NMI | Q13287 | 0.010183433 |
| SH3BGRL2 | Q9UJC5 | 0.011552206 |
| SYTL4 | Q96C24 | 0.014033378 |
| ZNF75D | P51815 | 0.014511551 |
| FKBP1B | P68106 | 0.015299965 |
| CASC3 | O15234 | 0.015431826 |
| PER3 | P56645 | 0.015437512 |
| MRPL58 | Q14197 | 0.022257047 |
| FHIP2A | Q5W0V3 | 0.025395361 |
| DCTN6 | O00399 | 0.029141574 |
| EIF1AX | P47813 | 0.036232616 |
| KRT17 | Q04695 | 0.036232616 |
| MMP15 | P51511 | 0.036929472 |
| PFDN6 | O15212 | 0.037407221 |
| RTKN2 | Q8IZC4 | 0.037712966 |
| POLR2A | P24928 | 0.039359336 |
| CTF1 | Q16619 | 0.045926044 |
| NT5C3A | Q9H0P0 | 0.045926044 |
| MDH1 | P40925 | 0.0493298 |

**Table S2. Comprehensive list of the 878 proteins with nominal differential expression between ischemic stroke (IS) and intracerebral hemorrhage (ICH) (unadjusted p < 0.05)**. CI.L and CI.R denote the 95% confidence interval (CI) for log2 fold change. Adjusted p-values were computed using the Benjamini–Hochberg method (FDR)

| **Assay** | **UniProt** | **log_2_FC** | **CI.L** | **CI.R** | **t** | **P.Value** | **adj.P.Val** |
| --- | --- | --- | --- | --- | --- | --- | --- |
| GFAP | P14136 | -3.907459 | -4.449042 | -3.365875 | -14.185025 | 0.000000 | 0.000000 |
| EHD3 | Q9NZN3 | -2.053637 | -2.752745 | -1.354529 | -5.775383 | 0.000000 | 0.000039 |
| DAPP1 | Q9UN19 | -2.036054 | -2.828035 | -1.244073 | -5.054473 | 0.000001 | 0.001663 |
| SERPINH1 | P50454 | -1.928227 | -2.752065 | -1.104390 | -4.601694 | 0.000006 | 0.014110 |
| LDLRAP1 | Q5SW96 | -1.910247 | -2.610797 | -1.209697 | -5.361070 | 0.000000 | 0.000356 |
| AKT2 | P31751 | -1.874836 | -2.686570 | -1.063102 | -4.540992 | 0.000007 | 0.018552 |
| GRAP2 | O75791 | -1.753233 | -2.467214 | -1.039252 | -4.827853 | 0.000002 | 0.004951 |
| SLA2 | Q9H6Q3 | -1.714807 | -2.341795 | -1.087818 | -5.377209 | 0.000000 | 0.000327 |
| DNM1 | Q05193 | -1.673075 | -2.337767 | -1.008384 | -4.948764 | 0.000001 | 0.002780 |
| STAT5B | P51692 | -1.671306 | -2.417649 | -0.924964 | -4.402699 | 0.000014 | 0.034193 |
| UFD1 | Q92890 | -1.641900 | -2.369227 | -0.914574 | -4.438317 | 0.000012 | 0.029274 |
| YARS1 | P54577 | -1.622823 | -2.387228 | -0.858418 | -4.173964 | 0.000037 | 0.090297 |
| RRAS | P10301 | -1.568873 | -2.430555 | -0.707192 | -3.579663 | 0.000388 | 0.898310 |
| MAP4K5 | Q9Y4K4 | -1.514690 | -2.140329 | -0.889052 | -4.759939 | 0.000003 | 0.006809 |
| TOM1L2 | Q6ZVM7 | -1.506809 | -2.248244 | -0.765374 | -3.995638 | 0.000077 | 0.185996 |
| SMAD2 | Q15796 | -1.465539 | -2.180966 | -0.750112 | -4.027478 | 0.000068 | 0.163605 |
| PMVK | Q15126 | -1.451370 | -2.105670 | -0.797070 | -4.361162 | 0.000017 | 0.040939 |
| POMC | P01189 | -1.435436 | -2.241612 | -0.629261 | -3.500707 | 0.000518 | 1.000000 |
| SH2B3 | Q9UQQ2 | -1.408357 | -1.947710 | -0.869004 | -5.133822 | 0.000000 | 0.001124 |
| PRTFDC1 | Q9NRG1 | -1.396438 | -2.099404 | -0.693473 | -3.905611 | 0.000111 | 0.265689 |
| ITGB1BP2 | Q9UKP3 | -1.395168 | -2.035225 | -0.755111 | -4.285576 | 0.000023 | 0.056589 |
| IRAK4 | Q9NWZ3 | -1.394511 | -1.998899 | -0.790123 | -4.536361 | 0.000008 | 0.018927 |
| ARFIP1 | P53367 | -1.394417 | -1.897531 | -0.891302 | -5.449124 | 0.000000 | 0.000226 |
| OMG | P23515 | -1.391737 | -1.733971 | -1.049502 | -7.995304 | 0.000000 | 0.000000 |
| NAA80 | Q93015 | -1.389525 | -2.132308 | -0.646742 | -3.677946 | 0.000268 | 0.628110 |
| DOK1 | Q99704 | -1.378064 | -2.066780 | -0.689348 | -3.933965 | 0.000099 | 0.237758 |
| GSTT2B | P0CG30 | -1.357363 | -2.127216 | -0.587509 | -3.466482 | 0.000586 | 1.000000 |
| PMM2 | O15305 | -1.354044 | -2.014206 | -0.693882 | -4.032585 | 0.000066 | 0.160362 |
| SNCA | P37840 | -1.330277 | -2.075433 | -0.585122 | -3.509912 | 0.000501 | 1.000000 |
| EIF2S2 | P20042 | -1.321549 | -1.944765 | -0.698333 | -4.169134 | 0.000038 | 0.092035 |
| ITPA | Q9BY32 | -1.313123 | -1.857677 | -0.768568 | -4.740949 | 0.000003 | 0.007434 |
| PHACTR2 | O75167 | -1.301143 | -1.966343 | -0.635943 | -3.845687 | 0.000140 | 0.334610 |
| OPHN1 | O60890 | -1.284743 | -1.868702 | -0.700784 | -4.325485 | 0.000019 | 0.047760 |
| MANF | P55145 | -1.265565 | -1.964633 | -0.566496 | -3.559311 | 0.000418 | 0.966679 |
| CDKN1A | P38936 | -1.252195 | -1.827038 | -0.677351 | -4.282754 | 0.000023 | 0.057256 |
| DNAJB1 | P25685 | -1.251100 | -1.837300 | -0.664899 | -4.196109 | 0.000034 | 0.082424 |
| MINK1 | Q8N4C8 | -1.240285 | -1.808280 | -0.672290 | -4.293172 | 0.000022 | 0.054816 |
| APLP1 | P51693 | -1.231856 | -1.586975 | -0.876736 | -6.820037 | 0.000000 | 0.000000 |
| DTD1 | Q8TEA8 | -1.228440 | -1.819379 | -0.637501 | -4.087073 | 0.000053 | 0.128734 |
| DAB2 | P98082 | -1.222960 | -1.786044 | -0.659876 | -4.270119 | 0.000025 | 0.060419 |
| NCK2 | O43639 | -1.215995 | -1.742719 | -0.689272 | -4.538896 | 0.000008 | 0.018720 |
| GIPC3 | Q8TF64 | -1.200819 | -1.771364 | -0.630275 | -4.137987 | 0.000043 | 0.104679 |
| TBCB | Q99426 | -1.197044 | -1.760712 | -0.633377 | -4.175309 | 0.000037 | 0.089825 |
| DIABLO | Q9NR28 | -1.196927 | -1.821240 | -0.572614 | -3.769348 | 0.000189 | 0.446740 |
| MESD | Q14696 | -1.191335 | -1.799579 | -0.583091 | -3.850858 | 0.000138 | 0.328153 |
| MTSS2 | Q765P7 | -1.168850 | -1.704385 | -0.633314 | -4.291132 | 0.000022 | 0.055277 |
| RHOC | P08134 | -1.164863 | -1.753924 | -0.575803 | -3.887912 | 0.000119 | 0.284322 |
| MGMT | P16455 | -1.162789 | -1.754307 | -0.571271 | -3.864863 | 0.000130 | 0.311062 |
| TMED8 | Q6PL24 | -1.160334 | -1.744757 | -0.575911 | -3.903526 | 0.000112 | 0.267790 |
| SARG | Q9BW04 | -1.153551 | -1.692837 | -0.614264 | -4.205506 | 0.000032 | 0.079313 |
| TIA1 | P31483 | -1.152032 | -1.771448 | -0.532615 | -3.656647 | 0.000291 | 0.679578 |
| CA13 | Q8N1Q1 | -1.145407 | -1.712891 | -0.577923 | -3.968325 | 0.000086 | 0.207451 |
| SRC | P12931 | -1.142984 | -1.809233 | -0.476736 | -3.372913 | 0.000818 | 1.000000 |
| ERP29 | P30040 | -1.140857 | -1.717151 | -0.564563 | -3.892139 | 0.000117 | 0.279712 |
| ZFYVE19 | Q96K21 | -1.135417 | -1.663242 | -0.607592 | -4.229283 | 0.000029 | 0.071797 |
| BCL2L1 | Q07817 | -1.110453 | -1.641110 | -0.579796 | -4.114216 | 0.000047 | 0.115377 |
| FYB1 | O15117 | -1.110150 | -1.643498 | -0.576802 | -4.092342 | 0.000052 | 0.126065 |
| SIRT2 | Q8IXJ6 | -1.108455 | -1.636470 | -0.580440 | -4.127364 | 0.000045 | 0.109283 |
| CDK5RAP3 | Q96JB5 | -1.106959 | -1.628123 | -0.585795 | -4.175979 | 0.000037 | 0.089609 |
| PPIF | P30405 | -1.106505 | -1.774035 | -0.438976 | -3.259001 | 0.001216 | 1.000000 |
| SULT1A1 | P50225 | -1.101412 | -1.735680 | -0.467144 | -3.414115 | 0.000707 | 1.000000 |
| LYN | P07948 | -1.101025 | -1.538152 | -0.663898 | -4.952116 | 0.000001 | 0.002736 |
| PRKG1 | Q13976 | -1.100741 | -1.751736 | -0.449746 | -3.324366 | 0.000970 | 1.000000 |
| EIF4E | P06730 | -1.084233 | -1.736274 | -0.432191 | -3.269251 | 0.001174 | 1.000000 |
| TPD52L2 | O43399 | -1.079144 | -1.587802 | -0.570486 | -4.171141 | 0.000037 | 0.091337 |
| CMIP | Q8IY22 | -1.074182 | -1.735882 | -0.412481 | -3.191667 | 0.001529 | 1.000000 |
| RGS10 | O43665 | -1.066759 | -1.568612 | -0.564905 | -4.179172 | 0.000036 | 0.088450 |
| MINDY1 | Q8N5J2 | -1.065050 | -1.797527 | -0.332572 | -2.858755 | 0.004482 | 1.000000 |
| ATP6V1G2 | O95670 | -1.062623 | -1.657451 | -0.467795 | -3.512281 | 0.000496 | 1.000000 |
| NRGN | Q92686 | -1.061605 | -1.586709 | -0.536501 | -3.974831 | 0.000084 | 0.202162 |
| PSTPIP2 | Q9H939 | -1.058497 | -1.625796 | -0.491197 | -3.668411 | 0.000278 | 0.650903 |
| SPRY2 | O43597 | -1.054346 | -1.592535 | -0.516157 | -3.851672 | 0.000137 | 0.327241 |
| CDKN2D | P55273 | -1.046527 | -1.634151 | -0.458903 | -3.501483 | 0.000516 | 1.000000 |
| CLASP1 | Q7Z460 | -1.045862 | -1.713410 | -0.378315 | -3.080303 | 0.002215 | 1.000000 |
| CRKL | P46109 | -1.044492 | -1.581576 | -0.507408 | -3.823527 | 0.000153 | 0.364051 |
| ABRAXAS2 | Q15018 | -1.034553 | -1.493698 | -0.575409 | -4.430009 | 0.000012 | 0.030344 |
| ARHGEF12 | Q9NZN5 | -1.032577 | -1.533567 | -0.531587 | -4.052231 | 0.000061 | 0.148217 |
| TMEM132A | Q24JP5 | -1.031151 | -1.487913 | -0.574389 | -4.438475 | 0.000012 | 0.029265 |
| PAK4 | O96013 | -1.030811 | -1.523707 | -0.537916 | -4.111739 | 0.000048 | 0.116522 |
| SNX2 | O60749 | -1.030228 | -1.580853 | -0.479603 | -3.678564 | 0.000268 | 0.627178 |
| PTPN6 | P29350 | -1.022195 | -1.519312 | -0.525077 | -4.042736 | 0.000064 | 0.153969 |
| FHIT | P49789 | -1.018745 | -1.552323 | -0.485168 | -3.753783 | 0.000201 | 0.473181 |
| SMS | P52788 | -1.017387 | -1.545872 | -0.488902 | -3.784905 | 0.000178 | 0.420989 |
| MITD1 | Q8WV92 | -1.011066 | -1.550078 | -0.472053 | -3.687923 | 0.000258 | 0.605831 |
| SNAP25 | P60880 | -1.006607 | -1.253250 | -0.759964 | -8.024039 | 0.000000 | 0.000000 |
| RILPL2 | Q969X0 | -1.003281 | -1.519535 | -0.487026 | -3.820849 | 0.000155 | 0.367727 |
| NT5C | Q8TCD5 | -1.000693 | -1.468851 | -0.532534 | -4.202516 | 0.000033 | 0.080257 |
| SNAP23 | O00161 | -0.998992 | -1.550806 | -0.447177 | -3.559344 | 0.000418 | 0.966679 |
| BRAP | Q7Z569 | -0.997324 | -1.509369 | -0.485278 | -3.829385 | 0.000150 | 0.356101 |
| PDLIM5 | Q96HC4 | -0.992143 | -1.490760 | -0.493527 | -3.912090 | 0.000108 | 0.259030 |
| PLCB2 | Q00722 | -0.988504 | -1.668277 | -0.308730 | -2.859006 | 0.004478 | 1.000000 |
| CXCL5 | P42830 | -0.986528 | -1.580764 | -0.392292 | -3.264013 | 0.001196 | 1.000000 |
| VPS4B | O75351 | -0.983149 | -1.600243 | -0.366055 | -3.132344 | 0.001865 | 1.000000 |
| CLIP2 | Q9UDT6 | -0.981009 | -1.589404 | -0.372614 | -3.170215 | 0.001644 | 1.000000 |
| VTA1 | Q9NP79 | -0.980855 | -1.426335 | -0.535376 | -4.328909 | 0.000019 | 0.047077 |
| DOK2 | O60496 | -0.979904 | -1.579726 | -0.380082 | -3.211904 | 0.001428 | 1.000000 |
| PLA2G4A | P47712 | -0.975823 | -1.427676 | -0.523969 | -4.245944 | 0.000027 | 0.066938 |
| ATP5IF1 | Q9UII2 | -0.973825 | -1.585716 | -0.361934 | -3.129018 | 0.001886 | 1.000000 |
| ENO1 | P06733 | -0.973056 | -1.408882 | -0.537230 | -4.389606 | 0.000015 | 0.036184 |
| CRYZL1 | O95825 | -0.968220 | -1.443467 | -0.492973 | -4.005495 | 0.000074 | 0.178777 |
| DCTD | P32321 | -0.961550 | -1.464567 | -0.458532 | -3.758287 | 0.000197 | 0.466027 |
| STK4 | Q13043 | -0.955813 | -1.406530 | -0.505097 | -4.169375 | 0.000038 | 0.091979 |
| CASP7 | P55210 | -0.952270 | -1.413427 | -0.491113 | -4.059876 | 0.000059 | 0.143816 |
| IGBP1 | P78318 | -0.951575 | -1.505350 | -0.397800 | -3.378399 | 0.000803 | 1.000000 |
| TACC3 | Q9Y6A5 | -0.948402 | -1.546845 | -0.349959 | -3.115810 | 0.001970 | 1.000000 |
| SUGT1 | Q9Y2Z0 | -0.946088 | -1.407847 | -0.484328 | -4.028255 | 0.000068 | 0.163155 |
| MAP2K6 | P52564 | -0.943095 | -1.447173 | -0.439016 | -3.678395 | 0.000268 | 0.627312 |
| PNMA1 | Q8ND90 | -0.939086 | -1.430605 | -0.447566 | -3.756346 | 0.000199 | 0.468731 |
| ELAC1 | Q9H777 | -0.938917 | -1.395093 | -0.482740 | -4.046649 | 0.000063 | 0.151585 |
| TXNDC9 | O14530 | -0.935485 | -1.370531 | -0.500439 | -4.227689 | 0.000029 | 0.072255 |
| IL18RAP | O95256 | -0.934649 | -1.570222 | -0.299077 | -2.891244 | 0.004052 | 1.000000 |
| MDM1 | Q8TC05 | -0.932006 | -1.603799 | -0.260212 | -2.727622 | 0.006668 | 1.000000 |
| VASP | P50552 | -0.931009 | -1.382584 | -0.479434 | -4.053455 | 0.000061 | 0.147535 |
| IST1 | P53990 | -0.929564 | -1.398939 | -0.460189 | -3.893681 | 0.000116 | 0.278123 |
| ACADSB | P45954 | -0.927725 | -1.492413 | -0.363036 | -3.230065 | 0.001343 | 1.000000 |
| APPL2 | Q8NEU8 | -0.924665 | -1.539004 | -0.310327 | -2.959224 | 0.003273 | 1.000000 |
| PLEKHO1 | Q53GL0 | -0.923949 | -1.454418 | -0.393481 | -3.424443 | 0.000682 | 1.000000 |
| EIF4B | P23588 | -0.923298 | -1.359707 | -0.486889 | -4.159580 | 0.000039 | 0.095719 |
| CACNB3 | P54284 | -0.923192 | -1.425087 | -0.421297 | -3.616431 | 0.000338 | 0.787215 |
| PDAP1 | Q13442 | -0.923114 | -1.430229 | -0.415999 | -3.578903 | 0.000389 | 0.900454 |
| TADA3 | O75528 | -0.921477 | -1.385935 | -0.457019 | -3.900671 | 0.000113 | 0.270734 |
| CSDE1 | O75534 | -0.917869 | -1.514244 | -0.321494 | -3.025953 | 0.002643 | 1.000000 |
| DNAJC6 | O75061 | -0.917195 | -1.468391 | -0.365999 | -3.271573 | 0.001165 | 1.000000 |
| ERBIN | Q96RT1 | -0.915374 | -1.474541 | -0.356207 | -3.218536 | 0.001397 | 1.000000 |
| PCBP2 | Q15366 | -0.914874 | -1.415945 | -0.413803 | -3.589743 | 0.000373 | 0.866476 |
| RGCC | Q9H4X1 | -0.912242 | -1.385172 | -0.439312 | -3.792402 | 0.000173 | 0.409437 |
| SMARCA2 | P51531 | -0.905400 | -1.388617 | -0.422183 | -3.683831 | 0.000262 | 0.615045 |
| LAT | O43561 | -0.902669 | -1.339995 | -0.465343 | -4.058112 | 0.000060 | 0.144802 |
| HNRNPK | P61978 | -0.900130 | -1.569083 | -0.231177 | -2.645517 | 0.008487 | 1.000000 |
| SEC31A | O94979 | -0.897586 | -1.243391 | -0.551781 | -5.103238 | 0.000001 | 0.001308 |
| RAB33A | Q14088 | -0.894149 | -1.448931 | -0.339367 | -3.168754 | 0.001652 | 1.000000 |
| ANKMY2 | Q8IV38 | -0.891376 | -1.449448 | -0.333303 | -3.140299 | 0.001817 | 1.000000 |
| GTPBP2 | Q9BX10 | -0.891217 | -1.488570 | -0.293864 | -2.933282 | 0.003552 | 1.000000 |
| PTRHD1 | Q6GMV3 | -0.890717 | -1.327524 | -0.453911 | -4.009147 | 0.000073 | 0.176216 |
| TWF2 | Q6IBS0 | -0.883487 | -1.386617 | -0.380358 | -3.452403 | 0.000617 | 1.000000 |
| AKT1S1 | Q96B36 | -0.883440 | -1.336243 | -0.430637 | -3.835915 | 0.000146 | 0.347546 |
| ENO2 | P09104 | -0.881158 | -1.274154 | -0.488162 | -4.408255 | 0.000013 | 0.033382 |
| SMAD1 | Q15797 | -0.879202 | -1.378546 | -0.379857 | -3.461701 | 0.000596 | 1.000000 |
| JPT2 | Q9H910 | -0.878992 | -1.316989 | -0.440996 | -3.945622 | 0.000094 | 0.227001 |
| HS1BP3 | Q53T59 | -0.877556 | -1.287669 | -0.467443 | -4.207003 | 0.000032 | 0.078845 |
| KIFBP | Q96EK5 | -0.877484 | -1.349225 | -0.405743 | -3.657100 | 0.000290 | 0.678709 |
| FKBP14 | Q9NWM8 | -0.877046 | -1.349523 | -0.404568 | -3.649575 | 0.000299 | 0.697026 |
| HGS | O14964 | -0.875124 | -1.328292 | -0.421956 | -3.796745 | 0.000170 | 0.402926 |
| RBM17 | Q96I25 | -0.873026 | -1.367977 | -0.378074 | -3.467891 | 0.000583 | 1.000000 |
| CABP2 | Q9NPB3 | -0.872158 | -1.465930 | -0.278387 | -2.887866 | 0.004095 | 1.000000 |
| DENR | O43583 | -0.870358 | -1.277426 | -0.463289 | -4.203696 | 0.000033 | 0.079890 |
| DNAJC9 | Q8WXX5 | -0.867141 | -1.453921 | -0.280362 | -2.905469 | 0.003877 | 1.000000 |
| NUMB | P49757 | -0.865978 | -1.311830 | -0.420126 | -3.818714 | 0.000156 | 0.370494 |
| CDC37 | Q16543 | -0.863266 | -1.363727 | -0.362806 | -3.391376 | 0.000767 | 1.000000 |
| THTPA | Q9BU02 | -0.860344 | -1.336330 | -0.384358 | -3.553686 | 0.000427 | 0.985761 |
| CRADD | P78560 | -0.855483 | -1.310030 | -0.400937 | -3.700283 | 0.000246 | 0.578275 |
| CXCL3 | P19876 | -0.851585 | -1.395147 | -0.308024 | -3.080213 | 0.002215 | 1.000000 |
| USP47 | Q96K76 | -0.851147 | -1.310852 | -0.391442 | -3.640209 | 0.000309 | 0.721385 |
| ENSA | O43768 | -0.848545 | -1.324966 | -0.372124 | -3.501750 | 0.000516 | 1.000000 |
| USO1 | O60763 | -0.847973 | -1.314557 | -0.381389 | -3.573168 | 0.000397 | 0.918975 |
| ARF6 | P62330 | -0.843597 | -1.388906 | -0.298288 | -3.041542 | 0.002513 | 1.000000 |
| CNP | P09543 | -0.842276 | -1.257959 | -0.426593 | -3.983765 | 0.000081 | 0.195075 |
| TBCC | Q15814 | -0.841930 | -1.315709 | -0.368152 | -3.493831 | 0.000531 | 1.000000 |
| BCAN | Q96GW7 | -0.839241 | -1.024545 | -0.653937 | -8.904369 | 0.000000 | 0.000000 |
| NEFL | P07196 | -0.836193 | -1.259467 | -0.412919 | -3.884060 | 0.000121 | 0.288454 |
| TBCA | O75347 | -0.834784 | -1.389615 | -0.279953 | -2.958113 | 0.003284 | 1.000000 |
| MOG | Q16653 | -0.833661 | -1.068587 | -0.598736 | -6.976869 | 0.000000 | 0.000000 |
| CASP3 | P42574 | -0.830138 | -1.285604 | -0.374671 | -3.583396 | 0.000382 | 0.886353 |
| GMPR | P36959 | -0.829883 | -1.229749 | -0.430018 | -4.080417 | 0.000055 | 0.132265 |
| PPP1R14A | Q96A00 | -0.829155 | -1.266087 | -0.392222 | -3.730977 | 0.000219 | 0.515410 |
| SLITRK1 | Q96PX8 | -0.815144 | -1.040063 | -0.590224 | -7.125383 | 0.000000 | 0.000000 |
| MAX | P61244 | -0.811599 | -1.383090 | -0.240108 | -2.792116 | 0.005495 | 1.000000 |
| IL10 | P22301 | -0.809718 | -1.339017 | -0.280419 | -3.007695 | 0.002803 | 1.000000 |
| PACS2 | Q86VP3 | -0.808551 | -1.245297 | -0.371805 | -3.639821 | 0.000310 | 0.722126 |
| IMPACT | Q9P2X3 | -0.807532 | -1.231782 | -0.383282 | -3.742302 | 0.000210 | 0.493916 |
| PVALB | P20472 | -0.803351 | -1.229743 | -0.376959 | -3.704227 | 0.000243 | 0.569898 |
| KLK12 | Q9UKR0 | -0.800773 | -1.488110 | -0.113435 | -2.290552 | 0.022524 | 1.000000 |
| FXYD5 | Q96DB9 | -0.791926 | -1.191207 | -0.392644 | -3.899479 | 0.000114 | 0.271905 |
| TBL1X | O60907 | -0.786368 | -1.221458 | -0.351278 | -3.553432 | 0.000427 | 0.986256 |
| CRYM | Q14894 | -0.784409 | -1.247438 | -0.321380 | -3.330698 | 0.000949 | 1.000000 |
| ASRGL1 | Q7L266 | -0.782471 | -1.251316 | -0.313626 | -3.281257 | 0.001127 | 1.000000 |
| GOPC | Q9HD26 | -0.782062 | -1.266328 | -0.297796 | -3.175109 | 0.001617 | 1.000000 |
| YES1 | P07947 | -0.780631 | -1.301334 | -0.259929 | -2.947527 | 0.003396 | 1.000000 |
| VPS53 | Q5VIR6 | -0.775239 | -1.200549 | -0.349929 | -3.583694 | 0.000382 | 0.885758 |
| GRHPR | Q9UBQ7 | -0.773723 | -1.148395 | -0.399050 | -4.060082 | 0.000059 | 0.143754 |
| FRMD4B | Q9Y2L6 | -0.773411 | -1.418769 | -0.128052 | -2.356190 | 0.018959 | 1.000000 |
| LPP | Q93052 | -0.769954 | -1.111580 | -0.428328 | -4.431136 | 0.000012 | 0.030205 |
| GCC1 | Q96CN9 | -0.768439 | -1.306590 | -0.230289 | -2.807419 | 0.005245 | 1.000000 |
| PPIB | P23284 | -0.766251 | -1.195013 | -0.337490 | -3.513635 | 0.000494 | 1.000000 |
| ATG16L1 | Q676U5 | -0.755299 | -1.152928 | -0.357669 | -3.734572 | 0.000216 | 0.508571 |
| BCR | P11274 | -0.750036 | -1.194663 | -0.305409 | -3.316556 | 0.000997 | 1.000000 |
| NPPC | P23582 | -0.749259 | -1.083907 | -0.414611 | -4.401951 | 0.000014 | 0.034291 |
| KLK13 | Q9UKR3 | -0.747848 | -1.020529 | -0.475166 | -5.392113 | 0.000000 | 0.000303 |
| KLK6 | Q92876 | -0.747227 | -0.937344 | -0.557109 | -7.727377 | 0.000000 | 0.000000 |
| STK24 | Q9Y6E0 | -0.746570 | -1.220608 | -0.272533 | -3.096415 | 0.002101 | 1.000000 |
| ATG4A | Q8WYN0 | -0.744969 | -1.267893 | -0.222045 | -2.800922 | 0.005350 | 1.000000 |
| PARD3 | Q8TEW0 | -0.744022 | -1.350453 | -0.137591 | -2.412157 | 0.016320 | 1.000000 |
| LAT2 | Q9GZY6 | -0.742545 | -1.317464 | -0.167625 | -2.539315 | 0.011495 | 1.000000 |
| DTYMK | P23919 | -0.742502 | -1.236101 | -0.248902 | -2.957495 | 0.003291 | 1.000000 |
| DBNL | Q9UJU6 | -0.741777 | -1.233718 | -0.249837 | -2.964574 | 0.003218 | 1.000000 |
| LATS1 | O95835 | -0.741619 | -1.255894 | -0.227344 | -2.835219 | 0.004818 | 1.000000 |
| FKBP5 | Q13451 | -0.741239 | -1.202700 | -0.279777 | -3.158085 | 0.001712 | 1.000000 |
| CSPG5 | O95196 | -0.741163 | -0.989320 | -0.493007 | -5.872049 | 0.000000 | 0.000023 |
| FGD3 | Q5JSP0 | -0.741008 | -1.319899 | -0.162117 | -2.516678 | 0.012248 | 1.000000 |
| CMC1 | Q7Z7K0 | -0.740229 | -1.182503 | -0.297955 | -3.290607 | 0.001091 | 1.000000 |
| SYT1 | P21579 | -0.739392 | -0.999514 | -0.479270 | -5.588544 | 0.000000 | 0.000108 |
| PDE5A | O76074 | -0.738260 | -1.214782 | -0.261738 | -3.045982 | 0.002477 | 1.000000 |
| STAMBP | O95630 | -0.736751 | -1.146346 | -0.327156 | -3.536450 | 0.000454 | 1.000000 |
| CETN2 | P41208 | -0.736740 | -1.280059 | -0.193422 | -2.666006 | 0.007996 | 1.000000 |
| EBAG9 | O00559 | -0.735581 | -1.188584 | -0.282578 | -3.192495 | 0.001525 | 1.000000 |
| VAV3 | Q9UKW4 | -0.735470 | -1.353974 | -0.116966 | -2.337888 | 0.019899 | 1.000000 |
| FOXO3 | O43524 | -0.734632 | -1.401828 | -0.067435 | -2.164796 | 0.031011 | 1.000000 |
| YTHDF3 | Q7Z739 | -0.729819 | -1.270112 | -0.189525 | -2.655746 | 0.008239 | 1.000000 |
| CALB1 | P05937 | -0.728655 | -1.062288 | -0.395022 | -4.293920 | 0.000022 | 0.054662 |
| GMPR2 | Q9P2T1 | -0.727613 | -1.149377 | -0.305849 | -3.391812 | 0.000766 | 1.000000 |
| HPCAL1 | P37235 | -0.725495 | -1.108600 | -0.342390 | -3.723215 | 0.000226 | 0.530504 |
| WASF1 | Q92558 | -0.725414 | -1.277064 | -0.173765 | -2.585378 | 0.010090 | 1.000000 |
| DPP6 | P42658 | -0.724182 | -0.923301 | -0.525064 | -7.150520 | 0.000000 | 0.000000 |
| PCYT2 | Q99447 | -0.722101 | -1.114579 | -0.329624 | -3.617298 | 0.000337 | 0.785007 |
| TMCO5A | Q8N6Q1 | -0.721485 | -1.328309 | -0.114662 | -2.337578 | 0.019915 | 1.000000 |
| PTPRR | Q15256 | -0.719643 | -0.941306 | -0.497979 | -6.382988 | 0.000000 | 0.000001 |
| PDLIM7 | Q9NR12 | -0.717399 | -1.300179 | -0.134619 | -2.420234 | 0.015968 | 1.000000 |
| GPR158 | Q5T848 | -0.715902 | -1.006588 | -0.425215 | -4.842056 | 0.000002 | 0.004631 |
| STK11 | Q15831 | -0.715570 | -1.109188 | -0.321951 | -3.574192 | 0.000395 | 0.915896 |
| SCG3 | Q8WXD2 | -0.714448 | -0.925324 | -0.503572 | -6.661082 | 0.000000 | 0.000000 |
| CEP43 | O95684 | -0.713554 | -1.136118 | -0.290991 | -3.319990 | 0.000985 | 1.000000 |
| EIF4G1 | Q04637 | -0.712304 | -1.180320 | -0.244289 | -2.992309 | 0.002945 | 1.000000 |
| PLXNA4 | Q9HCM2 | -0.710520 | -1.134601 | -0.286440 | -3.294044 | 0.001078 | 1.000000 |
| RAB37 | Q96AX2 | -0.708002 | -1.245676 | -0.170327 | -2.588905 | 0.009989 | 1.000000 |
| ADD1 | P35611 | -0.707411 | -1.091077 | -0.323745 | -3.625099 | 0.000327 | 0.762769 |
| CHMP1A | Q9HD42 | -0.704570 | -1.111606 | -0.297535 | -3.403247 | 0.000735 | 1.000000 |
| ASPSCR1 | Q9BZE9 | -0.700734 | -1.064622 | -0.336846 | -3.786052 | 0.000177 | 0.419476 |
| OTUD6B | Q8N6M0 | -0.700714 | -1.132984 | -0.268444 | -3.187034 | 0.001554 | 1.000000 |
| ACOT13 | Q9NPJ3 | -0.700571 | -1.345264 | -0.055878 | -2.136488 | 0.033263 | 1.000000 |
| GMFG | O60234 | -0.699896 | -1.177116 | -0.222677 | -2.883480 | 0.004152 | 1.000000 |
| AK2 | P54819 | -0.699568 | -1.258531 | -0.140605 | -2.460639 | 0.014303 | 1.000000 |
| GYS1 | P13807 | -0.699155 | -1.182811 | -0.215498 | -2.842088 | 0.004718 | 1.000000 |
| UROD | P06132 | -0.698700 | -1.118354 | -0.279047 | -3.273416 | 0.001158 | 1.000000 |
| GRSF1 | Q12849 | -0.697032 | -1.216983 | -0.177080 | -2.635668 | 0.008733 | 1.000000 |
| ARHGEF1 | Q92888 | -0.696274 | -1.158108 | -0.234440 | -2.964116 | 0.003222 | 1.000000 |
| GLO1 | Q04760 | -0.696213 | -1.131043 | -0.261382 | -3.147917 | 0.001771 | 1.000000 |
| ATP6V1F | Q16864 | -0.691759 | -1.168688 | -0.214830 | -2.851688 | 0.004581 | 1.000000 |
| CACYBP | Q9HB71 | -0.691740 | -1.191213 | -0.192266 | -2.722898 | 0.006762 | 1.000000 |
| UGDH | O60701 | -0.691168 | -1.264645 | -0.117691 | -2.369567 | 0.018296 | 1.000000 |
| SMAD3 | P84022 | -0.690656 | -1.197373 | -0.183939 | -2.679770 | 0.007680 | 1.000000 |
| EIF5 | P55010 | -0.689366 | -1.201133 | -0.177599 | -2.648372 | 0.008417 | 1.000000 |
| PPP1R12A | O14974 | -0.687435 | -1.201234 | -0.173637 | -2.630511 | 0.008864 | 1.000000 |
| NDUFB7 | P17568 | -0.685377 | -1.110412 | -0.260341 | -3.170334 | 0.001643 | 1.000000 |
| BRD3 | Q15059 | -0.683754 | -1.309725 | -0.057782 | -2.147565 | 0.032366 | 1.000000 |
| NIT1 | Q86X76 | -0.682726 | -0.993835 | -0.371618 | -4.314560 | 0.000020 | 0.050030 |
| NAA10 | P41227 | -0.680079 | -1.185391 | -0.174768 | -2.646072 | 0.008474 | 1.000000 |
| NCAN | O14594 | -0.679649 | -0.903442 | -0.455855 | -5.970884 | 0.000000 | 0.000013 |
| CA2 | P00918 | -0.679438 | -1.231879 | -0.126998 | -2.418051 | 0.016062 | 1.000000 |
| RNF5 | Q99942 | -0.678876 | -1.093281 | -0.264470 | -3.220817 | 0.001386 | 1.000000 |
| IRAK1 | P51617 | -0.677352 | -1.057772 | -0.296933 | -3.500683 | 0.000518 | 1.000000 |
| ICA1 | Q05084 | -0.676140 | -1.123691 | -0.228590 | -2.970269 | 0.003160 | 1.000000 |
| CSRP3 | P50461 | -0.675108 | -1.335226 | -0.014990 | -2.010727 | 0.045044 | 1.000000 |
| PKN3 | Q6P5Z2 | -0.674126 | -1.190279 | -0.157974 | -2.567818 | 0.010607 | 1.000000 |
| NEXN | Q0ZGT2 | -0.670551 | -1.110209 | -0.230892 | -2.998592 | 0.002887 | 1.000000 |
| DDI2 | Q5TDH0 | -0.669856 | -1.146177 | -0.193535 | -2.764923 | 0.005965 | 1.000000 |
| FOXO1 | Q12778 | -0.669678 | -1.126091 | -0.213266 | -2.884759 | 0.004135 | 1.000000 |
| TXLNA | P40222 | -0.667340 | -1.063413 | -0.271267 | -3.312635 | 0.001011 | 1.000000 |
| BNIP2 | Q12982 | -0.665364 | -1.112456 | -0.218273 | -2.925933 | 0.003636 | 1.000000 |
| NPTN | Q9Y639 | -0.664966 | -1.004017 | -0.325916 | -3.855995 | 0.000135 | 0.321857 |
| RRM2B | Q7LG56 | -0.664774 | -1.023454 | -0.306095 | -3.643919 | 0.000305 | 0.711704 |
| GGACT | Q9BVM4 | -0.663130 | -1.024840 | -0.301419 | -3.604446 | 0.000353 | 0.821435 |
| COMT | P21964 | -0.661529 | -1.090491 | -0.232567 | -3.032016 | 0.002592 | 1.000000 |
| AKR1B1 | P15121 | -0.659616 | -1.054714 | -0.264518 | -3.282367 | 0.001122 | 1.000000 |
| HSPB1 | P04792 | -0.657053 | -1.003897 | -0.310210 | -3.724503 | 0.000225 | 0.528123 |
| AKT3 | Q9Y243 | -0.653669 | -1.027910 | -0.279428 | -3.434060 | 0.000659 | 1.000000 |
| ACOX1 | Q15067 | -0.653368 | -1.059122 | -0.247614 | -3.165893 | 0.001668 | 1.000000 |
| UBXN1 | Q04323 | -0.653357 | -1.078018 | -0.228697 | -3.024896 | 0.002652 | 1.000000 |
| PPME1 | Q9Y570 | -0.652710 | -1.141462 | -0.163957 | -2.625623 | 0.008990 | 1.000000 |
| UNC5D | Q6UXZ4 | -0.652077 | -0.864971 | -0.439183 | -6.021947 | 0.000000 | 0.000010 |
| ENAH | Q8N8S7 | -0.651847 | -0.884880 | -0.418813 | -5.499564 | 0.000000 | 0.000173 |
| DNAJC21 | Q5F1R6 | -0.651344 | -0.975282 | -0.327405 | -3.953201 | 0.000092 | 0.220289 |
| CRYGD | P07320 | -0.648572 | -1.179998 | -0.117147 | -2.399480 | 0.016888 | 1.000000 |
| KHK | P50053 | -0.648004 | -0.987797 | -0.308211 | -3.749418 | 0.000204 | 0.480994 |
| FIS1 | Q9Y3D6 | -0.646865 | -0.971426 | -0.322304 | -3.918485 | 0.000105 | 0.252720 |
| ZNRF4 | Q8WWF5 | -0.645145 | -0.996055 | -0.294235 | -3.614624 | 0.000340 | 0.791864 |
| RABGAP1L | Q5R372 | -0.644720 | -1.106265 | -0.183174 | -2.746362 | 0.006306 | 1.000000 |
| NSFL1C | Q9UNZ2 | -0.643872 | -1.088988 | -0.198757 | -2.843990 | 0.004690 | 1.000000 |
| IFT20 | Q8IY31 | -0.643254 | -0.998910 | -0.287598 | -3.555933 | 0.000423 | 0.978061 |
| MPHOSPH8 | Q99549 | -0.642245 | -1.060355 | -0.224134 | -3.020026 | 0.002694 | 1.000000 |
| MED18 | Q9BUE0 | -0.641836 | -1.025366 | -0.258305 | -3.290220 | 0.001092 | 1.000000 |
| LRTM2 | Q8N967 | -0.639648 | -0.813325 | -0.465970 | -7.241007 | 0.000000 | 0.000000 |
| GP6 | Q9HCN6 | -0.639132 | -0.986745 | -0.291519 | -3.614892 | 0.000340 | 0.791412 |
| TARS1 | P26639 | -0.637737 | -0.962760 | -0.312713 | -3.857695 | 0.000134 | 0.319843 |
| C2orf69 | Q8N8R5 | -0.636428 | -1.055911 | -0.216946 | -2.982889 | 0.003035 | 1.000000 |
| RAB11FIP3 | O75154 | -0.634768 | -1.184035 | -0.085501 | -2.272127 | 0.023624 | 1.000000 |
| MIF | P14174 | -0.634396 | -1.055199 | -0.213592 | -2.964027 | 0.003223 | 1.000000 |
| NECAP2 | Q9NVZ3 | -0.634082 | -1.073661 | -0.194503 | -2.836024 | 0.004807 | 1.000000 |
| GNAS | O95467 | -0.631738 | -0.932593 | -0.330883 | -4.128391 | 0.000045 | 0.108862 |
| IL11 | P20809 | -0.630200 | -1.003651 | -0.256748 | -3.317764 | 0.000993 | 1.000000 |
| AXIN1 | O15169 | -0.630112 | -1.085418 | -0.174806 | -2.720918 | 0.006802 | 1.000000 |
| REPS1 | Q96D71 | -0.629046 | -1.005395 | -0.252697 | -3.286191 | 0.001108 | 1.000000 |
| EIF4EBP1 | Q13541 | -0.628966 | -1.208302 | -0.049629 | -2.134504 | 0.033426 | 1.000000 |
| PPP1R2 | P41236 | -0.627076 | -1.024651 | -0.229501 | -3.101003 | 0.002069 | 1.000000 |
| SNAP29 | O95721 | -0.626039 | -1.075741 | -0.176337 | -2.737017 | 0.006484 | 1.000000 |
| AK1 | P00568 | -0.625644 | -1.142460 | -0.108829 | -2.380087 | 0.017790 | 1.000000 |
| ST13 | P50502 | -0.625021 | -0.988708 | -0.261334 | -3.378840 | 0.000801 | 1.000000 |
| IL22 | Q9GZX6 | -0.624829 | -1.081418 | -0.168239 | -2.690521 | 0.007441 | 1.000000 |
| DDAH1 | O94760 | -0.624091 | -0.923308 | -0.324874 | -4.100750 | 0.000050 | 0.121904 |
| DARS1 | P14868 | -0.623682 | -1.044060 | -0.203304 | -2.916922 | 0.003740 | 1.000000 |
| NOP56 | O00567 | -0.622375 | -1.092744 | -0.152006 | -2.601447 | 0.009637 | 1.000000 |
| STIP1 | P31948 | -0.622187 | -1.032211 | -0.212163 | -2.983411 | 0.003030 | 1.000000 |
| PSRC1 | Q6PGN9 | -0.621257 | -0.995261 | -0.247252 | -3.265845 | 0.001188 | 1.000000 |
| PARK7 | Q99497 | -0.619312 | -0.979133 | -0.259490 | -3.383941 | 0.000787 | 1.000000 |
| RWDD1 | Q9H446 | -0.616582 | -0.991719 | -0.241445 | -3.231487 | 0.001336 | 1.000000 |
| CNTN2 | Q02246 | -0.613526 | -0.837596 | -0.389457 | -5.383341 | 0.000000 | 0.000317 |
| MVK | Q03426 | -0.612616 | -1.042930 | -0.182302 | -2.799009 | 0.005381 | 1.000000 |
| MTDH | Q86UE4 | -0.611876 | -1.219610 | -0.004143 | -1.979482 | 0.048466 | 1.000000 |
| RILP | Q96NA2 | -0.611610 | -1.058682 | -0.164538 | -2.689667 | 0.007460 | 1.000000 |
| YOD1 | Q5VVQ6 | -0.610570 | -1.125851 | -0.095288 | -2.329656 | 0.020335 | 1.000000 |
| MYDGF | Q969H8 | -0.610052 | -1.035491 | -0.184612 | -2.819228 | 0.005060 | 1.000000 |
| IFNL1 | Q8IU54 | -0.609752 | -1.140428 | -0.079077 | -2.259049 | 0.024433 | 1.000000 |
| PIBF1 | Q8WXW3-4 | -0.607102 | -1.057382 | -0.156822 | -2.650817 | 0.008358 | 1.000000 |
| ARHGAP1 | Q07960 | -0.606774 | -0.897930 | -0.315618 | -4.097342 | 0.000051 | 0.123586 |
| CEND1 | Q8N111 | -0.606641 | -0.938662 | -0.274620 | -3.592256 | 0.000370 | 0.858798 |
| XIAP | P98170 | -0.606456 | -0.974779 | -0.238133 | -3.237213 | 0.001311 | 1.000000 |
| PRRT3 | Q5FWE3 | -0.604385 | -0.851121 | -0.357650 | -4.815966 | 0.000002 | 0.005235 |
| ZBTB16 | Q05516 | -0.603667 | -1.021581 | -0.185754 | -2.839962 | 0.004749 | 1.000000 |
| PTPRN2 | Q92932 | -0.603581 | -0.846594 | -0.360568 | -4.883236 | 0.000002 | 0.003807 |
| BECN1 | Q14457 | -0.603507 | -1.030707 | -0.176308 | -2.777493 | 0.005743 | 1.000000 |
| MSLNL | Q96KJ4 | -0.601584 | -1.179479 | -0.023689 | -2.046674 | 0.041362 | 1.000000 |
| NFX1 | Q12986 | -0.600145 | -1.047490 | -0.152800 | -2.637636 | 0.008683 | 1.000000 |
| OPLAH | O14841 | -0.599519 | -0.942100 | -0.256938 | -3.440652 | 0.000643 | 1.000000 |
| CIRBP | Q14011 | -0.599499 | -1.109658 | -0.089339 | -2.310378 | 0.021390 | 1.000000 |
| SEZ6L | Q9BYH1 | -0.597943 | -0.790033 | -0.405852 | -6.120051 | 0.000000 | 0.000006 |
| DNAJA2 | O60884 | -0.597809 | -0.953223 | -0.242395 | -3.306960 | 0.001031 | 1.000000 |
| ECI2 | O75521 | -0.595373 | -1.073764 | -0.116982 | -2.446848 | 0.014853 | 1.000000 |
| SEZ6 | Q53EL9 | -0.594323 | -0.876691 | -0.311956 | -4.138185 | 0.000043 | 0.104636 |
| SLC9A3R1 | O14745 | -0.593709 | -1.029776 | -0.157641 | -2.676832 | 0.007747 | 1.000000 |
| RPE | Q96AT9 | -0.591809 | -1.029749 | -0.153869 | -2.656858 | 0.008212 | 1.000000 |
| LACTB2 | Q53H82 | -0.588985 | -0.947666 | -0.230305 | -3.228477 | 0.001350 | 1.000000 |
| OMP | P47874 | -0.588899 | -1.026175 | -0.151624 | -2.647811 | 0.008431 | 1.000000 |
| PPM1F | P49593 | -0.587422 | -0.884742 | -0.290103 | -3.884438 | 0.000121 | 0.288142 |
| PLPBP | O94903 | -0.586471 | -1.008874 | -0.164067 | -2.729729 | 0.006627 | 1.000000 |
| SF3B4 | Q15427 | -0.586012 | -1.012313 | -0.159711 | -2.702661 | 0.007180 | 1.000000 |
| SHMT1 | P34896 | -0.585447 | -1.001009 | -0.169885 | -2.769831 | 0.005877 | 1.000000 |
| C9orf40 | Q8IXQ3 | -0.585297 | -1.093974 | -0.076619 | -2.262220 | 0.024235 | 1.000000 |
| GORASP2 | Q9H8Y8 | -0.585155 | -0.938080 | -0.232231 | -3.259798 | 0.001213 | 1.000000 |
| ATXN3 | P54252 | -0.582309 | -1.010770 | -0.153848 | -2.672042 | 0.007856 | 1.000000 |
| USP25 | Q9UHP3 | -0.581093 | -0.980895 | -0.181290 | -2.857600 | 0.004498 | 1.000000 |
| CCS | O14618 | -0.580663 | -0.934577 | -0.226748 | -3.225721 | 0.001363 | 1.000000 |
| HARS1 | P12081 | -0.578670 | -0.923574 | -0.233766 | -3.298633 | 0.001061 | 1.000000 |
| PPP1CC | P36873 | -0.577256 | -0.994341 | -0.160171 | -2.721104 | 0.006799 | 1.000000 |
| HEXIM1 | O94992 | -0.576781 | -0.956989 | -0.196573 | -2.982574 | 0.003038 | 1.000000 |
| TBC1D23 | Q9NUY8 | -0.573697 | -1.032343 | -0.115050 | -2.459267 | 0.014356 | 1.000000 |
| MANEAL | Q5VSG8 | -0.572697 | -0.923285 | -0.222108 | -3.211646 | 0.001430 | 1.000000 |
| SNX9 | Q9Y5X1 | -0.572461 | -0.902748 | -0.242173 | -3.407647 | 0.000724 | 1.000000 |
| KIAA0319 | Q5VV43 | -0.571814 | -0.757616 | -0.386013 | -6.050717 | 0.000000 | 0.000009 |
| CHM | P24386 | -0.571281 | -1.072310 | -0.070253 | -2.241758 | 0.025540 | 1.000000 |
| DDHD2 | O94830 | -0.570474 | -0.866731 | -0.274216 | -3.785885 | 0.000177 | 0.419571 |
| RANBP1 | P43487 | -0.570470 | -0.950725 | -0.190215 | -2.949575 | 0.003374 | 1.000000 |
| COX6B1 | P14854 | -0.568957 | -1.050962 | -0.086952 | -2.320753 | 0.020816 | 1.000000 |
| SOD1 | P00441 | -0.568144 | -0.924558 | -0.211729 | -3.134039 | 0.001855 | 1.000000 |
| TSC22D1 | Q15714 | -0.566108 | -0.899261 | -0.232954 | -3.340843 | 0.000916 | 1.000000 |
| NEK7 | Q8TDX7 | -0.564600 | -1.025276 | -0.103923 | -2.409606 | 0.016433 | 1.000000 |
| SH3GLB2 | Q9NR46 | -0.564077 | -1.004532 | -0.123623 | -2.517902 | 0.012206 | 1.000000 |
| USP8 | P40818 | -0.562185 | -1.078426 | -0.045945 | -2.141060 | 0.032890 | 1.000000 |
| BANK1 | Q8NDB2 | -0.561505 | -1.051279 | -0.071731 | -2.254026 | 0.024750 | 1.000000 |
| OTUD7B | Q6GQQ9 | -0.560942 | -0.883269 | -0.238616 | -3.421553 | 0.000689 | 1.000000 |
| INPPL1 | O15357 | -0.559801 | -1.011071 | -0.108531 | -2.438926 | 0.015177 | 1.000000 |
| NUDC | Q9Y266 | -0.556681 | -0.868705 | -0.244657 | -3.507678 | 0.000505 | 1.000000 |
| ACYP1 | P07311 | -0.556670 | -0.970009 | -0.143331 | -2.647843 | 0.008430 | 1.000000 |
| MOCS2 | O96007 | -0.556175 | -0.819038 | -0.293311 | -4.159896 | 0.000039 | 0.095632 |
| RNF43 | Q68DV7 | -0.556013 | -0.847843 | -0.264183 | -3.745903 | 0.000207 | 0.487334 |
| DFFA | O00273 | -0.554435 | -0.944728 | -0.164141 | -2.792933 | 0.005481 | 1.000000 |
| BRDT | Q58F21 | -0.552164 | -0.939119 | -0.165210 | -2.805493 | 0.005276 | 1.000000 |
| TPPP3 | Q9BW30 | -0.551552 | -0.953986 | -0.149118 | -2.694592 | 0.007353 | 1.000000 |
| SCPEP1 | Q9HB40 | -0.549207 | -0.830925 | -0.267490 | -3.832871 | 0.000148 | 0.351421 |
| GP1BB | P13224 | -0.548804 | -1.013139 | -0.084468 | -2.323732 | 0.020654 | 1.000000 |
| AGR2 | O95994 | -0.547995 | -1.049897 | -0.046094 | -2.146643 | 0.032439 | 1.000000 |
| GGCT | O75223 | -0.547816 | -0.934029 | -0.161603 | -2.788744 | 0.005551 | 1.000000 |
| SSNA1 | O43805 | -0.547779 | -1.019787 | -0.075771 | -2.281695 | 0.023047 | 1.000000 |
| RTBDN | Q9BSG5 | -0.547561 | -0.747222 | -0.347900 | -5.391893 | 0.000000 | 0.000304 |
| CHGB | P05060 | -0.545015 | -0.804266 | -0.285765 | -4.133239 | 0.000044 | 0.106729 |
| ATP6V1G1 | O75348 | -0.544834 | -1.003055 | -0.086613 | -2.337709 | 0.019909 | 1.000000 |
| STAT2 | P52630 | -0.544491 | -0.952657 | -0.136325 | -2.622736 | 0.009065 | 1.000000 |
| DMP1 | Q13316 | -0.542885 | -0.906125 | -0.179644 | -2.938427 | 0.003495 | 1.000000 |
| YWHAQ | P27348 | -0.542176 | -0.918509 | -0.165843 | -2.832496 | 0.004859 | 1.000000 |
| CC2D1A | Q6P1N0 | -0.541928 | -0.965413 | -0.118442 | -2.515961 | 0.012273 | 1.000000 |
| S100A16 | Q96FQ6 | -0.541588 | -0.852090 | -0.231085 | -3.429295 | 0.000670 | 1.000000 |
| FADD | Q13158 | -0.540635 | -0.975481 | -0.105790 | -2.444391 | 0.014953 | 1.000000 |
| CNPY4 | Q8N129 | -0.539115 | -0.861943 | -0.216287 | -3.283306 | 0.001119 | 1.000000 |
| SPOCK1 | Q08629 | -0.534433 | -0.686763 | -0.382103 | -6.897783 | 0.000000 | 0.000000 |
| DNPH1 | O43598 | -0.533596 | -0.900966 | -0.166227 | -2.855692 | 0.004524 | 1.000000 |
| PRKD2 | Q9BZL6 | -0.533314 | -0.819722 | -0.246906 | -3.660993 | 0.000286 | 0.669105 |
| PDIA4 | P13667 | -0.533205 | -0.864945 | -0.201465 | -3.160073 | 0.001701 | 1.000000 |
| TRIM25 | Q14258 | -0.532871 | -1.030948 | -0.034795 | -2.103427 | 0.036070 | 1.000000 |
| SLMAP | Q14BN4 | -0.532566 | -0.977882 | -0.087251 | -2.351293 | 0.019206 | 1.000000 |
| NIT2 | Q9NQR4 | -0.532189 | -0.929382 | -0.134995 | -2.634294 | 0.008768 | 1.000000 |
| S100A4 | P26447 | -0.531335 | -0.850794 | -0.211876 | -3.270050 | 0.001171 | 1.000000 |
| NFATC1 | O95644 | -0.530594 | -0.934346 | -0.126842 | -2.583740 | 0.010137 | 1.000000 |
| GCLM | P48507 | -0.530153 | -0.949027 | -0.111280 | -2.488396 | 0.013250 | 1.000000 |
| PRDX1 | Q06830 | -0.530059 | -0.893273 | -0.166844 | -2.869204 | 0.004339 | 1.000000 |
| PAPPA | Q13219 | -0.529656 | -0.838388 | -0.220923 | -3.372971 | 0.000818 | 1.000000 |
| SORD | Q00796 | -0.526729 | -1.013729 | -0.039729 | -2.126470 | 0.034093 | 1.000000 |
| CHAC2 | Q8WUX2 | -0.526347 | -0.934233 | -0.118461 | -2.537085 | 0.011568 | 1.000000 |
| UBE2L6 | O14933 | -0.525954 | -0.878522 | -0.173386 | -2.932959 | 0.003556 | 1.000000 |
| IL13 | P35225 | -0.525275 | -1.026088 | -0.024462 | -2.062111 | 0.039861 | 1.000000 |
| NPTXR | O95502 | -0.524565 | -0.696997 | -0.352133 | -5.981111 | 0.000000 | 0.000013 |
| RAB27B | O00194 | -0.524088 | -1.026538 | -0.021638 | -2.050750 | 0.040961 | 1.000000 |
| RAB10 | P61026 | -0.522140 | -0.884386 | -0.159895 | -2.833905 | 0.004838 | 1.000000 |
| NPY | P01303 | -0.521103 | -0.873931 | -0.168275 | -2.903767 | 0.003897 | 1.000000 |
| GLRX5 | Q86SX6 | -0.518073 | -0.966941 | -0.069205 | -2.269202 | 0.023803 | 1.000000 |
| UBE2Z | Q9H832 | -0.516716 | -0.846177 | -0.187255 | -3.083538 | 0.002191 | 1.000000 |
| EHBP1 | Q8NDI1 | -0.516004 | -0.918446 | -0.113561 | -2.520867 | 0.012106 | 1.000000 |
| TRIM24 | O15164 | -0.514406 | -0.864953 | -0.163860 | -2.885106 | 0.004131 | 1.000000 |
| TRDMT1 | O14717 | -0.514242 | -1.020573 | -0.007910 | -1.996796 | 0.046544 | 1.000000 |
| MPIG6B | O95866 | -0.512296 | -0.989358 | -0.035234 | -2.111286 | 0.035385 | 1.000000 |
| METAP2 | P50579 | -0.512197 | -0.824624 | -0.199770 | -3.223217 | 0.001374 | 1.000000 |
| MAPKAPK2 | P49137 | -0.511739 | -0.972976 | -0.050501 | -2.181346 | 0.029756 | 1.000000 |
| TXNDC5 | Q8NBS9 | -0.511536 | -0.862195 | -0.160877 | -2.868090 | 0.004354 | 1.000000 |
| CIAPIN1 | Q6FI81 | -0.508242 | -0.866584 | -0.149901 | -2.788525 | 0.005555 | 1.000000 |
| NUB1 | Q9Y5A7 | -0.506330 | -0.999746 | -0.012913 | -2.017533 | 0.044326 | 1.000000 |
| GIT1 | Q9Y2X7 | -0.506228 | -0.909198 | -0.103258 | -2.469875 | 0.013944 | 1.000000 |
| AHCY | P23526 | -0.503585 | -0.892872 | -0.114298 | -2.543341 | 0.011366 | 1.000000 |
| IL7 | P13232 | -0.502524 | -0.825815 | -0.179234 | -3.056081 | 0.002397 | 1.000000 |
| LZTFL1 | Q9NQ48 | -0.501857 | -0.888842 | -0.114871 | -2.549684 | 0.011165 | 1.000000 |
| IGSF21 | Q96ID5 | -0.501741 | -0.709186 | -0.294297 | -4.755311 | 0.000003 | 0.006955 |
| DECR1 | Q16698 | -0.501335 | -0.994844 | -0.007826 | -1.997259 | 0.046493 | 1.000000 |
| CCL5 | P13501 | -0.501191 | -0.969082 | -0.033301 | -2.106010 | 0.035844 | 1.000000 |
| ELAVL4 | P26378 | -0.499353 | -0.885332 | -0.113373 | -2.543574 | 0.011359 | 1.000000 |
| ARHGAP25 | P42331 | -0.499004 | -0.837092 | -0.160917 | -2.901860 | 0.003920 | 1.000000 |
| ZPR1 | O75312 | -0.498059 | -0.883020 | -0.113098 | -2.543693 | 0.011355 | 1.000000 |
| TP53I3 | Q53FA7 | -0.497648 | -0.813622 | -0.181673 | -3.096499 | 0.002100 | 1.000000 |
| PEBP1 | P30086 | -0.496323 | -0.846500 | -0.146145 | -2.786618 | 0.005587 | 1.000000 |
| L3HYPDH | Q96EM0 | -0.495900 | -0.920507 | -0.071293 | -2.296194 | 0.022196 | 1.000000 |
| ATOX1 | O00244 | -0.495805 | -0.798050 | -0.193560 | -3.225169 | 0.001365 | 1.000000 |
| CD69 | Q07108 | -0.495576 | -0.972692 | -0.018459 | -2.042147 | 0.041811 | 1.000000 |
| TRIM58 | Q8NG06 | -0.492918 | -0.956228 | -0.029609 | -2.091728 | 0.037111 | 1.000000 |
| ARHGAP45 | Q92619 | -0.492096 | -0.959729 | -0.024463 | -2.068928 | 0.039213 | 1.000000 |
| FAM172A | Q8WUF8 | -0.491964 | -0.792663 | -0.191264 | -3.216635 | 0.001406 | 1.000000 |
| FKBPL | Q9UIM3 | -0.491925 | -0.904308 | -0.079541 | -2.345301 | 0.019513 | 1.000000 |
| SERPINA12 | Q8IW75 | -0.491816 | -0.973309 | -0.010323 | -2.008234 | 0.045309 | 1.000000 |
| PDCD5 | O14737 | -0.490658 | -0.884883 | -0.096434 | -2.447014 | 0.014846 | 1.000000 |
| NT5C1A | Q9BXI3 | -0.488985 | -0.936943 | -0.041027 | -2.146146 | 0.032479 | 1.000000 |
| DNAJB6 | O75190 | -0.488206 | -0.835984 | -0.140427 | -2.759951 | 0.006054 | 1.000000 |
| NFKB1 | P19838 | -0.487993 | -0.848571 | -0.127415 | -2.660823 | 0.008118 | 1.000000 |
| SRPK2 | P78362 | -0.487947 | -0.928352 | -0.047542 | -2.178321 | 0.029982 | 1.000000 |
| CNST | Q6PJW8 | -0.486654 | -0.962471 | -0.010837 | -2.010858 | 0.045030 | 1.000000 |
| RAB2B | Q8WUD1 | -0.486518 | -0.847978 | -0.125058 | -2.646302 | 0.008468 | 1.000000 |
| C19orf12 | Q9NSK7 | -0.483938 | -0.843846 | -0.124030 | -2.643622 | 0.008534 | 1.000000 |
| MRI1 | Q9BV20 | -0.480979 | -0.829672 | -0.132287 | -2.711971 | 0.006985 | 1.000000 |
| APRT | P07741 | -0.480657 | -0.840257 | -0.121057 | -2.627946 | 0.008930 | 1.000000 |
| ENPP6 | Q6UWR7 | -0.479689 | -0.653897 | -0.305481 | -5.413688 | 0.000000 | 0.000271 |
| GAL | P22466 | -0.478210 | -0.765338 | -0.191082 | -3.274496 | 0.001153 | 1.000000 |
| TSNAX | Q99598 | -0.476156 | -0.863962 | -0.088350 | -2.413994 | 0.016240 | 1.000000 |
| GLRX | P35754 | -0.470918 | -0.868700 | -0.073137 | -2.327566 | 0.020447 | 1.000000 |
| BOLA2 | Q9H3K6 | -0.470861 | -0.794744 | -0.146979 | -2.858294 | 0.004488 | 1.000000 |
| PLIN1 | O60240 | -0.468939 | -0.931747 | -0.006130 | -1.992122 | 0.047056 | 1.000000 |
| REXO2 | Q9Y3B8 | -0.468105 | -0.833526 | -0.102685 | -2.518558 | 0.012184 | 1.000000 |
| DNAJB2 | P25686 | -0.467200 | -0.865128 | -0.069271 | -2.308332 | 0.021504 | 1.000000 |
| HDGF | P51858 | -0.466908 | -0.902700 | -0.031116 | -2.106461 | 0.035804 | 1.000000 |
| RLN1 | P04808 | -0.466471 | -0.850202 | -0.082740 | -2.390006 | 0.017323 | 1.000000 |
| AP3S2 | P59780 | -0.464655 | -0.841106 | -0.088205 | -2.426744 | 0.015688 | 1.000000 |
| SUSD1 | Q6UWL2 | -0.463594 | -0.811787 | -0.115401 | -2.617697 | 0.009198 | 1.000000 |
| RAD23B | P54727 | -0.463214 | -0.744800 | -0.181628 | -3.234233 | 0.001324 | 1.000000 |
| FKBP4 | Q02790 | -0.462843 | -0.701857 | -0.223830 | -3.807262 | 0.000163 | 0.387094 |
| LARP1 | Q6PKG0 | -0.462807 | -0.905368 | -0.020245 | -2.056020 | 0.040447 | 1.000000 |
| NELL2 | Q99435 | -0.461977 | -0.609802 | -0.314151 | -6.144281 | 0.000000 | 0.000005 |
| CARHSP1 | Q9Y2V2 | -0.461764 | -0.827134 | -0.096394 | -2.484785 | 0.013383 | 1.000000 |
| CHMP6 | Q96FZ7 | -0.461488 | -0.720864 | -0.202112 | -3.498102 | 0.000523 | 1.000000 |
| PRKAR1A | P10644 | -0.460604 | -0.888572 | -0.032635 | -2.116006 | 0.034979 | 1.000000 |
| PSMG3 | Q9BT73 | -0.460349 | -0.902248 | -0.018450 | -2.048165 | 0.041215 | 1.000000 |
| SERPINB6 | P35237 | -0.459823 | -0.747890 | -0.171757 | -3.138341 | 0.001829 | 1.000000 |
| TARBP2 | Q15633 | -0.459590 | -0.892711 | -0.026469 | -2.086230 | 0.037609 | 1.000000 |
| ELOB | Q15370 | -0.458655 | -0.774831 | -0.142479 | -2.852055 | 0.004575 | 1.000000 |
| TRAF2 | Q12933 | -0.458364 | -0.829013 | -0.087716 | -2.431361 | 0.015493 | 1.000000 |
| CD33 | P20138 | -0.458283 | -0.802624 | -0.113943 | -2.616662 | 0.009225 | 1.000000 |
| MSRA | Q9UJ68 | -0.457005 | -0.822762 | -0.091249 | -2.456576 | 0.014463 | 1.000000 |
| PAGR1 | Q9BTK6 | -0.452948 | -0.848771 | -0.057124 | -2.249819 | 0.025019 | 1.000000 |
| GBP2 | P32456 | -0.452645 | -0.870579 | -0.034712 | -2.129373 | 0.033851 | 1.000000 |
| OGA | O60502 | -0.451825 | -0.816094 | -0.087555 | -2.438644 | 0.015189 | 1.000000 |
| BOLA1 | Q9Y3E2 | -0.449325 | -0.825140 | -0.073509 | -2.350644 | 0.019239 | 1.000000 |
| HSBP1 | O75506 | -0.449022 | -0.845898 | -0.052146 | -2.224405 | 0.026694 | 1.000000 |
| CETN3 | O15182 | -0.448306 | -0.769454 | -0.127158 | -2.744546 | 0.006340 | 1.000000 |
| RGL2 | O15211 | -0.447041 | -0.889188 | -0.004893 | -1.987839 | 0.047530 | 1.000000 |
| FGF12 | P61328 | -0.445190 | -0.674286 | -0.216094 | -3.820575 | 0.000155 | 0.367965 |
| STX1B | P61266 | -0.444881 | -0.770624 | -0.119138 | -2.685157 | 0.007560 | 1.000000 |
| RNF41 | Q9H4P4 | -0.444297 | -0.777792 | -0.110803 | -2.619304 | 0.009155 | 1.000000 |
| AMPD3 | Q01432 | -0.444146 | -0.865052 | -0.023241 | -2.074639 | 0.038677 | 1.000000 |
| TRIM5 | Q9C035 | -0.443532 | -0.852542 | -0.034523 | -2.132029 | 0.033630 | 1.000000 |
| SNX15 | Q9NRS6 | -0.442760 | -0.829612 | -0.055909 | -2.250222 | 0.024993 | 1.000000 |
| SYAP1 | Q96A49 | -0.439583 | -0.731397 | -0.147769 | -2.961666 | 0.003247 | 1.000000 |
| VGF | O15240 | -0.439459 | -0.630071 | -0.248846 | -4.532817 | 0.000008 | 0.019224 |
| FABP5 | Q01469 | -0.437145 | -0.813093 | -0.061196 | -2.286113 | 0.022784 | 1.000000 |
| MARS1 | P56192 | -0.436192 | -0.831207 | -0.041178 | -2.171032 | 0.030533 | 1.000000 |
| HS6ST2 | Q96MM7 | -0.436060 | -0.685239 | -0.186882 | -3.440622 | 0.000643 | 1.000000 |
| NUDT5 | Q9UKK9 | -0.432846 | -0.734137 | -0.131554 | -2.824540 | 0.004979 | 1.000000 |
| TRIM21 | P19474 | -0.432004 | -0.854942 | -0.009066 | -2.008226 | 0.045310 | 1.000000 |
| DPP10 | Q8N608 | -0.430995 | -0.653509 | -0.208481 | -3.808169 | 0.000163 | 0.385891 |
| LUZP2 | Q86TE4 | -0.428224 | -0.626087 | -0.230361 | -4.255076 | 0.000026 | 0.064406 |
| FXN | Q16595 | -0.428118 | -0.804283 | -0.051953 | -2.237621 | 0.025811 | 1.000000 |
| FKBP7 | Q9Y680 | -0.427458 | -0.762922 | -0.091993 | -2.505227 | 0.012645 | 1.000000 |
| ARL2BP | Q9Y2Y0 | -0.427280 | -0.750892 | -0.103667 | -2.595903 | 0.009791 | 1.000000 |
| HTRA2 | O43464 | -0.426803 | -0.717586 | -0.136019 | -2.885749 | 0.004122 | 1.000000 |
| DRG2 | P55039 | -0.426013 | -0.714424 | -0.137602 | -2.904104 | 0.003893 | 1.000000 |
| PTGDS | P41222 | -0.425747 | -0.654846 | -0.196648 | -3.653677 | 0.000294 | 0.686931 |
| PPCDC | Q96CD2 | -0.424080 | -0.733561 | -0.114598 | -2.694099 | 0.007364 | 1.000000 |
| DCTN1 | Q14203 | -0.423522 | -0.775154 | -0.071889 | -2.368034 | 0.018371 | 1.000000 |
| LMOD1 | P29536 | -0.422963 | -0.727715 | -0.118212 | -2.728718 | 0.006647 | 1.000000 |
| WFIKKN1 | Q96NZ8 | -0.422430 | -0.646280 | -0.198580 | -3.710218 | 0.000237 | 0.557268 |
| ACTA2 | P62736 | -0.422110 | -0.702944 | -0.141277 | -2.955140 | 0.003315 | 1.000000 |
| CPA2 | P48052 | -0.421381 | -0.748155 | -0.094608 | -2.535301 | 0.011626 | 1.000000 |
| AHSA1 | O95433 | -0.421261 | -0.798076 | -0.044445 | -2.197979 | 0.028539 | 1.000000 |
| COMMD1 | Q8N668 | -0.420238 | -0.743519 | -0.096957 | -2.555736 | 0.010976 | 1.000000 |
| SWAP70 | Q9UH65 | -0.419875 | -0.730506 | -0.109244 | -2.657517 | 0.008196 | 1.000000 |
| PDZD2 | O15018 | -0.419663 | -0.798332 | -0.040994 | -2.178926 | 0.029937 | 1.000000 |
| CRNN | Q9UBG3 | -0.418877 | -0.721289 | -0.116466 | -2.723264 | 0.006755 | 1.000000 |
| CNTNAP2 | Q9UHC6 | -0.417174 | -0.635511 | -0.198837 | -3.756562 | 0.000199 | 0.468541 |
| SERPINB9 | P50453 | -0.416068 | -0.608790 | -0.223346 | -4.244580 | 0.000027 | 0.067301 |
| ACP5 | P13686 | -0.415429 | -0.577270 | -0.253588 | -5.046720 | 0.000001 | 0.001727 |
| LMOD1 | P29536 | -0.415276 | -0.727964 | -0.102589 | -2.611125 | 0.009373 | 1.000000 |
| TMEM106A | Q96A25 | -0.412829 | -0.785362 | -0.040296 | -2.178745 | 0.029950 | 1.000000 |
| QDPR | P09417 | -0.411782 | -0.662918 | -0.160645 | -3.223725 | 0.001372 | 1.000000 |
| BAG6 | P46379 | -0.411663 | -0.609474 | -0.213851 | -4.091581 | 0.000052 | 0.126410 |
| LMOD1 | P29536 | -0.411515 | -0.717845 | -0.105186 | -2.641181 | 0.008595 | 1.000000 |
| CALCA | P01258 | -0.407599 | -0.810216 | -0.004982 | -1.990407 | 0.047245 | 1.000000 |
| PTPRZ1 | P23471 | -0.407526 | -0.590897 | -0.224155 | -4.369443 | 0.000016 | 0.039503 |
| LMOD1 | P29536 | -0.407501 | -0.719190 | -0.095813 | -2.570453 | 0.010528 | 1.000000 |
| NAP1L4 | Q99733 | -0.407204 | -0.707460 | -0.106948 | -2.666376 | 0.007987 | 1.000000 |
| CPPED1 | Q9BRF8 | -0.406478 | -0.804028 | -0.008927 | -2.010228 | 0.045097 | 1.000000 |
| FABP2 | P12104 | -0.406322 | -0.723408 | -0.089236 | -2.519382 | 0.012156 | 1.000000 |
| KYAT1 | Q16773 | -0.405809 | -0.728832 | -0.082786 | -2.469960 | 0.013941 | 1.000000 |
| IMPA1 | P29218 | -0.405317 | -0.730991 | -0.079644 | -2.446888 | 0.014851 | 1.000000 |
| GALNT3 | Q14435 | -0.404660 | -0.622581 | -0.186740 | -3.650844 | 0.000297 | 0.694001 |
| DHRS4L2 | Q6PKH6 | -0.403881 | -0.704616 | -0.103147 | -2.640412 | 0.008614 | 1.000000 |
| CORO6 | Q6QEF8 | -0.402259 | -0.692354 | -0.112164 | -2.726255 | 0.006695 | 1.000000 |
| VPS37A | Q8NEZ2 | -0.401439 | -0.797631 | -0.005248 | -1.992122 | 0.047056 | 1.000000 |
| MAP2K1 | Q02750 | -0.400962 | -0.801607 | -0.000317 | -1.967637 | 0.049820 | 1.000000 |
| CRYBB1 | P53674 | -0.400742 | -0.673819 | -0.127665 | -2.885237 | 0.004129 | 1.000000 |
| IGF2BP3 | O00425 | -0.398724 | -0.729253 | -0.068195 | -2.371722 | 0.018191 | 1.000000 |
| VWC2L | B2RUY7 | -0.396085 | -0.594431 | -0.197738 | -3.926123 | 0.000102 | 0.245231 |
| BLOC1S3 | Q6QNY0 | -0.395489 | -0.704933 | -0.086046 | -2.512783 | 0.012382 | 1.000000 |
| DNM3 | Q9UQ16 | -0.395230 | -0.711000 | -0.079460 | -2.460824 | 0.014295 | 1.000000 |
| CCT5 | P48643 | -0.395087 | -0.708552 | -0.081623 | -2.478028 | 0.013635 | 1.000000 |
| CASP2 | P42575 | -0.395059 | -0.744125 | -0.045994 | -2.225134 | 0.026645 | 1.000000 |
| JAM3 | Q9BX67 | -0.394532 | -0.651663 | -0.137402 | -3.016689 | 0.002723 | 1.000000 |
| BRK1 | Q8WUW1 | -0.392676 | -0.619074 | -0.166278 | -3.410069 | 0.000717 | 1.000000 |
| PRDX6 | P30041 | -0.392313 | -0.705474 | -0.079152 | -2.463008 | 0.014210 | 1.000000 |
| DKK3 | Q9UBP4 | -0.391248 | -0.574708 | -0.207789 | -4.192895 | 0.000034 | 0.083516 |
| NPL | Q9BXD5 | -0.389155 | -0.667317 | -0.110993 | -2.750587 | 0.006227 | 1.000000 |
| GFER | P55789 | -0.387310 | -0.727177 | -0.047443 | -2.240532 | 0.025620 | 1.000000 |
| CRTAP | O75718 | -0.386926 | -0.655612 | -0.118239 | -2.831279 | 0.004877 | 1.000000 |
| RRM2 | P31350 | -0.386720 | -0.711304 | -0.062136 | -2.342449 | 0.019661 | 1.000000 |
| NXPH3 | O95157 | -0.386394 | -0.578049 | -0.194740 | -3.963811 | 0.000088 | 0.211171 |
| DRAXIN | Q8NBI3 | -0.384694 | -0.631301 | -0.138088 | -3.066992 | 0.002313 | 1.000000 |
| SORCS2 | Q96PQ0 | -0.381068 | -0.650345 | -0.111791 | -2.782304 | 0.005660 | 1.000000 |
| IL34 | Q6ZMJ4 | -0.380737 | -0.643611 | -0.117863 | -2.847599 | 0.004639 | 1.000000 |
| GLOD4 | Q9HC38 | -0.379755 | -0.611806 | -0.147705 | -3.217536 | 0.001401 | 1.000000 |
| PGD | P52209 | -0.376138 | -0.717601 | -0.034675 | -2.165731 | 0.030939 | 1.000000 |
| TOMM20 | Q15388 | -0.375814 | -0.691018 | -0.060610 | -2.344135 | 0.019574 | 1.000000 |
| PSME2 | Q9UL46 | -0.375811 | -0.615908 | -0.135713 | -3.077386 | 0.002236 | 1.000000 |
| KLHL41 | O60662 | -0.375341 | -0.699654 | -0.051028 | -2.275426 | 0.023424 | 1.000000 |
| ADAMTS15 | Q8TE58 | -0.374602 | -0.717290 | -0.031913 | -2.149171 | 0.032237 | 1.000000 |
| KAZALD1 | Q96I82 | -0.372125 | -0.610466 | -0.133784 | -3.069664 | 0.002293 | 1.000000 |
| EDAR | Q9UNE0 | -0.370933 | -0.705644 | -0.036222 | -2.178848 | 0.029942 | 1.000000 |
| SUSD4 | Q5VX71 | -0.370860 | -0.625485 | -0.116235 | -2.863586 | 0.004416 | 1.000000 |
| CAPN3 | P20807 | -0.370839 | -0.625164 | -0.116513 | -2.866795 | 0.004372 | 1.000000 |
| PODXL2 | Q9NZ53 | -0.369766 | -0.528316 | -0.211215 | -4.585210 | 0.000006 | 0.015202 |
| CCER2 | I3L3R5 | -0.368913 | -0.698546 | -0.039280 | -2.200364 | 0.028368 | 1.000000 |
| CALCB | P10092 | -0.368843 | -0.608331 | -0.129355 | -3.028017 | 0.002626 | 1.000000 |
| PRKRA | O75569 | -0.368836 | -0.708660 | -0.029013 | -2.133935 | 0.033473 | 1.000000 |
| AIF1L | Q9BQI0 | -0.368283 | -0.641908 | -0.094659 | -2.646232 | 0.008470 | 1.000000 |
| B4GAT1 | O43505 | -0.367285 | -0.511245 | -0.223324 | -5.016046 | 0.000001 | 0.002006 |
| AMOTL2 | Q9Y2J4 | -0.365538 | -0.552213 | -0.178862 | -3.849866 | 0.000138 | 0.329297 |
| KLB | Q86Z14 | -0.365342 | -0.693331 | -0.037353 | -2.189988 | 0.029118 | 1.000000 |
| TMEM25 | Q86YD3 | -0.360988 | -0.606192 | -0.115784 | -2.894456 | 0.004012 | 1.000000 |
| VSIG10L | Q86VR7 | -0.360246 | -0.573326 | -0.147165 | -3.323967 | 0.000972 | 1.000000 |
| ALDH1A1 | P00352 | -0.359925 | -0.703329 | -0.016521 | -2.060664 | 0.039999 | 1.000000 |
| ADAM22 | Q9P0K1 | -0.359661 | -0.562657 | -0.156666 | -3.483444 | 0.000551 | 1.000000 |
| COMP | P49747 | -0.358627 | -0.554828 | -0.162426 | -3.593703 | 0.000368 | 0.854562 |
| EPHB6 | O15197 | -0.358489 | -0.582955 | -0.134023 | -3.139982 | 0.001819 | 1.000000 |
| AAMDC | Q9H7C9 | -0.353981 | -0.654187 | -0.053775 | -2.318260 | 0.020953 | 1.000000 |
| CDC26 | Q8NHZ8 | -0.353864 | -0.635822 | -0.071905 | -2.467470 | 0.014037 | 1.000000 |
| BCAT1 | P54687 | -0.353488 | -0.556569 | -0.150408 | -3.422220 | 0.000687 | 1.000000 |
| WFIKKN2 | Q8TEU8 | -0.352864 | -0.524566 | -0.181162 | -4.040480 | 0.000064 | 0.155333 |
| PCDHB15 | Q9Y5E8 | -0.351312 | -0.597288 | -0.105336 | -2.808024 | 0.005236 | 1.000000 |
| SCRN1 | Q12765 | -0.350665 | -0.683599 | -0.017732 | -2.070795 | 0.039037 | 1.000000 |
| NRCAM | Q92823 | -0.349824 | -0.493883 | -0.205764 | -4.774279 | 0.000003 | 0.006369 |
| SEZ6L2 | Q6UXD5 | -0.349029 | -0.499383 | -0.198675 | -4.564022 | 0.000007 | 0.016727 |
| EFCAB2 | Q5VUJ9 | -0.347441 | -0.604426 | -0.090455 | -2.658113 | 0.008182 | 1.000000 |
| MSTN | O14793 | -0.347361 | -0.668809 | -0.025912 | -2.124569 | 0.034253 | 1.000000 |
| TXNRD1 | Q16881 | -0.344260 | -0.591951 | -0.096568 | -2.732600 | 0.006570 | 1.000000 |
| F2R | P25116 | -0.343469 | -0.638231 | -0.048706 | -2.290949 | 0.022500 | 1.000000 |
| CHRDL2 | Q6WN34 | -0.342838 | -0.552982 | -0.132693 | -3.207538 | 0.001450 | 1.000000 |
| GNPDA2 | Q8TDQ7 | -0.342441 | -0.542510 | -0.142373 | -3.365184 | 0.000841 | 1.000000 |
| HS6ST1 | O60243 | -0.339933 | -0.570258 | -0.109608 | -2.901705 | 0.003922 | 1.000000 |
| ADGRE2 | Q9UHX3 | -0.338354 | -0.492455 | -0.184253 | -4.316840 | 0.000020 | 0.049559 |
| OXCT1 | P55809 | -0.337293 | -0.670432 | -0.004154 | -1.990598 | 0.047224 | 1.000000 |
| CDCP1 | Q9H5V8 | -0.335239 | -0.616245 | -0.054233 | -2.345528 | 0.019502 | 1.000000 |
| NFIC | P08651 | -0.333991 | -0.595029 | -0.072953 | -2.515548 | 0.012287 | 1.000000 |
| PSME1 | Q06323 | -0.333925 | -0.577897 | -0.089954 | -2.690984 | 0.007431 | 1.000000 |
| ISLR2 | Q6UXK2 | -0.333854 | -0.543246 | -0.124462 | -3.134716 | 0.001851 | 1.000000 |
| FNTA | P49354 | -0.333342 | -0.578724 | -0.087959 | -2.670838 | 0.007884 | 1.000000 |
| TFRC | P02786 | -0.330373 | -0.551339 | -0.109407 | -2.939545 | 0.003483 | 1.000000 |
| IL2RG | P31785 | -0.330320 | -0.561927 | -0.098713 | -2.804040 | 0.005300 | 1.000000 |
| EPHA4 | P54764 | -0.330219 | -0.510313 | -0.150126 | -3.604999 | 0.000353 | 0.820097 |
| TUBB3 | Q13509 | -0.329629 | -0.604656 | -0.054602 | -2.356415 | 0.018947 | 1.000000 |
| LRP1 | Q07954 | -0.329130 | -0.499222 | -0.159038 | -3.804381 | 0.000165 | 0.391297 |
| DMD | P11532 | -0.326745 | -0.640233 | -0.013257 | -2.049225 | 0.041110 | 1.000000 |
| MAGED1 | Q9Y5V3 | -0.326584 | -0.620679 | -0.032488 | -2.183269 | 0.029613 | 1.000000 |
| DAG1 | Q14118 | -0.324795 | -0.532782 | -0.116808 | -3.070251 | 0.002289 | 1.000000 |
| UBE2B | P63146 | -0.323586 | -0.611102 | -0.036070 | -2.212731 | 0.027496 | 1.000000 |
| SPP1 | P10451 | -0.322947 | -0.583430 | -0.062464 | -2.437547 | 0.015234 | 1.000000 |
| VPS28 | Q9UK41 | -0.322738 | -0.642301 | -0.003176 | -1.985617 | 0.047777 | 1.000000 |
| TSPYL1 | Q9H0U9 | -0.322041 | -0.591900 | -0.052182 | -2.346254 | 0.019464 | 1.000000 |
| BRSK2 | Q8IWQ3 | -0.321120 | -0.613470 | -0.028771 | -2.159569 | 0.031416 | 1.000000 |
| FZD8 | Q9H461 | -0.317757 | -0.635293 | -0.000220 | -1.967443 | 0.049842 | 1.000000 |
| PLIN3 | O60664 | -0.315829 | -0.568478 | -0.063181 | -2.457744 | 0.014417 | 1.000000 |
| GPC5 | P78333 | -0.315761 | -0.542887 | -0.088634 | -2.733328 | 0.006556 | 1.000000 |
| ITGB7 | P26010 | -0.314877 | -0.542960 | -0.086793 | -2.714236 | 0.006938 | 1.000000 |
| CPA4 | Q9UI42 | -0.313539 | -0.516547 | -0.110530 | -3.036535 | 0.002554 | 1.000000 |
| NAAA | Q02083 | -0.313384 | -0.557782 | -0.068986 | -2.521043 | 0.012100 | 1.000000 |
| COL9A1 | P20849 | -0.312417 | -0.603817 | -0.021016 | -2.107877 | 0.035681 | 1.000000 |
| CLTA | P09496 | -0.311838 | -0.538581 | -0.085095 | -2.703936 | 0.007153 | 1.000000 |
| PENK | P01210 | -0.311502 | -0.545795 | -0.077209 | -2.613979 | 0.009297 | 1.000000 |
| TREM2 | Q9NZC2 | -0.310671 | -0.560548 | -0.060794 | -2.444422 | 0.014952 | 1.000000 |
| RP2 | O75695 | -0.310659 | -0.516346 | -0.104972 | -2.969469 | 0.003168 | 1.000000 |
| DNER | Q8NFT8 | -0.308014 | -0.437537 | -0.178490 | -4.675441 | 0.000004 | 0.010067 |
| DBI | P07108 | -0.307731 | -0.595616 | -0.019847 | -2.101621 | 0.036229 | 1.000000 |
| DTNB | O60941 | -0.306720 | -0.577676 | -0.035763 | -2.225578 | 0.026615 | 1.000000 |
| NPDC1 | Q9NQX5 | -0.306591 | -0.580275 | -0.032908 | -2.202480 | 0.028217 | 1.000000 |
| THBS4 | P35443 | -0.306478 | -0.540925 | -0.072031 | -2.570137 | 0.010537 | 1.000000 |
| FAM171B | Q6P995 | -0.301747 | -0.446679 | -0.156815 | -4.093368 | 0.000052 | 0.125583 |
| TALDO1 | P37837 | -0.301287 | -0.574580 | -0.027994 | -2.167472 | 0.030805 | 1.000000 |
| KYNU | Q16719 | -0.300519 | -0.485756 | -0.115282 | -3.189666 | 0.001540 | 1.000000 |
| CTSF | Q9UBX1 | -0.298710 | -0.512485 | -0.084935 | -2.747228 | 0.006290 | 1.000000 |
| LEG1 | Q6P5S2 | -0.297658 | -0.590389 | -0.004928 | -1.999180 | 0.046284 | 1.000000 |
| CA12 | O43570 | -0.297592 | -0.491405 | -0.103778 | -3.018827 | 0.002705 | 1.000000 |
| DBN1 | Q16643 | -0.293434 | -0.539290 | -0.047578 | -2.346555 | 0.019449 | 1.000000 |
| ASPN | Q9BXN1 | -0.292825 | -0.493883 | -0.091768 | -2.863451 | 0.004417 | 1.000000 |
| FGFR2 | P21802 | -0.292256 | -0.413721 | -0.170791 | -4.730576 | 0.000003 | 0.007800 |
| CD200 | P41217 | -0.291825 | -0.441463 | -0.142188 | -3.834276 | 0.000147 | 0.349638 |
| HPGDS | O60760 | -0.287298 | -0.513075 | -0.061520 | -2.501801 | 0.012766 | 1.000000 |
| GOT1 | P17174 | -0.286377 | -0.527164 | -0.045590 | -2.338336 | 0.019876 | 1.000000 |
| CD276 | Q5ZPR3 | -0.285641 | -0.471462 | -0.099819 | -3.022216 | 0.002675 | 1.000000 |
| ADGRE5 | P48960 | -0.284933 | -0.449005 | -0.120861 | -3.414366 | 0.000707 | 1.000000 |
| ACRBP | Q8NEB7 | -0.284662 | -0.468161 | -0.101164 | -3.049988 | 0.002445 | 1.000000 |
| NEDD4L | Q96PU5 | -0.284337 | -0.534779 | -0.033894 | -2.232161 | 0.026173 | 1.000000 |
| CRELD2 | Q6UXH1 | -0.283417 | -0.519723 | -0.047111 | -2.358045 | 0.018866 | 1.000000 |
| PLXNB3 | Q9ULL4 | -0.282776 | -0.475190 | -0.090363 | -2.889403 | 0.004076 | 1.000000 |
| SSC5D | A1L4H1 | -0.282206 | -0.456172 | -0.108240 | -3.189357 | 0.001541 | 1.000000 |
| MERTK | Q12866 | -0.282088 | -0.441414 | -0.122763 | -3.480972 | 0.000556 | 1.000000 |
| PRSS53 | Q2L4Q9 | -0.281857 | -0.464442 | -0.099271 | -3.035034 | 0.002567 | 1.000000 |
| CX3CL1 | P78423 | -0.281836 | -0.489583 | -0.074090 | -2.667253 | 0.007967 | 1.000000 |
| SNAPIN | O95295 | -0.281037 | -0.556139 | -0.005934 | -2.008492 | 0.045282 | 1.000000 |
| TNFSF10 | P50591 | -0.280981 | -0.449753 | -0.112209 | -3.273233 | 0.001158 | 1.000000 |
| UBAC1 | Q9BSL1 | -0.280871 | -0.511437 | -0.050304 | -2.395029 | 0.017091 | 1.000000 |
| NCAM1 | P13591 | -0.280670 | -0.424959 | -0.136380 | -3.824391 | 0.000153 | 0.362976 |
| IL5RA | Q01344 | -0.279162 | -0.540970 | -0.017355 | -2.096407 | 0.036691 | 1.000000 |
| DSCAM | O60469 | -0.276516 | -0.491535 | -0.061497 | -2.528399 | 0.011853 | 1.000000 |
| TAFA5 | Q7Z5A7 | -0.274979 | -0.515641 | -0.034317 | -2.246428 | 0.025237 | 1.000000 |
| TMPRSS5 | Q9H3S3 | -0.273268 | -0.426273 | -0.120264 | -3.511453 | 0.000498 | 1.000000 |
| IMPG1 | Q17R60 | -0.273177 | -0.487572 | -0.058782 | -2.505131 | 0.012649 | 1.000000 |
| SCGN | O76038 | -0.272977 | -0.498204 | -0.047750 | -2.382905 | 0.017656 | 1.000000 |
| CEACAM6 | P40199 | -0.272514 | -0.468575 | -0.076453 | -2.732748 | 0.006567 | 1.000000 |
| FGFBP2 | Q9BYJ0 | -0.272474 | -0.492414 | -0.052534 | -2.435687 | 0.015312 | 1.000000 |
| EPGN | Q6UW88 | -0.271877 | -0.521526 | -0.022227 | -2.141126 | 0.032885 | 1.000000 |
| CHAD | O15335 | -0.270459 | -0.537681 | -0.003237 | -1.989898 | 0.047302 | 1.000000 |
| TNFSF9 | P41273 | -0.269791 | -0.524428 | -0.015154 | -2.083088 | 0.037896 | 1.000000 |
| DCBLD2 | Q96PD2 | -0.269561 | -0.459587 | -0.079535 | -2.788980 | 0.005547 | 1.000000 |
| ACAN | P16112 | -0.267452 | -0.401830 | -0.133073 | -3.913069 | 0.000108 | 0.258129 |
| TGFBR3 | Q03167 | -0.267263 | -0.469578 | -0.064948 | -2.597233 | 0.009754 | 1.000000 |
| APOM | O95445 | -0.267016 | -0.431127 | -0.102905 | -3.198899 | 0.001493 | 1.000000 |
| VSIR | Q9H7M9 | -0.266716 | -0.514113 | -0.019318 | -2.119601 | 0.034672 | 1.000000 |
| MSLN | Q13421 | -0.266026 | -0.506551 | -0.025500 | -2.174523 | 0.030268 | 1.000000 |
| SEPTIN9 | Q9UHD8 | -0.264847 | -0.459103 | -0.070590 | -2.680525 | 0.007663 | 1.000000 |
| ADAMTSL4 | Q6UY14 | -0.264747 | -0.481785 | -0.047708 | -2.398255 | 0.016944 | 1.000000 |
| SLITRK2 | Q9H156 | -0.262438 | -0.469411 | -0.055465 | -2.492951 | 0.013084 | 1.000000 |
| CD226 | Q15762 | -0.259886 | -0.485684 | -0.034088 | -2.262894 | 0.024193 | 1.000000 |
| TGFB1 | P01137 | -0.259688 | -0.443937 | -0.075439 | -2.771070 | 0.005855 | 1.000000 |
| SPINT2 | O43291 | -0.259171 | -0.495718 | -0.022624 | -2.154123 | 0.031844 | 1.000000 |
| OGFR | Q9NZT2 | -0.258319 | -0.478099 | -0.038539 | -2.310835 | 0.021364 | 1.000000 |
| BLMH | Q13867 | -0.257108 | -0.429014 | -0.085202 | -2.940532 | 0.003472 | 1.000000 |
| TNFSF8 | P32971 | -0.256686 | -0.444177 | -0.069195 | -2.691677 | 0.007416 | 1.000000 |
| KLK15 | Q9H2R5 | -0.256578 | -0.496330 | -0.016825 | -2.104057 | 0.036015 | 1.000000 |
| THOP1 | P52888 | -0.254813 | -0.491729 | -0.017897 | -2.114602 | 0.035099 | 1.000000 |
| ADA2 | Q9NZK5 | -0.254352 | -0.483360 | -0.025344 | -2.183661 | 0.029584 | 1.000000 |
| ROR1 | Q01973 | -0.252011 | -0.439889 | -0.064133 | -2.637204 | 0.008694 | 1.000000 |
| ISM1 | B1AKI9 | -0.250649 | -0.435125 | -0.066173 | -2.671323 | 0.007873 | 1.000000 |
| F3 | P13726 | -0.248717 | -0.400266 | -0.097169 | -3.226670 | 0.001358 | 1.000000 |
| CD8A | P01732 | -0.248404 | -0.493853 | -0.002955 | -1.989747 | 0.047318 | 1.000000 |
| KITLG | P21583 | -0.247122 | -0.456289 | -0.037954 | -2.322835 | 0.020703 | 1.000000 |
| LY75 | O60449 | -0.246493 | -0.431476 | -0.061510 | -2.619836 | 0.009141 | 1.000000 |
| SELP | P16109 | -0.244805 | -0.462298 | -0.027311 | -2.212963 | 0.027480 | 1.000000 |
| PON3 | Q15166 | -0.244550 | -0.384396 | -0.104704 | -3.438101 | 0.000649 | 1.000000 |
| LRRN1 | Q6UXK5 | -0.243328 | -0.460401 | -0.026254 | -2.203869 | 0.028119 | 1.000000 |
| ENPP5 | Q9UJA9 | -0.240268 | -0.437399 | -0.043137 | -2.396307 | 0.017033 | 1.000000 |
| ADAMTS8 | Q9UP79 | -0.238685 | -0.444397 | -0.032973 | -2.281215 | 0.023075 | 1.000000 |
| CLUL1 | Q15846 | -0.236657 | -0.440170 | -0.033143 | -2.286264 | 0.022776 | 1.000000 |
| NTRK2 | Q16620 | -0.235147 | -0.374273 | -0.096021 | -3.323023 | 0.000975 | 1.000000 |
| TPK1 | Q9H3S4 | -0.234217 | -0.393029 | -0.075404 | -2.899570 | 0.003949 | 1.000000 |
| PLXDC1 | Q8IUK5 | -0.234083 | -0.383393 | -0.084773 | -3.082342 | 0.002200 | 1.000000 |
| NAGA | P17050 | -0.233804 | -0.432489 | -0.035120 | -2.313607 | 0.021210 | 1.000000 |
| POSTN | Q15063 | -0.232579 | -0.416099 | -0.049060 | -2.491671 | 0.013130 | 1.000000 |
| THY1 | P04216 | -0.232330 | -0.445077 | -0.019583 | -2.147050 | 0.032407 | 1.000000 |
| OMD | Q99983 | -0.232316 | -0.440085 | -0.024547 | -2.198363 | 0.028512 | 1.000000 |
| WARS1 | P23381 | -0.230707 | -0.448988 | -0.012426 | -2.078001 | 0.038364 | 1.000000 |
| ANGPTL2 | Q9UKU9 | -0.229319 | -0.416145 | -0.042494 | -2.413266 | 0.016272 | 1.000000 |
| APOA1 | P02647 | -0.229062 | -0.368106 | -0.090017 | -3.238920 | 0.001303 | 1.000000 |
| CHL1 | O00533 | -0.226122 | -0.343943 | -0.108300 | -3.773278 | 0.000186 | 0.440191 |
| APOA4 | P06727 | -0.225745 | -0.429385 | -0.022105 | -2.179497 | 0.029894 | 1.000000 |
| CD28 | P10747 | -0.225668 | -0.425068 | -0.026268 | -2.225082 | 0.026649 | 1.000000 |
| GALNT7 | Q86SF2 | -0.225317 | -0.352183 | -0.098451 | -3.491809 | 0.000535 | 1.000000 |
| LGALS1 | P09382 | -0.224340 | -0.435932 | -0.012748 | -2.084535 | 0.037763 | 1.000000 |
| GPC1 | P35052 | -0.223983 | -0.377297 | -0.070669 | -2.872326 | 0.004298 | 1.000000 |
| CDON | Q4KMG0 | -0.223276 | -0.389708 | -0.056843 | -2.637578 | 0.008685 | 1.000000 |
| GFRA3 | O60609 | -0.223157 | -0.387133 | -0.059180 | -2.675655 | 0.007773 | 1.000000 |
| MCAM | P43121 | -0.223087 | -0.409644 | -0.036530 | -2.351067 | 0.019218 | 1.000000 |
| OSCAR | Q8IYS5 | -0.221647 | -0.388011 | -0.055282 | -2.619403 | 0.009153 | 1.000000 |
| CTSV | O60911 | -0.220217 | -0.420575 | -0.019859 | -2.160955 | 0.031308 | 1.000000 |
| CA14 | Q9ULX7 | -0.219314 | -0.431244 | -0.007383 | -2.034576 | 0.042571 | 1.000000 |
| PRG3 | Q9Y2Y8 | -0.219201 | -0.438305 | -0.000097 | -1.966952 | 0.049899 | 1.000000 |
| APOH | P02749 | -0.218360 | -0.396634 | -0.040086 | -2.408161 | 0.016497 | 1.000000 |
| CTHRC1 | Q96CG8 | -0.218353 | -0.402382 | -0.034325 | -2.332789 | 0.020168 | 1.000000 |
| TNFSF12 | O43508 | -0.217887 | -0.410409 | -0.025365 | -2.225117 | 0.026646 | 1.000000 |
| PLTP | P55058 | -0.217043 | -0.393961 | -0.040125 | -2.411983 | 0.016328 | 1.000000 |
| NTRK3 | Q16288 | -0.216948 | -0.341812 | -0.092085 | -3.416040 | 0.000702 | 1.000000 |
| RGMB | Q6NW40 | -0.216116 | -0.401487 | -0.030746 | -2.292179 | 0.022429 | 1.000000 |
| GAPDH | P04406 | -0.215932 | -0.389173 | -0.042691 | -2.450569 | 0.014703 | 1.000000 |
| DSG3 | P32926 | -0.215786 | -0.391011 | -0.040560 | -2.421175 | 0.015927 | 1.000000 |
| PTPRB | P23467 | -0.214296 | -0.348116 | -0.080476 | -3.148421 | 0.001768 | 1.000000 |
| S100A13 | Q99584 | -0.214210 | -0.422906 | -0.005513 | -2.018020 | 0.044275 | 1.000000 |
| CPE | P16870 | -0.213487 | -0.361407 | -0.065566 | -2.837546 | 0.004784 | 1.000000 |
| IL32 | P24001 | -0.213421 | -0.426082 | -0.000760 | -1.973104 | 0.049191 | 1.000000 |
| CBLN4 | Q9NTU7 | -0.213132 | -0.403480 | -0.022784 | -2.201418 | 0.028293 | 1.000000 |
| SIAE | Q9HAT2 | -0.212809 | -0.410487 | -0.015132 | -2.116577 | 0.034930 | 1.000000 |
| TNFRSF21 | O75509 | -0.212539 | -0.380542 | -0.044536 | -2.487266 | 0.013291 | 1.000000 |
| PGLYRP2 | Q96PD5 | -0.212077 | -0.362881 | -0.061273 | -2.764912 | 0.005965 | 1.000000 |
| CDH2 | P19022 | -0.211446 | -0.392360 | -0.030531 | -2.297879 | 0.022099 | 1.000000 |
| LRRC25 | Q8N386 | -0.211326 | -0.405114 | -0.017537 | -2.144005 | 0.032652 | 1.000000 |
| GFRA2 | O00451 | -0.210841 | -0.321148 | -0.100535 | -3.757996 | 0.000198 | 0.466353 |
| FST | P19883 | -0.210529 | -0.403651 | -0.017408 | -2.143302 | 0.032708 | 1.000000 |
| ASAH1 | Q13510 | -0.209417 | -0.405890 | -0.012944 | -2.095612 | 0.036762 | 1.000000 |
| P4HB | P07237 | -0.208052 | -0.381302 | -0.034802 | -2.361023 | 0.018717 | 1.000000 |
| SUSD5 | O60279 | -0.207822 | -0.381578 | -0.034066 | -2.351537 | 0.019194 | 1.000000 |
| PROCR | Q9UNN8 | -0.207708 | -0.394966 | -0.020451 | -2.180797 | 0.029797 | 1.000000 |
| CD248 | Q9HCU0 | -0.207545 | -0.410999 | -0.004090 | -2.005607 | 0.045590 | 1.000000 |
| IFNLR1 | Q8IU57 | -0.207521 | -0.384773 | -0.030269 | -2.301820 | 0.021873 | 1.000000 |
| SOD2 | P04179 | -0.207324 | -0.414121 | -0.000526 | -1.971084 | 0.049423 | 1.000000 |
| CD200R1 | Q8TD46 | -0.207156 | -0.370596 | -0.043717 | -2.491969 | 0.013119 | 1.000000 |
| SEMA4D | Q92854 | -0.207096 | -0.347566 | -0.066625 | -2.898597 | 0.003961 | 1.000000 |
| AHNAK2 | Q8IVF2 | -0.206845 | -0.392603 | -0.021088 | -2.189274 | 0.029170 | 1.000000 |
| MXRA8 | Q9BRK3 | -0.205120 | -0.370137 | -0.040102 | -2.443875 | 0.014974 | 1.000000 |
| IDUA | P35475 | -0.204140 | -0.383773 | -0.024507 | -2.234305 | 0.026030 | 1.000000 |
| ICOSLG | O75144 | -0.203829 | -0.314802 | -0.092856 | -3.611182 | 0.000345 | 0.801757 |
| HEG1 | Q9ULI3 | -0.203609 | -0.326985 | -0.080233 | -3.244639 | 0.001278 | 1.000000 |
| CNTN3 | Q9P232 | -0.203105 | -0.346098 | -0.060113 | -2.792602 | 0.005487 | 1.000000 |
| PDCD1LG2 | Q9BQ51 | -0.202574 | -0.386204 | -0.018944 | -2.168904 | 0.030695 | 1.000000 |
| CD109 | Q6YHK3 | -0.201835 | -0.356719 | -0.046952 | -2.562084 | 0.010780 | 1.000000 |
| IGF2R | P11717 | -0.200831 | -0.320634 | -0.081029 | -3.295839 | 0.001071 | 1.000000 |
| NRP2 | O60462 | -0.200599 | -0.359270 | -0.041928 | -2.485602 | 0.013352 | 1.000000 |
| CPM | P14384 | -0.199145 | -0.369859 | -0.028430 | -2.293507 | 0.022351 | 1.000000 |
| NCAM2 | O15394 | -0.198794 | -0.340926 | -0.056662 | -2.749876 | 0.006240 | 1.000000 |
| ADGRB3 | O60242 | -0.197249 | -0.374755 | -0.019743 | -2.184751 | 0.029503 | 1.000000 |
| MEGF10 | Q96KG7 | -0.195892 | -0.362484 | -0.029301 | -2.311880 | 0.021306 | 1.000000 |
| CPQ | Q9Y646 | -0.195585 | -0.366676 | -0.024494 | -2.247549 | 0.025165 | 1.000000 |
| PAM | P19021 | -0.195579 | -0.353611 | -0.037546 | -2.433189 | 0.015416 | 1.000000 |
| BCHE | P06276 | -0.193888 | -0.326705 | -0.061070 | -2.870100 | 0.004327 | 1.000000 |
| PLA2G7 | Q13093 | -0.193565 | -0.341175 | -0.045954 | -2.578163 | 0.010300 | 1.000000 |
| TIMM10 | P62072 | -0.193070 | -0.378884 | -0.007256 | -2.042858 | 0.041740 | 1.000000 |
| HSD11B1 | P28845 | -0.192866 | -0.374213 | -0.011518 | -2.090957 | 0.037180 | 1.000000 |
| GLIPR1 | P48060 | -0.192804 | -0.311676 | -0.073931 | -3.188847 | 0.001544 | 1.000000 |
| NFASC | O94856 | -0.190266 | -0.352444 | -0.028088 | -2.306586 | 0.021603 | 1.000000 |
| NOTCH2 | Q04721 | -0.189244 | -0.323158 | -0.055331 | -2.778428 | 0.005727 | 1.000000 |
| ADAM23 | O75077 | -0.189152 | -0.362734 | -0.015571 | -2.142448 | 0.032778 | 1.000000 |
| SMAD5 | Q99717 | -0.188777 | -0.304535 | -0.073018 | -3.206236 | 0.001456 | 1.000000 |
| LYPD3 | O95274 | -0.187924 | -0.344284 | -0.031564 | -2.362969 | 0.018620 | 1.000000 |
| PROC | P04070 | -0.187532 | -0.337914 | -0.037150 | -2.451769 | 0.014654 | 1.000000 |
| GLA | P06280 | -0.186562 | -0.340543 | -0.032582 | -2.382098 | 0.017694 | 1.000000 |
| IGDCC4 | Q8TDY8 | -0.186005 | -0.328019 | -0.043991 | -2.575094 | 0.010390 | 1.000000 |
| L1CAM | P32004 | -0.184688 | -0.326282 | -0.043095 | -2.564466 | 0.010708 | 1.000000 |
| CSF3R | Q99062 | -0.184300 | -0.353380 | -0.015220 | -2.143058 | 0.032728 | 1.000000 |
| NHLRC3 | Q5JS37 | -0.184110 | -0.332713 | -0.035507 | -2.435848 | 0.015305 | 1.000000 |
| SERPIND1 | P05546 | -0.182796 | -0.354082 | -0.011510 | -2.098197 | 0.036532 | 1.000000 |
| NEO1 | Q92859 | -0.177735 | -0.270741 | -0.084729 | -3.757193 | 0.000198 | 0.467601 |
| PRCP | P42785 | -0.177173 | -0.339561 | -0.014785 | -2.145083 | 0.032565 | 1.000000 |
| LY9 | Q9HBG7 | -0.175002 | -0.329426 | -0.020578 | -2.228075 | 0.026447 | 1.000000 |
| PLG | P00747 | -0.174368 | -0.314434 | -0.034301 | -2.447557 | 0.014824 | 1.000000 |
| MANSC1 | Q9H8J5 | -0.173387 | -0.338538 | -0.008236 | -2.064133 | 0.039668 | 1.000000 |
| CPOX | P36551 | -0.173347 | -0.337604 | -0.009089 | -2.074875 | 0.038655 | 1.000000 |
| GPNMB | Q14956 | -0.172632 | -0.309255 | -0.036009 | -2.484269 | 0.013402 | 1.000000 |
| CD86 | P42081 | -0.172547 | -0.328447 | -0.016646 | -2.176009 | 0.030156 | 1.000000 |
| SUSD2 | Q9UGT4 | -0.172332 | -0.314534 | -0.030130 | -2.382661 | 0.017667 | 1.000000 |
| IL6R | P08887 | -0.170991 | -0.312584 | -0.029398 | -2.374290 | 0.018067 | 1.000000 |
| PLAU | P00749 | -0.170430 | -0.304805 | -0.036055 | -2.493617 | 0.013059 | 1.000000 |
| ENTPD6 | O75354 | -0.169502 | -0.291982 | -0.047022 | -2.720897 | 0.006803 | 1.000000 |
| BOC | Q9BWV1 | -0.169024 | -0.295350 | -0.042699 | -2.630631 | 0.008861 | 1.000000 |
| ECE1 | P42892 | -0.168490 | -0.335212 | -0.001768 | -1.986926 | 0.047632 | 1.000000 |
| F7 | P08709 | -0.168342 | -0.334962 | -0.001722 | -1.986401 | 0.047690 | 1.000000 |
| CD244 | Q9BZW8 | -0.168315 | -0.308860 | -0.027770 | -2.354547 | 0.019041 | 1.000000 |
| TXNDC15 | Q96J42 | -0.167883 | -0.284020 | -0.051745 | -2.842066 | 0.004718 | 1.000000 |
| STC2 | O76061 | -0.167740 | -0.325062 | -0.010418 | -2.096279 | 0.036703 | 1.000000 |
| SEMA4C | Q9C0C4 | -0.167066 | -0.315748 | -0.018384 | -2.209175 | 0.027744 | 1.000000 |
| CNTN1 | Q12860 | -0.166363 | -0.305578 | -0.027148 | -2.349488 | 0.019298 | 1.000000 |
| GALNT10 | Q86SR1 | -0.165992 | -0.315788 | -0.016195 | -2.178644 | 0.029958 | 1.000000 |
| PAMR1 | Q6UXH9 | -0.165697 | -0.310022 | -0.021373 | -2.257233 | 0.024547 | 1.000000 |
| THBD | P07204 | -0.165546 | -0.327746 | -0.003346 | -2.006633 | 0.045480 | 1.000000 |
| ROBO1 | Q9Y6N7 | -0.164586 | -0.300149 | -0.029022 | -2.386987 | 0.017464 | 1.000000 |
| STX3 | Q13277 | -0.164061 | -0.303739 | -0.024383 | -2.309288 | 0.021451 | 1.000000 |
| PEAR1 | Q5VY43 | -0.164015 | -0.276219 | -0.051812 | -2.873948 | 0.004276 | 1.000000 |
| GSR | P00390 | -0.163105 | -0.289095 | -0.037115 | -2.545260 | 0.011305 | 1.000000 |
| CD164 | Q04900 | -0.162338 | -0.322111 | -0.002565 | -1.997640 | 0.046452 | 1.000000 |
| FETUB | Q9UGM5 | -0.161988 | -0.319603 | -0.004372 | -2.020618 | 0.044004 | 1.000000 |
| PKD1 | P98161 | -0.161844 | -0.308850 | -0.014838 | -2.164527 | 0.031032 | 1.000000 |
| FOLR2 | P14207 | -0.160955 | -0.305628 | -0.016281 | -2.187339 | 0.029312 | 1.000000 |
| LAMB1 | P07942 | -0.159626 | -0.300073 | -0.019179 | -2.234570 | 0.026013 | 1.000000 |
| MEGF9 | Q9H1U4 | -0.159205 | -0.261098 | -0.057313 | -3.071970 | 0.002276 | 1.000000 |
| VASN | Q6EMK4 | -0.158302 | -0.296186 | -0.020419 | -2.257233 | 0.024547 | 1.000000 |
| CA11 | O75493 | -0.158138 | -0.279809 | -0.036467 | -2.555359 | 0.010987 | 1.000000 |
| SEMA7A | O75326 | -0.156184 | -0.311511 | -0.000858 | -1.976938 | 0.048754 | 1.000000 |
| CD84 | Q9UIB8 | -0.155717 | -0.297219 | -0.014215 | -2.163584 | 0.031104 | 1.000000 |
| DDR1 | Q08345 | -0.151821 | -0.280640 | -0.023003 | -2.317157 | 0.021014 | 1.000000 |
| GAS6 | Q14393 | -0.151537 | -0.294601 | -0.008472 | -2.082506 | 0.037949 | 1.000000 |
| CD99L2 | Q8TCZ2 | -0.151381 | -0.300612 | -0.002151 | -1.994413 | 0.046804 | 1.000000 |
| TGFBI | Q15582 | -0.151138 | -0.290604 | -0.011672 | -2.130626 | 0.033747 | 1.000000 |
| AMIGO2 | Q86SJ2 | -0.149145 | -0.245277 | -0.053014 | -3.050325 | 0.002442 | 1.000000 |
| C1R | P00736 | -0.149061 | -0.255185 | -0.042937 | -2.761538 | 0.006026 | 1.000000 |
| LEPR | P48357 | -0.148893 | -0.294172 | -0.003613 | -2.014981 | 0.044594 | 1.000000 |
| FSTL1 | Q12841 | -0.147845 | -0.261107 | -0.034584 | -2.566411 | 0.010649 | 1.000000 |
| ERBB4 | Q15303 | -0.147704 | -0.263591 | -0.031816 | -2.505856 | 0.012623 | 1.000000 |
| PRTG | Q2VWP7 | -0.147301 | -0.272280 | -0.022322 | -2.317240 | 0.021009 | 1.000000 |
| F13B | P05160 | -0.146936 | -0.264139 | -0.029733 | -2.464842 | 0.014139 | 1.000000 |
| RECK | O95980 | -0.144903 | -0.245000 | -0.044805 | -2.846122 | 0.004660 | 1.000000 |
| ICAM3 | P32942 | -0.143975 | -0.283607 | -0.004344 | -2.027250 | 0.043318 | 1.000000 |
| MEGF11 | A6BM72 | -0.140843 | -0.272154 | -0.009531 | -2.108789 | 0.035601 | 1.000000 |
| TYRO3 | Q06418 | -0.140689 | -0.277496 | -0.003883 | -2.021879 | 0.043873 | 1.000000 |
| NAGPA | Q9UK23 | -0.140525 | -0.255376 | -0.025675 | -2.405600 | 0.016612 | 1.000000 |
| PCDH12 | Q9NPG4 | -0.140288 | -0.267716 | -0.012860 | -2.164500 | 0.031034 | 1.000000 |
| PROS1 | P07225 | -0.140278 | -0.273441 | -0.007116 | -2.071138 | 0.039005 | 1.000000 |
| PON1 | P27169 | -0.137839 | -0.263844 | -0.011835 | -2.150739 | 0.032112 | 1.000000 |
| HYAL1 | Q12794 | -0.137154 | -0.252813 | -0.021495 | -2.331480 | 0.020238 | 1.000000 |
| CPB2 | Q96IY4 | -0.135423 | -0.257779 | -0.013067 | -2.176043 | 0.030153 | 1.000000 |
| CD34 | P28906 | -0.133088 | -0.261130 | -0.005045 | -2.043553 | 0.041671 | 1.000000 |
| CD58 | P19256 | -0.128318 | -0.224141 | -0.032496 | -2.632825 | 0.008805 | 1.000000 |
| ATRN | O75882-2 | -0.124526 | -0.247394 | -0.001657 | -1.992594 | 0.047004 | 1.000000 |
| ENTPD5 | O75356 | -0.123478 | -0.230670 | -0.016286 | -2.264791 | 0.024075 | 1.000000 |
| HYOU1 | Q9Y4L1 | -0.122875 | -0.238987 | -0.006762 | -2.080581 | 0.038126 | 1.000000 |
| LAMP1 | P11279 | -0.121523 | -0.237558 | -0.005488 | -2.059072 | 0.040152 | 1.000000 |
| ERP44 | Q9BS26 | -0.119984 | -0.239829 | -0.000139 | -1.968361 | 0.049736 | 1.000000 |
| GSN | P06396 | -0.119929 | -0.218105 | -0.021753 | -2.401715 | 0.016787 | 1.000000 |
| MENT | Q9BUN1 | -0.110994 | -0.214403 | -0.007585 | -2.110300 | 0.035470 | 1.000000 |
| CFP | P27918 | -0.108733 | -0.212944 | -0.004521 | -2.051384 | 0.040899 | 1.000000 |
| EGFR | P00533 | -0.106283 | -0.210578 | -0.001988 | -2.003565 | 0.045810 | 1.000000 |
| PCDH1 | Q08174 | -0.053895 | -0.097616 | -0.010174 | -2.423586 | 0.015823 | 1.000000 |
| SLC16A1 | P53985 | 0.213074 | 0.020568 | 0.405580 | 2.176142 | 0.030146 | 1.000000 |
| ADAMTSL5 | Q6ZMM2 | 0.222768 | 0.009637 | 0.435900 | 2.054981 | 0.040548 | 1.000000 |
| CCL23 | P55773 | 0.228150 | 0.008611 | 0.447688 | 2.043198 | 0.041706 | 1.000000 |
| TFPI2 | P48307 | 0.239260 | 0.000753 | 0.477766 | 1.972290 | 0.049284 | 1.000000 |
| GNLY | P22749 | 0.268575 | 0.045886 | 0.491265 | 2.371198 | 0.018217 | 1.000000 |
| ANGPT2 | O15123 | 0.284895 | 0.083984 | 0.485806 | 2.787929 | 0.005565 | 1.000000 |
| CCL14 | Q16627 | 0.288010 | 0.079629 | 0.496391 | 2.717386 | 0.006874 | 1.000000 |
| RAB6A | P20340 | 0.297699 | 0.024860 | 0.570538 | 2.145218 | 0.032554 | 1.000000 |
| LPCAT2 | Q7L5N7 | 0.318592 | 0.018246 | 0.618938 | 2.085520 | 0.037673 | 1.000000 |
| ITGA6 | P23229 | 0.319659 | 0.035059 | 0.604259 | 2.208275 | 0.027808 | 1.000000 |
| CCL7 | P80098 | 0.320253 | 0.029809 | 0.610696 | 2.167867 | 0.030775 | 1.000000 |
| AGER | Q15109 | 0.332677 | 0.055694 | 0.609659 | 2.361406 | 0.018698 | 1.000000 |
| CNDP1 | Q96KN2 | 0.341428 | 0.024171 | 0.658685 | 2.115871 | 0.034991 | 1.000000 |
| REN | P00797 | 0.407231 | 0.039549 | 0.774913 | 2.177556 | 0.030039 | 1.000000 |
| PLA2G2A | P14555 | 0.421432 | 0.062609 | 0.780255 | 2.309129 | 0.021460 | 1.000000 |
| ELOA | Q14241 | 0.423531 | 0.037844 | 0.809217 | 2.158996 | 0.031461 | 1.000000 |
| TPR | P12270 | 0.427258 | 0.026926 | 0.827589 | 2.098317 | 0.036521 | 1.000000 |
| NPM1 | P06748 | 0.444075 | 0.031868 | 0.856283 | 2.118077 | 0.034802 | 1.000000 |
| SIT1 | Q9Y3P8 | 0.472732 | 0.136540 | 0.808925 | 2.764577 | 0.005971 | 1.000000 |
| FGF23 | Q9GZV9 | 0.481304 | 0.032489 | 0.930118 | 2.108401 | 0.035635 | 1.000000 |
| MYH9 | P35579 | 0.498826 | 0.090101 | 0.907551 | 2.399492 | 0.016887 | 1.000000 |
| SART1 | O43290 | 0.501531 | 0.051891 | 0.951172 | 2.192974 | 0.028901 | 1.000000 |
| GPRC5C | Q9NQ84 | 0.513117 | 0.066603 | 0.959631 | 2.259345 | 0.024415 | 1.000000 |
| VIM | P08670 | 0.515588 | 0.132638 | 0.898537 | 2.647052 | 0.008450 | 1.000000 |
| DOC2B | Q14184 | 0.536093 | 0.028319 | 1.043867 | 2.075729 | 0.038575 | 1.000000 |
| NEDD9 | Q14511 | 0.566600 | 0.103552 | 1.029649 | 2.405755 | 0.016605 | 1.000000 |
| GZMH | P20718 | 0.570582 | 0.063641 | 1.077523 | 2.212900 | 0.027484 | 1.000000 |
| RAB44 | Q7Z6P3 | 0.591296 | 0.071576 | 1.111016 | 2.236847 | 0.025862 | 1.000000 |
| SMNDC1 | O75940 | 0.701110 | 0.193148 | 1.209071 | 2.713667 | 0.006950 | 1.000000 |
| NT-proBNP | NTproBNP | 0.758516 | 0.031575 | 1.485457 | 2.051477 | 0.040890 | 1.000000 |
| PADI4 | Q9UM07 | 0.758990 | 0.231817 | 1.286163 | 2.830635 | 0.004887 | 1.000000 |
| S100A12 | P80511 | 0.840987 | 0.405042 | 1.276932 | 3.792788 | 0.000173 | 0.408997 |
| IVD | P26440 | 1.017959 | 0.373098 | 1.662819 | 3.103600 | 0.002052 | 1.000000 |
| MNDA | P41218 | 1.053378 | 0.430868 | 1.675888 | 3.326895 | 0.000962 | 1.000000 |

**Table S3. Diagnostic performance of ischemia-enriched biomarkers.** Diagnostic accuracy metrics for proteins upregulated in ischemic stroke.

| **Biomarker** | **Cut-off** | **AUC (95% CI)** | **Sensitivity**  **(95% CI)** | **Specificity**  **(95% CI)** | **PPV**  **(95% CI)** | **NPV**  **(95% CI)** |
| --- | --- | --- | --- | --- | --- | --- |
| S100A12 | -0.209 | 0.675  (0.598 – 0.753) | 36.4  (23.8 – 51.1) | 35.8  (30.9 – 41.0) | 6.8  (4.2 – 10.7) | 81.5  (74.5–86.8) |
| MNDA | -0.339 | 0.657  (0.574 – 0.740) | 40.9  (27.7 – 55.6) | 33.7  (28.9 – 38.9) | 7.3  (4.7 – 11.3) | 81.7  (74.5–87.2) |
| PADI4 | 0.231 | 0.635  (0.574 – 0.740) | 22.7  (12.8 – 37.0) | 50.6  (45.3 – 55.8) | 5.6  (3.0 – 9.9) | 83.7  (78.0–88.1) |
| SMNDC1 | 0.399 | 0.613  (0.537 – 0.689) | 15.9  (7.9 – 29.4) | 58.1  (52.9 – 63.2) | 4.6  (2.3 – 9.3) | 84.4  (79.2–88.5) |
| NT-proBNP | 2.084 | 0.603  (0.526 – 0.680) | 4.5  (1.3 – 15.1) | 73.8  (68.9 – 78.2) | 2.2  (0.6 – 7.6) | 85.8  (81.4–89.3) |
| IVD | -0.027 | 0.602  (0.511 – 0.693) | 29.5  (18.2 – 44.2) | 48.8  (43.6 – 54.1) | 6.9  (4.1 – 11.4) | 84.4  (78.7–88.8) |

CI, confidence interval; PPV, positive predictive value; NPV, negative predictive value; AUC, area under the ROC curve.

**Table S4.** STRING functional enrichment computed from the subset of 68 differentially expressed proteins that remained significant after Bonferroni correction in the DEP analysis from Table S1 (ischemic vs hemorrhagic stroke). For each annotation term the table reports: term ID and description, observed gene count in the 68-protein query set, background gene count in the organism-wide reference used by STRING, enrichment strength defined as log10(observed/expected), the STRING “signal” metric provided as a measure of term specificity, and the false discovery rate (FDR) reported by STRING after multiple-testing correction. Only terms with FDR < 0.05 are shown. Identifier prefixes: GO biological process, GOCC cellular component, HSA Reactome, WP WikiPathways, BTO BRENDA Tissue Ontology, KW UniProt Keywords, IPR InterPro, SM SMART, CL STRING cluster labels. Counts are shown as “genes” to match STRING nomenclature although the input corresponds to proteins.

| **Term ID** | **Term description** | **Observed gene count** | **Background gene count** | **Strength** | **Signal** | **(FDR)** |
| --- | --- | --- | --- | --- | --- | --- |
| GO:0007399 | Nervous system development | 28 | 2188 | 0.57 | 0.73 | 3.59E-06 |
| GO:0031175 | Neuron projection development | 16 | 674 | 0.84 | 0.96 | 8.69E-06 |
| GO:0048699 | Generation of neurons | 19 | 1131 | 0.69 | 0.78 | 2.65E-05 |
| GO:0048731 | System development | 35 | 3867 | 0.42 | 0.55 | 2.65E-05 |
| GO:0022008 | Neurogenesis | 20 | 1290 | 0.65 | 0.75 | 2.77E-05 |
| GO:0007275 | Multicellular organism development | 36 | 4209 | 0.39 | 0.53 | 3.14E-05 |
| GO:0061564 | Axon development | 12 | 402 | 0.94 | 0.97 | 3.44E-05 |
| GO:0007409 | Axonogenesis | 11 | 363 | 0.94 | 0.91 | 9.78E-05 |
| GO:0030182 | Neuron differentiation | 17 | 1062 | 0.67 | 0.69 | 0.00015 |
| GO:0048858 | Cell projection morphogenesis | 12 | 487 | 0.85 | 0.81 | 0.00017 |
| GO:0120036 | Plasma membrane bounded cell projection organization | 17 | 1112 | 0.65 | 0.66 | 0.00023 |
| GO:0007417 | Central nervous system development | 16 | 1035 | 0.65 | 0.64 | 0.00038 |
| GO:0060074 | Synapse maturation | 4 | 17 | 1.83 | 1 | 0.00068 |
| GO:0048468 | Cell development | 20 | 1719 | 0.53 | 0.54 | 0.00073 |
| GO:0050808 | Synapse organization | 9 | 298 | 0.94 | 0.76 | 0.00077 |
| GO:0007155 | Cell adhesion | 14 | 965 | 0.62 | 0.52 | 0.003 |
| GO:0010001 | Glial cell differentiation | 7 | 188 | 1.03 | 0.68 | 0.003 |
| GO:0007411 | Axon guidance | 7 | 232 | 0.94 | 0.54 | 0.01 |
| GO:0050804 | Modulation of chemical synaptic transmission | 9 | 436 | 0.78 | 0.49 | 0.0109 |
| GO:0010975 | Regulation of neuron projection development | 9 | 442 | 0.77 | 0.48 | 0.0113 |
| GO:0006897 | Endocytosis | 9 | 447 | 0.77 | 0.48 | 0.0116 |
| GO:0023051 | Regulation of signaling | 26 | 3367 | 0.35 | 0.34 | 0.0124 |
| GO:0032501 | Multicellular organismal process | 39 | 6490 | 0.24 | 0.3 | 0.0133 |
| GO:0009987 | Cellular process | 64 | 14826 | 0.1 | 0.24 | 0.0198 |
| GO:0021782 | Glial cell development | 5 | 114 | 1.1 | 0.49 | 0.0219 |
| GO:0016477 | Cell migration | 12 | 903 | 0.59 | 0.38 | 0.0229 |
| GO:0048870 | Cell motility | 13 | 1061 | 0.55 | 0.37 | 0.0241 |
| GO:0010646 | Regulation of cell communication | 25 | 3355 | 0.33 | 0.3 | 0.0294 |
| GO:0031623 | Receptor internalization | 4 | 70 | 1.22 | 0.42 | 0.0438 |
| GO:0001764 | Neuron migration | 5 | 138 | 1.02 | 0.39 | 0.0465 |
| GO:0005539 | Glycosaminoglycan binding | 8 | 245 | 0.98 | 0.53 | 0.0121 |
| GO:0016020 | Membrane | 57 | 9523 | 0.24 | 0.44 | 2.87E-06 |
| GO:0045202 | Synapse | 20 | 1350 | 0.63 | 0.74 | 1.93E-05 |
| GO:0071944 | Cell periphery | 42 | 6015 | 0.31 | 0.45 | 7.55E-05 |
| GO:0030054 | Cell junction | 23 | 2115 | 0.5 | 0.57 | 0.00014 |
| GO:0098978 | Glutamatergic synapse | 10 | 334 | 0.94 | 0.88 | 0.00014 |
| GO:0005886 | Plasma membrane | 39 | 5544 | 0.31 | 0.44 | 0.00016 |
| GO:0012505 | Endomembrane system | 34 | 4721 | 0.32 | 0.41 | 0.0009 |
| GO:0097060 | Synaptic membrane | 9 | 375 | 0.84 | 0.66 | 0.0016 |
| GO:0012506 | Vesicle membrane | 15 | 1209 | 0.56 | 0.49 | 0.0031 |
| GO:0031982 | Vesicle | 29 | 3957 | 0.33 | 0.37 | 0.0042 |
| GO:0031225 | Anchored component of membrane | 6 | 171 | 1.01 | 0.6 | 0.0061 |
| GO:0030659 | Cytoplasmic vesicle membrane | 14 | 1190 | 0.53 | 0.43 | 0.0085 |
| GO:0098794 | Postsynapse | 10 | 621 | 0.67 | 0.47 | 0.0088 |
| GO:0031410 | Cytoplasmic vesicle | 21 | 2482 | 0.39 | 0.37 | 0.0093 |
| GO:0098588 | Bounding membrane of organelle | 19 | 2125 | 0.41 | 0.37 | 0.0099 |
| GO:0000015 | Phosphopyruvate hydratase complex | 2 | 4 | 2.16 | 0.55 | 0.0209 |
| GO:0072534 | Perineuronal net | 2 | 4 | 2.16 | 0.55 | 0.0209 |
| GO:0098590 | Plasma membrane region | 13 | 1237 | 0.48 | 0.33 | 0.0315 |
| GO:0005576 | Extracellular region | 27 | 4175 | 0.27 | 0.27 | 0.0387 |
| CL:23280 | Mixed, incl. Exocytic vesicle membrane, and Acetylcholine Neurotransmitter Release Cycle | 5 | 84 | 1.24 | 0.4 | 0.0497 |
| CL:28870 | Perisynaptic extracellular matrix, and Pleiotrophin / midkine family | 3 | 10 | 1.94 | 0.42 | 0.0497 |
| HSA-9607240 | FLT3 Signaling | 5 | 38 | 1.58 | 0.94 | 0.00082 |
| HSA-9006934 | Signaling by Receptor Tyrosine Kinases | 11 | 521 | 0.79 | 0.62 | 0.0021 |
| HSA-3595172 | Defective CHST3 causes SEDCJD | 3 | 8 | 2.04 | 0.75 | 0.0048 |
| HSA-3595174 | Defective CHST14 causes EDS, musculocontractural type | 3 | 8 | 2.04 | 0.75 | 0.0048 |
| HSA-3595177 | Defective CHSY1 causes TPBS | 3 | 8 | 2.04 | 0.75 | 0.0048 |
| HSA-2022923 | Dermatan sulfate biosynthesis | 3 | 11 | 1.9 | 0.73 | 0.0052 |
| HSA-1433557 | Signaling by SCF-KIT | 4 | 43 | 1.43 | 0.67 | 0.0067 |
| HSA-2024101 | CS/DS degradation | 3 | 14 | 1.79 | 0.68 | 0.0073 |
| HSA-2022870 | Chondroitin sulfate biosynthesis | 3 | 20 | 1.64 | 0.56 | 0.0167 |
| HSA-3560783 | Defective B4GALT7 causes EDS, progeroid type | 3 | 20 | 1.64 | 0.56 | 0.0167 |
| HSA-3560801 | Defective B3GAT3 causes JDSSDHD | 3 | 20 | 1.64 | 0.56 | 0.0167 |
| HSA-4420332 | Defective B3GALT6 causes EDSP2 and SEMDJL1 | 3 | 20 | 1.64 | 0.56 | 0.0167 |
| HSA-422475 | Axon guidance | 9 | 551 | 0.68 | 0.41 | 0.0215 |
| HSA-1971475 | A tetrasaccharide linker sequence is required for GAG synthesis | 3 | 26 | 1.52 | 0.52 | 0.0218 |
| HSA-389356 | CD28 co-stimulation | 3 | 33 | 1.42 | 0.45 | 0.0366 |
| WP2431 | Spinal cord injury | 5 | 115 | 1.1 | 0.4 | 0.0455 |
| BTO:0000142 | Brain | 45 | 5733 | 0.36 | 0.55 | 7.31E-07 |
| BTO:0000282 | Head | 48 | 6642 | 0.32 | 0.51 | 7.31E-07 |
| BTO:0001484 | Nervous system | 46 | 6016 | 0.35 | 0.54 | 7.31E-07 |
| BTO:0000445 | Cerebral lobe | 14 | 759 | 0.73 | 0.74 | 0.00015 |
| BTO:0001489 | Whole body | 63 | 13099 | 0.14 | 0.34 | 0.00015 |
| BTO:0001355 | Temporal lobe | 12 | 633 | 0.74 | 0.67 | 0.00062 |
| BTO:0000042 | Animal | 66 | 15148 | 0.1 | 0.29 | 0.0012 |
| BTO:0000233 | Cerebral cortex | 15 | 1118 | 0.59 | 0.55 | 0.0013 |
| BTO:0000900 | Myelin sheath | 3 | 9 | 1.98 | 0.89 | 0.0017 |
| BTO:0001001 | Parietal lobe | 4 | 33 | 1.55 | 0.85 | 0.0017 |
| BTO:0000293 | Occipital lobe | 4 | 36 | 1.51 | 0.84 | 0.0018 |
| BTO:0000478 | Forebrain | 17 | 1534 | 0.51 | 0.48 | 0.002 |
| BTO:0001042 | Amygdala | 7 | 226 | 0.95 | 0.69 | 0.002 |
| BTO:0000928 | Limbic system | 12 | 811 | 0.63 | 0.53 | 0.0027 |
| BTO:0000484 | Frontal lobe | 5 | 113 | 1.11 | 0.63 | 0.0059 |
| BTO:0002495 | Cerebral gyrus | 3 | 19 | 1.66 | 0.7 | 0.006 |
| BTO:0000601 | Hippocampus | 7 | 299 | 0.83 | 0.52 | 0.0087 |
| BTO:0000235 | Basal ganglion | 9 | 550 | 0.68 | 0.45 | 0.0117 |
| BTO:0001279 | Spinal cord | 6 | 233 | 0.87 | 0.48 | 0.016 |
| BTO:0004676 | Cerebral peduncle | 2 | 4 | 2.16 | 0.59 | 0.016 |
| BTO:0000894 | Myelin | 2 | 8 | 1.86 | 0.44 | 0.0431 |
| BTO:0000237 | Cerebrospinal fluid | 3 | 45 | 1.29 | 0.41 | 0.0472 |
| GOCC:0016020 | Membrane | 38 | 5715 | 0.28 | 0.33 | 0.008 |
| GOCC:0005737 | Cytoplasm | 46 | 8195 | 0.21 | 0.3 | 0.0081 |
| GOCC:0005886 | Plasma membrane | 28 | 3535 | 0.36 | 0.36 | 0.0081 |
| GOCC:0045202 | Synapse | 10 | 493 | 0.77 | 0.51 | 0.0081 |
| GOCC:0012505 | Endomembrane system | 25 | 3156 | 0.36 | 0.34 | 0.0132 |
| GOCC:0030659 | Cytoplasmic vesicle membrane | 11 | 723 | 0.64 | 0.43 | 0.0145 |
| GOCC:0030054 | Cell junction | 13 | 1053 | 0.55 | 0.4 | 0.0153 |
| GOCC:0031982 | Vesicle | 19 | 2125 | 0.41 | 0.34 | 0.0177 |
| GOCC:0098588 | Bounding membrane of organelle | 15 | 1451 | 0.48 | 0.35 | 0.023 |
| GOCC:0000015 | Phosphopyruvate hydratase complex | 2 | 5 | 2.06 | 0.44 | 0.0453 |
| GOCC:0031410 | Cytoplasmic vesicle | 16 | 1738 | 0.43 | 0.3 | 0.0453 |
| GOCC:0072534 | Perineuronal net | 2 | 5 | 2.06 | 0.44 | 0.0453 |
| GOCC:0110165 | Cellular anatomical entity | 61 | 14060 | 0.1 | 0.21 | 0.0453 |
| KW-0732 | Signal | 36 | 3277 | 0.5 | 0.76 | 6.18E-09 |
| KW-0325 | Glycoprotein | 38 | 4386 | 0.4 | 0.59 | 7.04E-07 |
| KW-0727 | SH2 domain | 7 | 110 | 1.27 | 1.19 | 3.42E-05 |
| KW-0654 | Proteoglycan | 5 | 55 | 1.42 | 1.02 | 0.00033 |
| KW-0472 | Membrane | 43 | 7068 | 0.25 | 0.38 | 0.00058 |
| KW-1003 | Cell membrane | 27 | 3277 | 0.38 | 0.45 | 0.0006 |
| KW-1015 | Disulfide bond | 27 | 3338 | 0.37 | 0.44 | 0.00073 |
| KW-0130 | Cell adhesion | 9 | 478 | 0.74 | 0.56 | 0.0035 |
| KW-0768 | Sushi | 4 | 57 | 1.31 | 0.7 | 0.0043 |
| KW-0336 | GPI-anchor | 5 | 138 | 1.02 | 0.57 | 0.009 |
| KW-0245 | EGF-like domain | 6 | 232 | 0.87 | 0.52 | 0.0103 |
| KW-0393 | Immunoglobulin domain | 8 | 463 | 0.7 | 0.46 | 0.0113 |
| KW-0770 | Synapse | 8 | 461 | 0.7 | 0.46 | 0.0113 |
| KW-0373 | Hyaluronic acid | 2 | 10 | 1.76 | 0.47 | 0.0361 |
| KW-0254 | Endocytosis | 4 | 128 | 0.96 | 0.38 | 0.0498 |
| IPR000980 | SH2 domain | 7 | 111 | 1.26 | 0.83 | 0.0012 |
| IPR036860 | SH2 domain superfamily | 7 | 115 | 1.25 | 0.83 | 0.0012 |
| SM00252 | Src homology 2 domains | 7 | 112 | 1.26 | 1.05 | 0.00014 |
| SM00032 | Domain abundant in complement control proteins | 4 | 56 | 1.32 | 0.51 | 0.0222 |
| SM01192 | Enolase, C-terminal TIM barrel domain | 2 | 4 | 2.16 | 0.43 | 0.0474 |
| SM01193 | Enolase, N-terminal domain | 2 | 4 | 2.16 | 0.43 | 0.0474 |
