# Supplementary material for: Plasma proteomics uncovers divergent molecular signatures in ischemic stroke and intracerebral hemorrhage
Source: Biomark Res. 2025 Oct 28;13:136. doi: 10.1186/s40364-025-00848-1 (PMC12570678; doi:10.1186/s40364-025-00848-1)
Supplement: Supplementary file 2 — Supplementary Material 2. [file 40364_2025_848_MOESM2_ESM.docx]

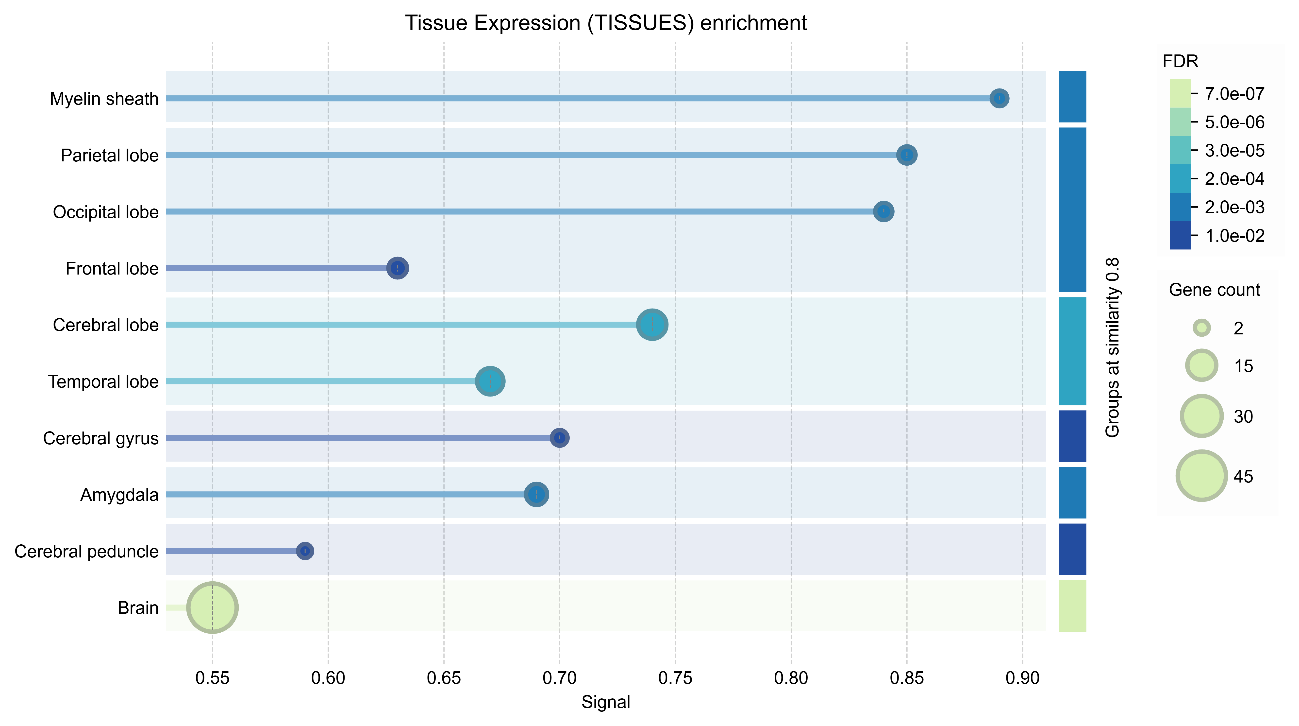


**Supplementary Figure S1.** **Functional enrichment analysis of differentially expressed proteins** **in tissue-specific.** Each dot represents a significantly enriched term, grouped by functional similarity. Dot size corresponds to the number of proteins associated with each term, while the x-axis indicates the enrichment signal score. Color intensity reflects the false discovery rate (FDR), ranging from light green (lowest FDR) to dark blue (higher FDR).


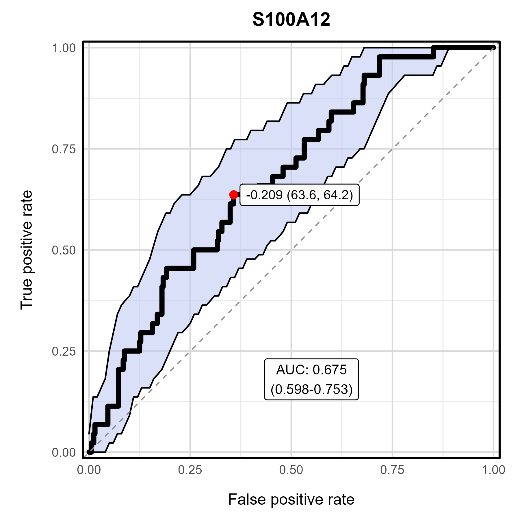

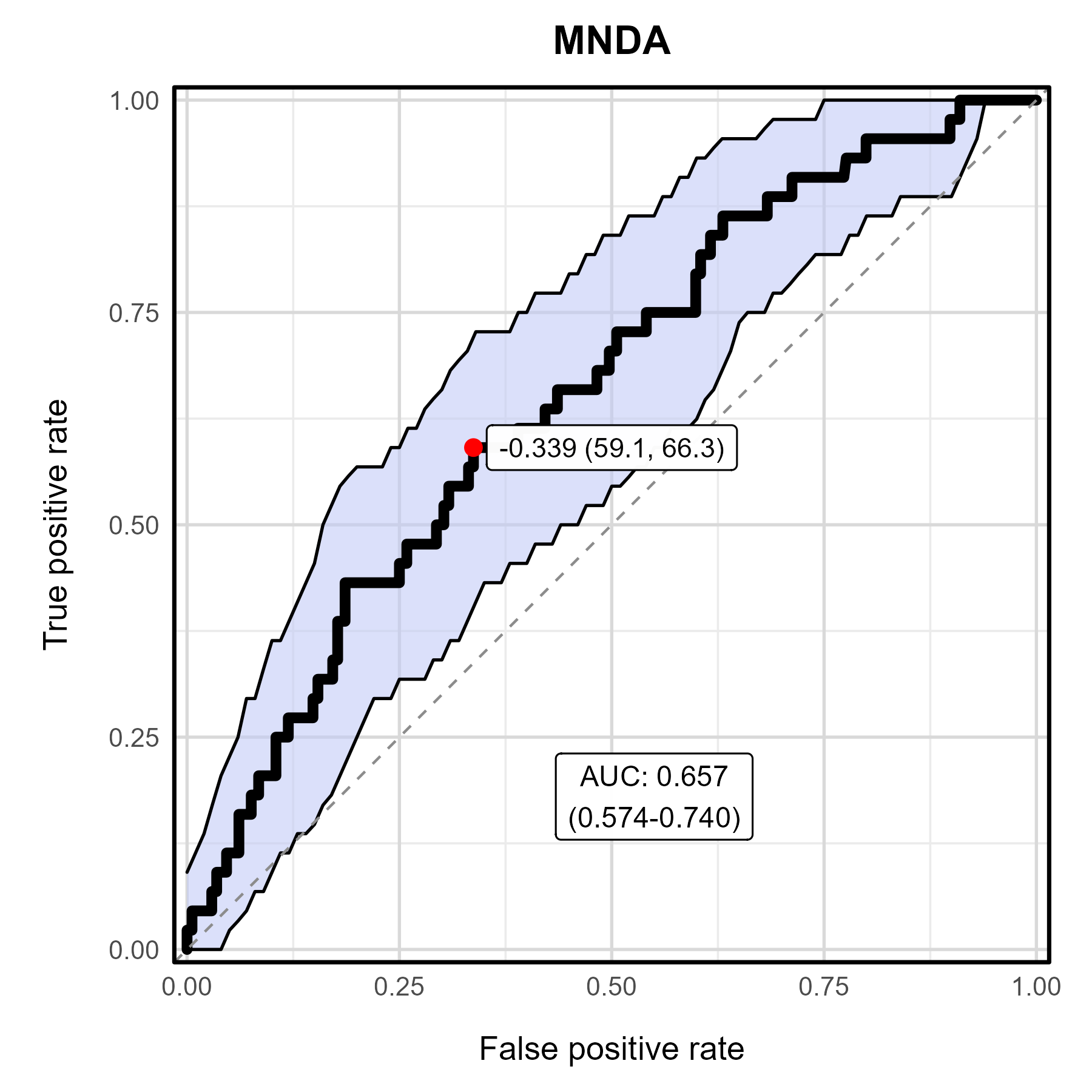

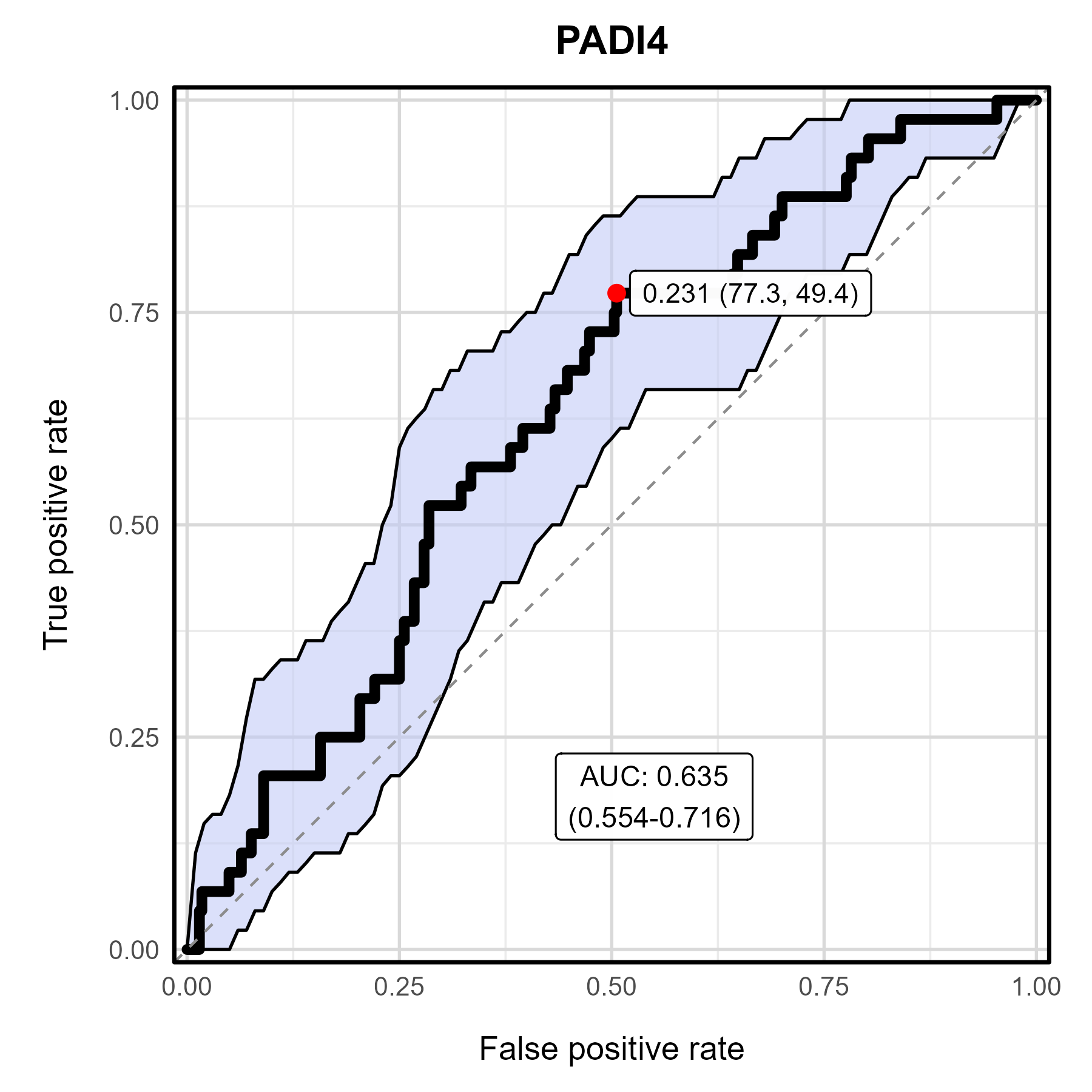

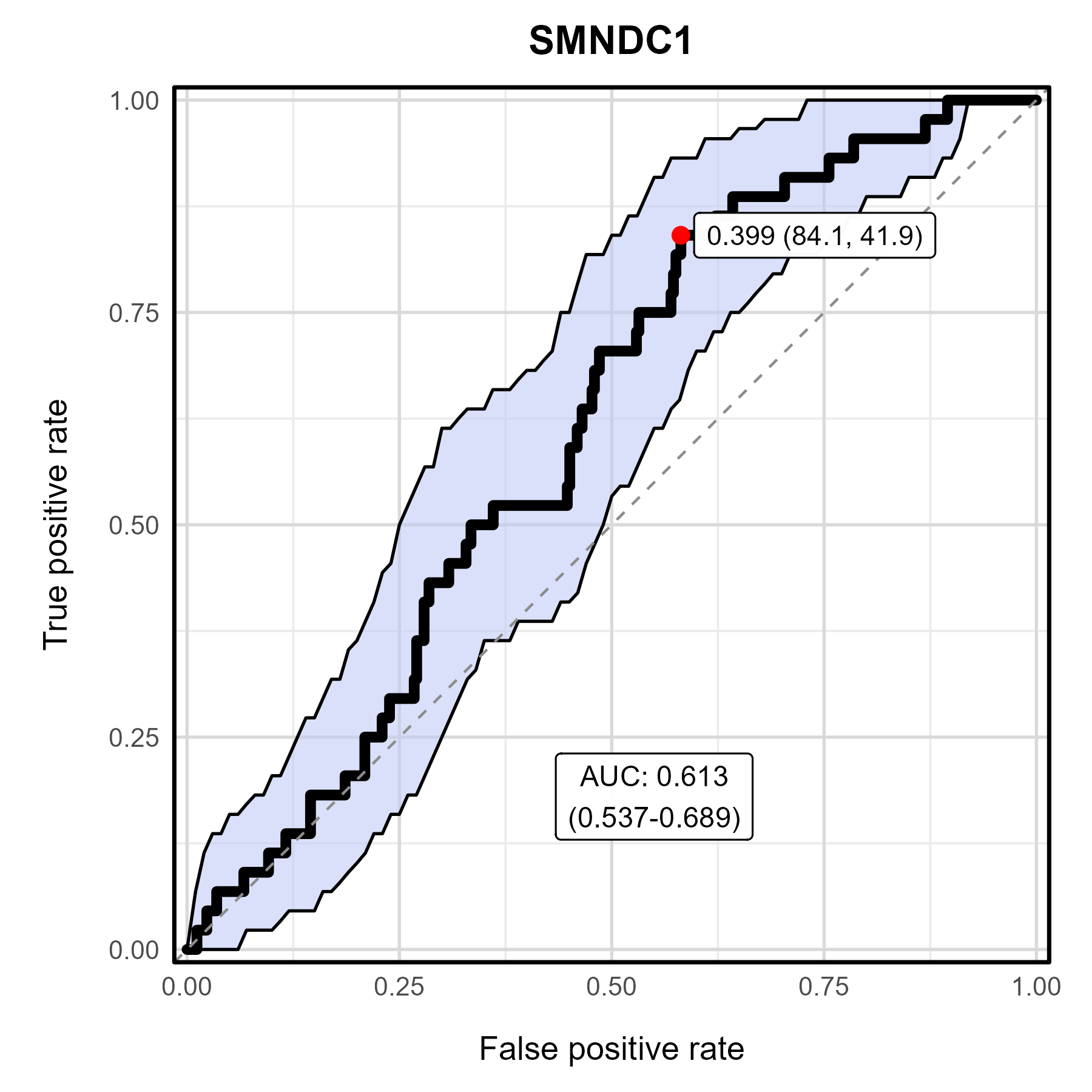

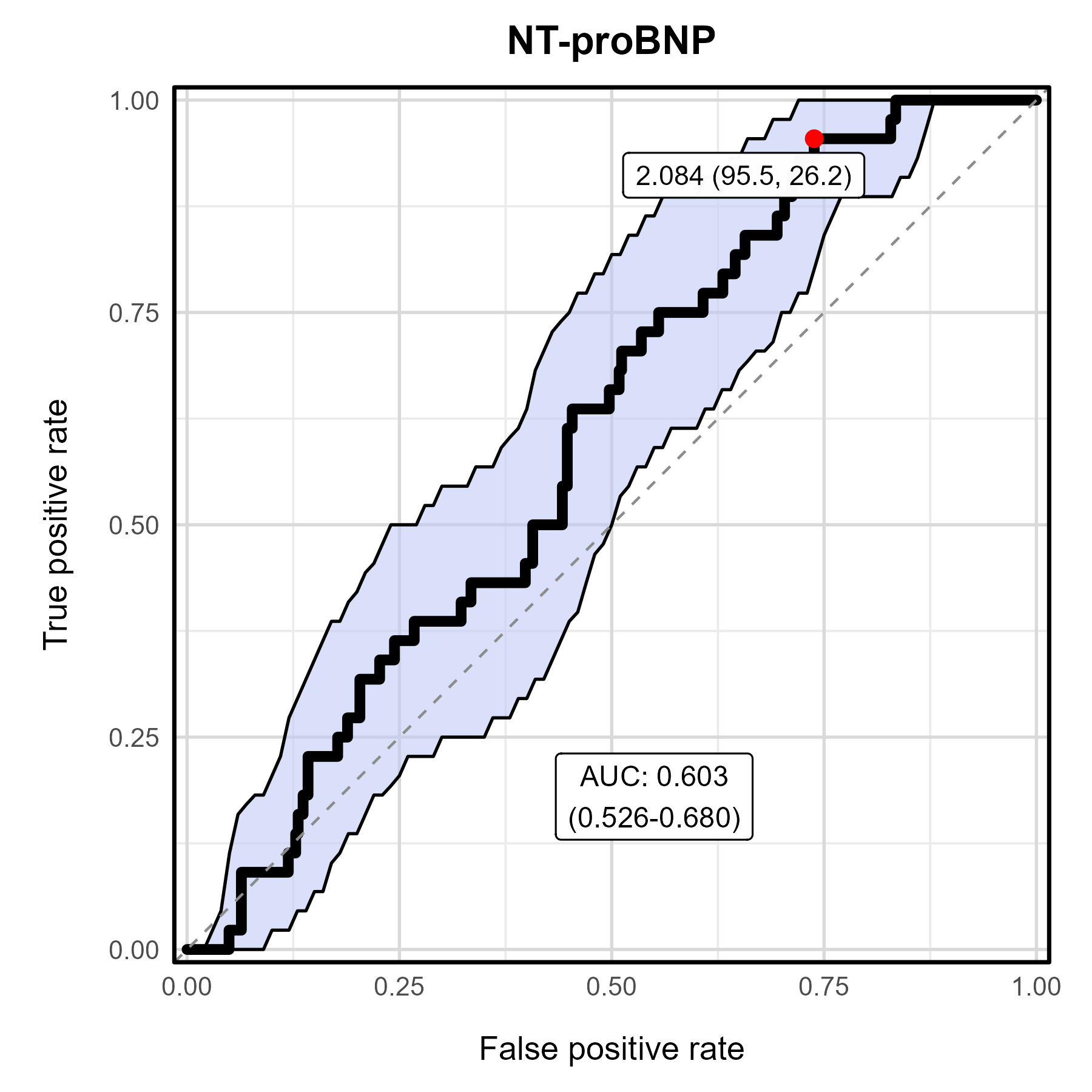

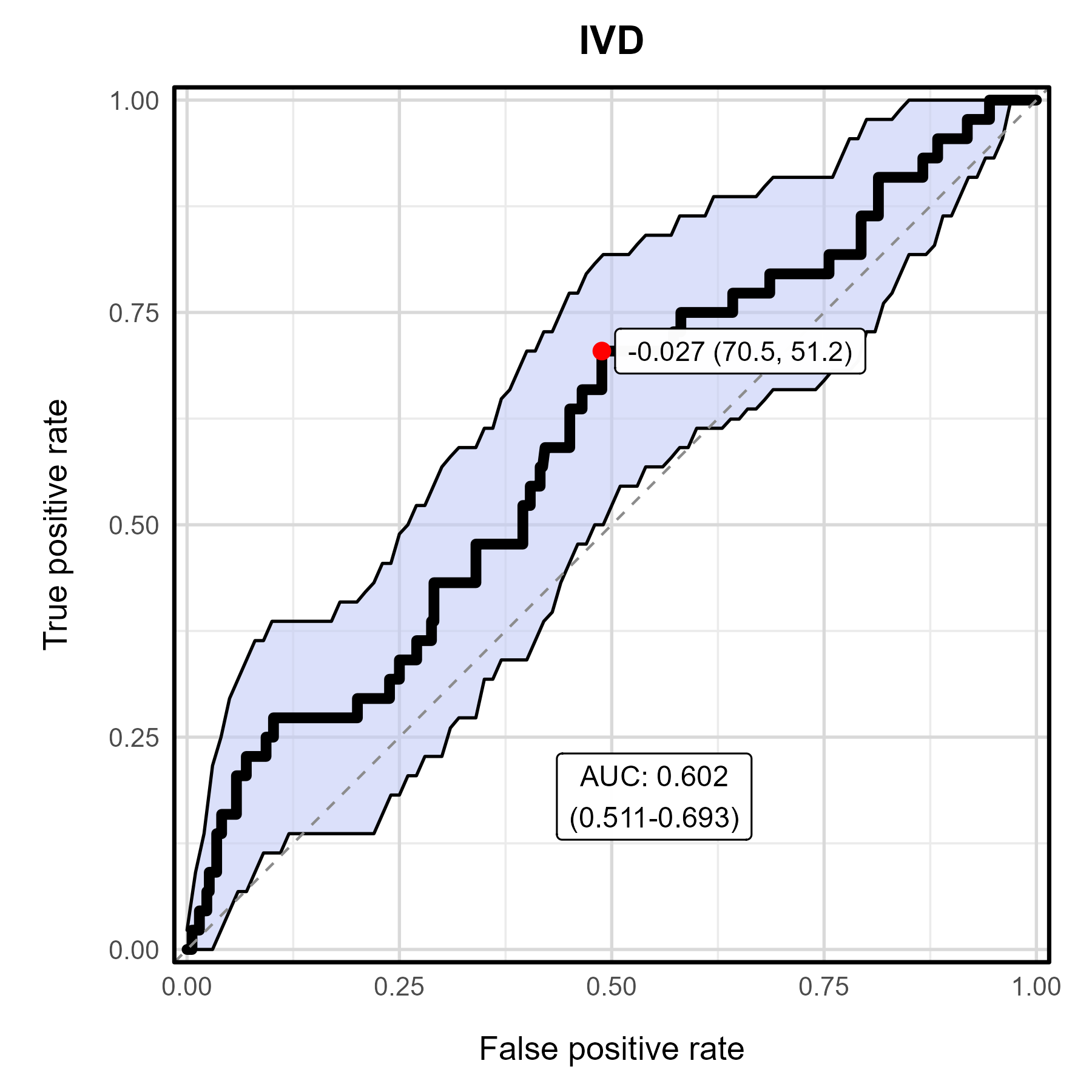


**Supplementary Figure S2.** **Receiver Operating Characteristic (ROC) curve analysis for proteins upregulated in ischemic stroke**. Each ROC curve includes the AUC value with 95% confidence intervals and the optimal cut-off points indicated by the red dot for S100A12, MNDA, PADI4, SMNDC1, NT-proBNP, and IVD in differentiating ischemic stroke from intracerebral hemorrhagic stroke patients.


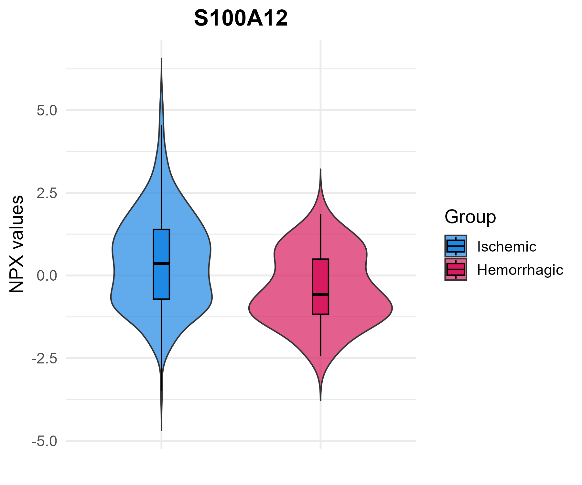

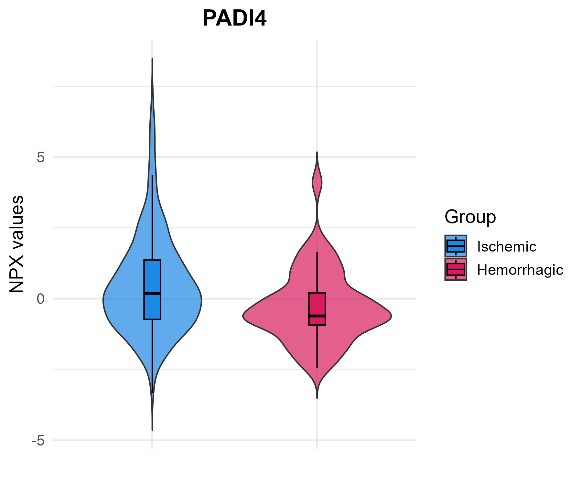

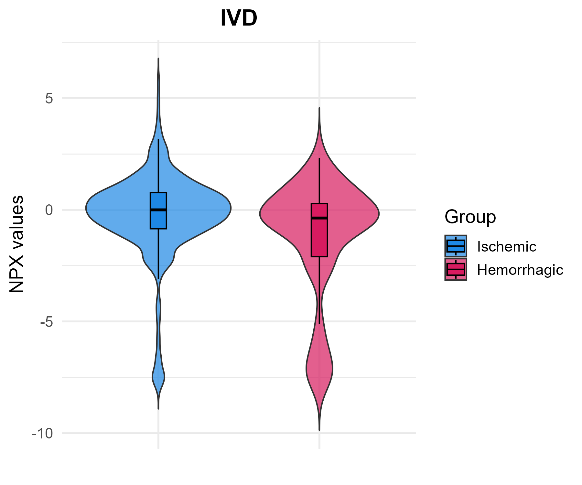

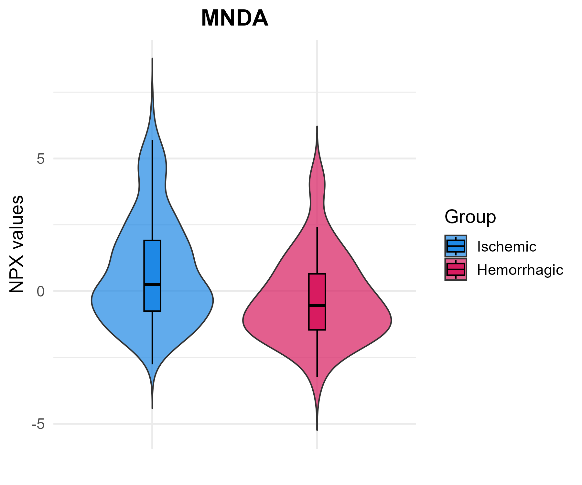

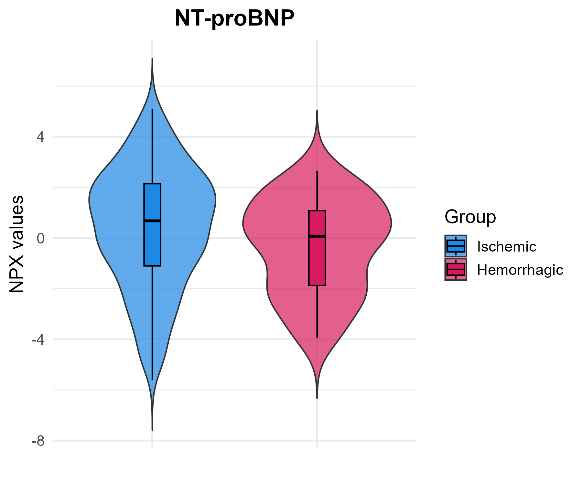

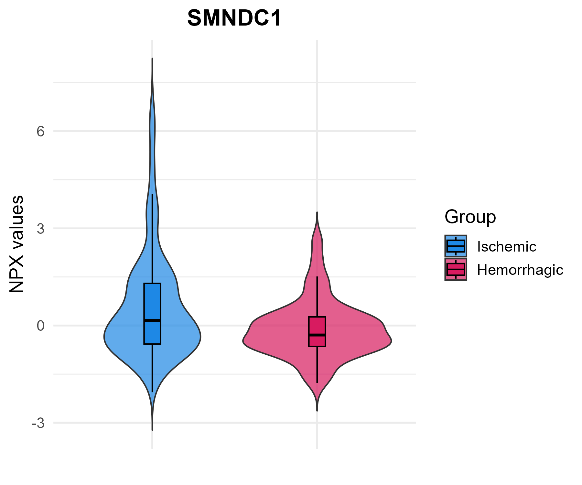


**Supplementary Figure S3. Violin plots showing Normalized Protein eXpression values of S100A12, MNDA, PADI4, SMNDC1, NT-proBNP, and IVD across ischemic stroke (blue) and intracerebral hemorrhage (red) groups.**


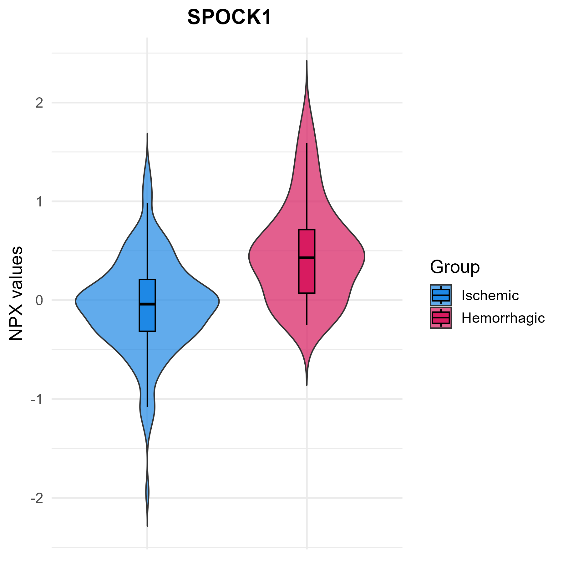

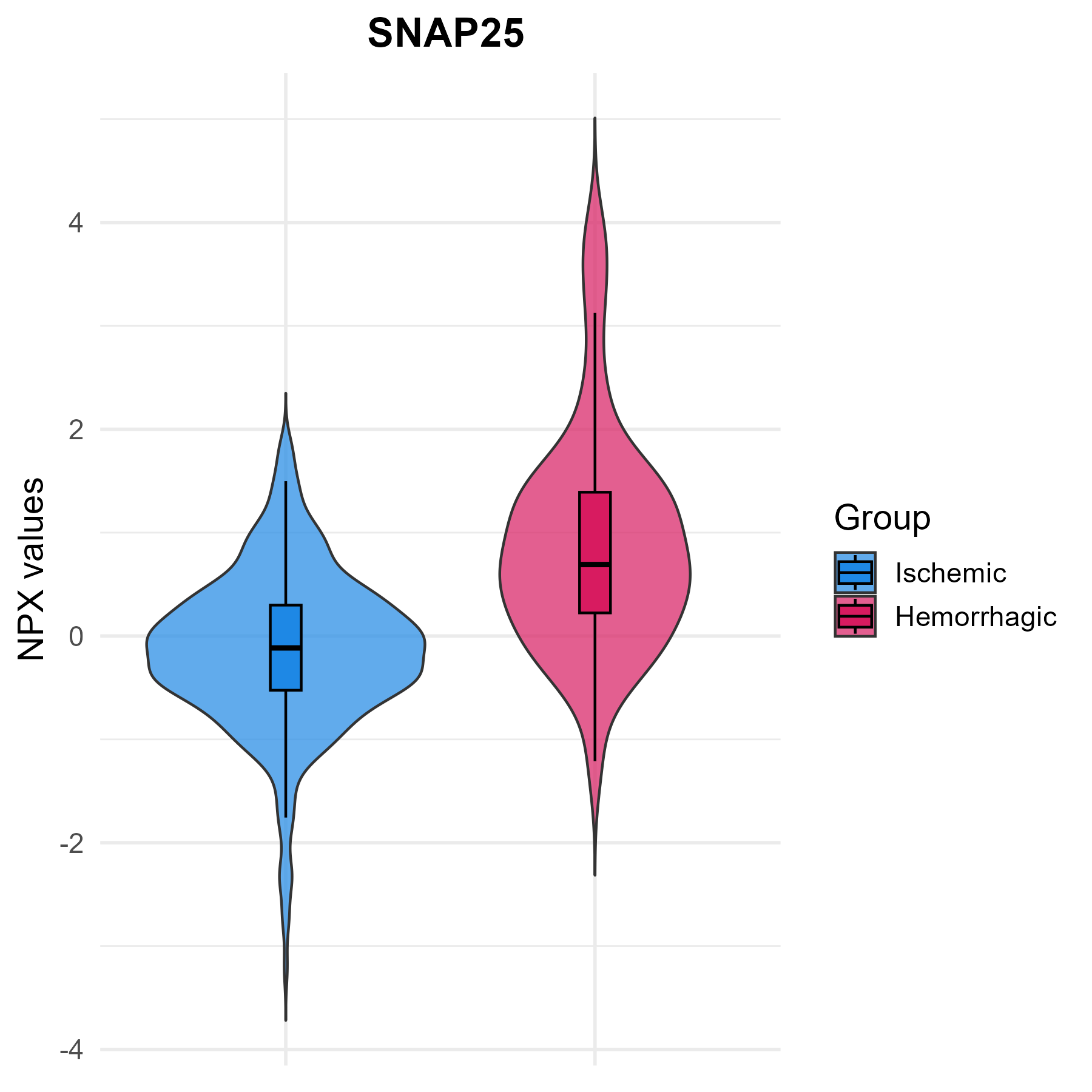

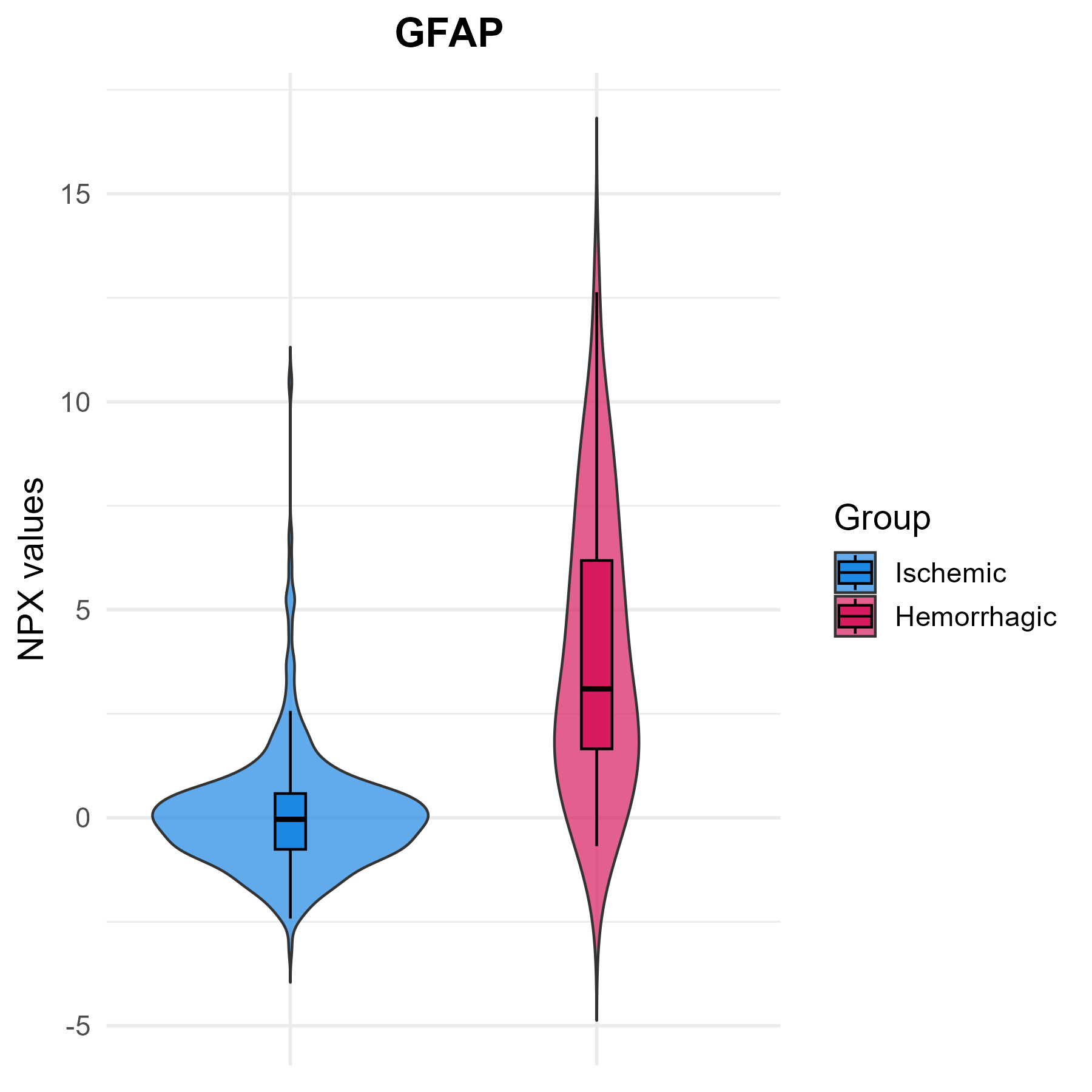

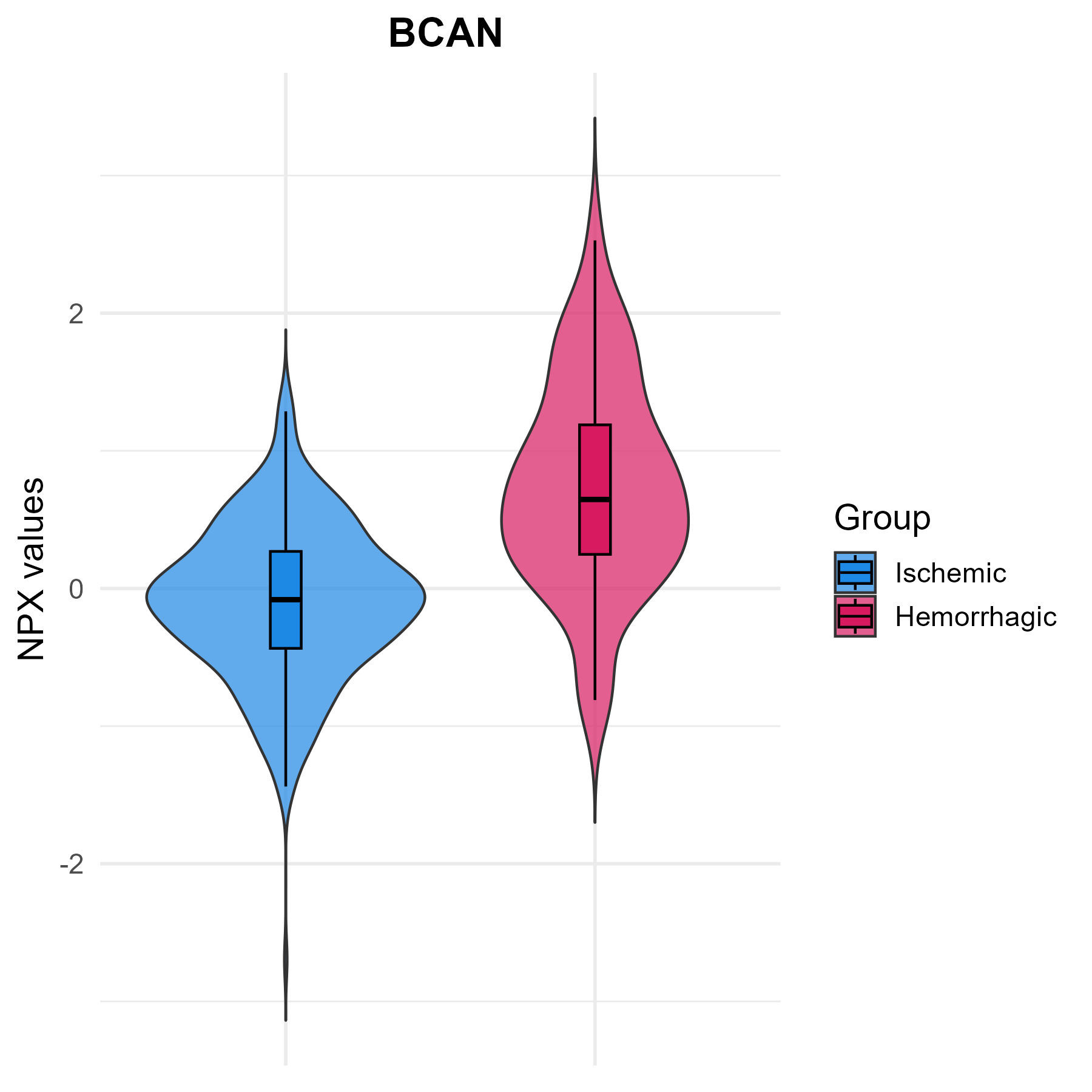


**Supplementary Figure S4. Violin plots showing normalized Normalized Protein eXpression values of GFAP, BCAN, SNAP25, and SPOCK1 across ischemic stroke (blue) and intracerebral hemorrhage (red) groups.**
